# Design, delivery, implementation, and evaluation of simulated clinical placements: a scoping review and evidence and gap map of the current state of evidence

**DOI:** 10.1186/s41077-026-00420-3

**Published:** 2026-02-18

**Authors:** Danielle Pollock, Charles Marley, Grace Holland, Sabira Hasanoff, Matthew Tieu, Adam Montagu, Jenniffer Paguio, Raju Kanakula, Carrie Price, Zachary Munn, Ellen Davies

**Affiliations:** 1 Health Evidence Synthesis, Recommendations and Impact (HESRI), School of Public Health, Faculty of Health and Medical Sciences, University of Adelaide, Adelaide, Australia; 2 School of Allied Health, Faculty of Health and Medical Sciences, The University of Adelaide, Adelaide, Australia; 3 Adelaide Health Simulation, Faculty of Health and Medical Sciences, The University of Adelaide, Level 2, 4 North Terrace, Adelaide, SA 5000 Australia; 4 College of Nursing, University of the Philippines Manila, Manila, Philippines; 5 School of Architecture, Design and Planning, University of Sydney, Sydney, NSW Australia; 6 Population Wellbeing and Environment Research Lab (PowerLab), Sydney, NSW Australia; 7 Independent Information Specialist, Baltimore, MD USA

## Abstract

**Background:**

Clinical placements allow health professions students to apply theoretical knowledge in clinical settings. For various economical, practical, and preferential reasons, some programs and institutions have trialled and established simulated clinical placements. This scoping review examines the evidence on the design, delivery, implementation, and evaluation of simulated clinical placements.

**Methods:**

We conducted a scoping review using JBI methods and reported according to the PRISMA-ScR. PubMed (NCBI), Embase (Embase.com), and CINAHL (EBSCO), PsycINFO (EBSCO) were searched. Reviewers screened title and abstracts and full-texts articles to assess for eligibility. Data were extracted and analysed with descriptive statistics and qualitative content analysis. Evidence and gap maps were developed to visualise the analysed data.

**Results:**

There were 131 documents included in this scoping review. Nursing (*n* = 77; 58.8%), physiotherapy (*n* = 10; 7.6%), and radiography (*n* = 9; 7.6%) were the most common fields where simulated clinical placements were trialled and established. Most studies originated the USA (*n* = 58; 44.3%), followed by Australia (*n* = 22; 16%), and the UK (*n* = 15; 11.5%). SCPs were experienced in-person (*n* = 57), or online (*n* = 46), with 18 documents reporting both in-person and online options. The percentage of replaced traditional clinical placement time most often ranged from between 0 and 50%, with seven documents reporting a replacement of between 76 and 100%. Several theoretical and conceptual frameworks were identified as being foundational in the establishment of simulated clinical placements. Learning outcomes, student outcomes, assessment types, regulatory and accreditation determinations and guidelines, resources (capital, human, technological, and student), costs and strengths, barriers, limitations and facilitators attributed to simulated clinical placements were identified.

**Conclusion:**

Simulated clinical placements are increasingly established to capitalise on the opportunities and affordances they offer to health professions students, particularly when traditional placements become less feasible. Given the acceleration of placements that are being undertaken in simulated rather than traditional health care services, it is likely they will continue to be established well into the future. There are significant opportunities to shape learner experiences and performance through how we use simulation techniques in simulated clinical placements for the purpose of building student readiness for practice as health professionals.

**Supplementary Information:**

The online version contains supplementary material available at 10.1186/s41077-026-00420-3.

## Background

Health systems around the globe are grappling with the challenge of receiving enough graduate health professionals who are *work ready* [5, 56]. Simultaneously, universities are contending with the challenge of finding enough appropriate placement positions in clinical contexts to graduate health professions students with sufficient work experience to be *work ready* [44, 64]. The tension between these two realities has resulted in shifting thoughts about clinical placement models: specifically, how, why, where, and with what resources these are undertaken. Clinical placement models are “theoretical structure[s] that guide educators and health professional students in their engagement with authentic clinical opportunities” [33, 45]. Traditionally, clinical placements have been undertaken in healthcare services, where learners have opportunities to observe and practice their professional skills under the direct supervision of licensed and registered clinicians and clinical educators [27]. Regulatory and accrediting bodies have mandated expectations relating to clinical placements, including, for some, the number of hours students must be present in a clinical context, before being able to register as a professional [27, 52].

The benefits of traditional clinical placements are wide-ranging. For instance, students can experience theory in practice, socialise in their chosen professions, interact with health professionals from other professions, disciplines and specialties, engage with patients, consumers and carers, and perform procedural tasks under supervision [33, 67]. However, there are also notable risks and adverse outcomes associated with traditional clinical placements [21]. These can be classified as psychological risks, risk of abuse, work-related physical risks, adverse supervision experiences, barriers to reporting inappropriate or unsafe work conditions, and barriers to competence [21]. Such risks not only impact students’ well-being but can also hinder their professional development [12, 28].

Alternatives to traditional clinical placement models have been trialled: of particular relevance to this paper is the application of various modalities of simulation to partially or fully replace the time and experiences considered requisite for graduation [8, 30, 31, 59]. Universities, accrediting bodies and health regulators have expanded their acceptance of alternatives to traditional clinical placement models in recognition of (1) the challenges of finding enough traditional clinical placements to accommodate students; (2) the risks and adverse outcomes associated with clinical placements and (3) the opportunities for optimisation and efficiencies afforded by retrenching from traditional clinical contexts into more controlled and controllable environments [6, 13, 30, 38, 44, 52].

The growing uptake of simulated clinical placements (SCPs) has occurred with varied inputs, expectations and requirements. Bespoke programs are being implemented for varying reasons, with varied resources and varied outcomes. The relatively rapid evolution and implementation of SCPs presents a gap in understanding for the broader health professions community who are wanting to know how they are operationalised, by and for whom, and under what circumstances. As programs seek to investigate the relevance and application of SCPs to their own programs, and as they consider investing in the human and capital resources required to establish SCPs, the lessons that can be learned from established programs become valuable. Decision makers are wanting to understand what SCPs are, why different programs decided to adopt this type of placement, what resources have been used, how SCPs have been evaluated, the financial implications of SCPs and the barriers and facilitators experienced in establishing successful SCP programs. Mapping this complex terrain is essential for informing evidence-based decision-making and future planning. This scoping review and evidence and gap map sought to identify and synthesise evidence related to characteristics of SCPs including their design, delivery modes, implementation and evaluation, with the broad aim of providing information to support decision-making and planning for SCPs.

## Methods

This scoping review was conducted in accordance with the JBI methodology for scoping reviews [46] and reported in line with the Preferred Reporting Items for Systematic Reviews and Meta-Analyses extension for Scoping Reviews (PRISMA-ScR) [60]. The evidence and gap map was developed following guidance from the Campbell Collaboration [63]. A protocol outlining the planned methods and approaches for this scoping review has been published [47].

### Review questions

What evidence exists to guide the design, implementation, delivery, and evaluation of SCPs?

### Sub-questions:

1. What are the characteristics and distinguishing features (e.g. theoretical underpinnings, learning outcomes, student assessments, hours allocated, resources used) of SCPs?
2. What definitions of SCPs have been proposed within the literature?
3. For what reasons have SCPs been implemented?
4. How have the regulatory, legislative, accreditation and qualification implications relating to SCPs been considered in the available evidence?
5. How have SCPs been integrated into the broader educational program and practical ecosystem where they are being established?
6. How have SCPs been evaluated?
7. What are the strengths, weaknesses, facilitators and barriers of SCPs?
8. What are the financial implications of SCPs?

### Eligibility criteria

#### Participants

This scoping review considered documents addressing SCPs for students undertaking tertiary education to become a health professional. In this review, tertiary education referred to undergraduate and postgraduate applied health degrees. This review used the definition of *health professional* developed by the International Standard Classification of Occupations (ISCO) [66], major group 2 (professionals), sub-major group (health professions) and the following minor categories in Table 1.

**Table 1:** Included health professions within this scoping review

ISCO-08 CODE	Examples
The following sub-major groups are included as well as their associated minor categories. • 221 – Medical Doctors • 222 – Nursing and Midwifery Professionals • 223 – Traditional and Complementary Medicine Professionals • 224 – Paramedical Practitioners • 225- Veterinarians • 226 – Other Health Professionals	Medical Practitioners; Nurses; Midwives; Dentists; Pharmacists; Physiotherapists; Dieticians; Nutritionists; Audiologists; Speech Therapists; Optometrists; Acupuncturist; Naturopath; Advanced care paramedic; Physical Therapist; Arts Therapist; Chiropractor; Occupational Therapist; Veterinarians, Osteopath and Podiatrist

#### Concept

This scoping review considered documents that have examined the design, implementation, delivery, and evaluation of SCPs. The SCPs may have been used as a sole replacement of traditional clinical placements or used in combination with traditional clinical placements.

#### Context

Documents which examined SCPs in tertiary (university) undergraduate, post-graduate, pre-registration and professional entry programs (including on campus, in-situ and virtual attendance) were included. There were no geographical limitations.

#### Types of documents

This scoping review included peer-reviewed articles which have examined SCPs for health professionals. They included documents of any study design, such as quantitative, qualitative, mixed-methods, case documents and reviews. Discussion pieces and commentaries which refer to the theoretical underpinnings and methodological approaches of SCPs were also included. Conference abstracts were assessed for eligibility and then the primary author was contacted to access either the study findings or the full report. If a full report was not available online, or from the authors of the abstract, it was exclude. There were no limitations based on publication date or language.

### Search strategy

A three-phase search strategy was developed by an information specialist (CP) to identify published and unpublished literature. An initial search of MEDLINE (Ovid) was conducted to identify relevant documents related to SCPs. From these identified records, the titles, abstracts, keywords, and subject headings were assessed to inform a full search strategy. The search strategy was reviewed by content experts (CM, MT, and ED) to ensure that all appropriate terms were identified. The search was then peer-reviewed by another medical librarian and assessed using the Peer Review of Electronic Search Strategies (PRESS) criteria [43].

The search strategy was adapted for each included database through Polyglot [10]. The following databases were searched: PubMed (NCBI), Embase (Embase.com), and CINAHL (EBSCO), PsycINFO (EBSCO). The search strategy was executed in these databases on May 2nd, 2024.

The journal titled *Focus on Health Professional Education* was hand searched by one author (SH) on the 5th of August 2024, as it is not currently indexed within academic databases. This journal was specifically included as it is a prominent professional journal managed by the Australian and New Zealand Association for Health Professional Educators (ANZAHPE). Relevant articles were screened by two reviewers in Covidence as per process for other screened documents. A supplemental search followed in which authors went through all the reviews that met eligibility criteria for this scoping review and collated the reference list of those. This list was screened for potentially relevant documents, and where necessary, authors were contacted for the full text.

DeepL Translator and Google Translate were used for documents in languages other than English to determine eligibility. If DeepL or Google Translate were unable to translate those documents, they were excluded.

### Study selection and screening

**Fig. 1:**
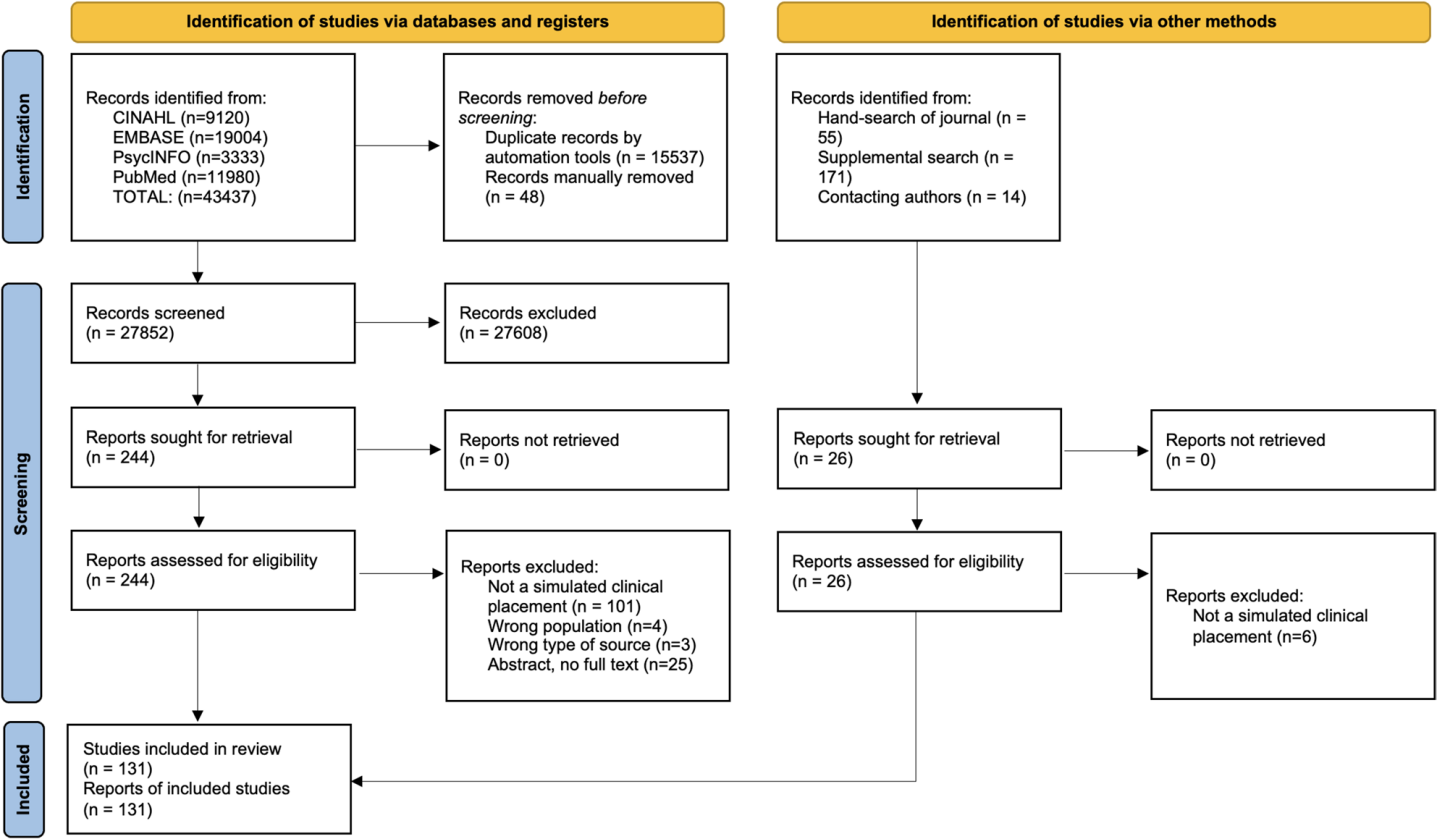
PRISMA Flowchart

After completion of the search, all identified documents were collated and uploaded into EndNote v.X20 (Clarivate Analytics, PA, USA). Duplicates were removed in Endnote and then again in the DeDuplicator tool in the Systematic Review Accelerator [9]. Deduplicated documents were imported into Covidence (Veritas Health Innovation, Melbourne, Australia). Screening was conducted in two stages: title and abstract screening and then full text screening. Piloting of the screening processes occurred before each of these two screening stages. The piloting allowed reviewers to independently screen documents against the eligibility criteria and then discuss their decisions with the whole group, ensuring a shared understanding of inclusion and exclusion parameters before each screening stage commenced. Piloting involved each reviewer independently screening ten titles and abstracts (prior to Stage 1 screening of title and abstracts) and ten full texts (prior to Stage 2 screening of full-texts) and then meeting to review screening decisions. In the first meeting of each stage, > 70% agreement was observed and screening for that stage commenced. Each document during title and abstract and full text screening was reviewed independently by two reviewers (DP, MD, RJ, GH, SH, MT, JP, ED). The search results and study inclusions are presented in a PRISMA flow diagram (Fig. 1). Reasons for excluding full text documents that did not meet the inclusion criteria were recorded and are reported in Supplementary File 1.

Weekly meetings were held to discuss conflicts amongst reviewers in their screening decisions. Any disagreements that arose between the reviewers at each stage of the selection process were resolved through discussion and majority consensus.

### Data extraction and coding

Extraction of the included documents occurred in EppiReviewer [58] using a standardised data extraction form and data dictionary specifically designed for this review. This data dictionary was co-developed by the team to support shared understanding of the concepts and terms relevant to the study. Prior to starting data extraction, reviewers (DP, GH, RJ, SH, MT, JP, ED) were asked to pilot the data extraction process by extracting data from five documents that varied in methods and/or participant characteristics. During piloting, the extraction form was calibrated based on the feedback received by the reviewers to ensure that all necessary information was being collected. Once piloting concluded, each document was extracted independently by two reviewers (DP, GH, RJ, SH, MT, JP, ED). As the nature and complexity of the evidence emerged during the initial stages of extraction, the data extraction form required alterations. These alterations were designed to support a better shared understanding of what content needed to be extracted. Discrepancies between the extraction table provided in the protocol and the one altered during this study have been listed in Supplementary File 1. Discrepancies between individual extraction of the same document were resolved with an additional reviewer (DP, SH, GH) reconciling differences.

### Data analysis and presentation

To address the review questions, a mixture of descriptive statistics, such as frequency counts and percentages, and inductive basic qualitative content analysis, as described by Pollock et al., [48], were used.

Inductive basic qualitative content analysis involves four stages: (1) open coding; (2) development of the coding framework; (3) extraction and organising; and (4) categorisation [48]. During the initial stages of analysing the extracted data, reviewers (DP, SH, GH, ED, MT, CM) noted their thoughts and potential categories which described the data. These notes were used to develop the coding framework, which was then reviewed by a second reviewer (ED). Once the coding framework had been developed and reviewed, the extracted data were organised into their relevant category. These extractions were checked by a second reviewer (ED), and any suggested changes to coding or categorisation were discussed. All documents were coded, and the results are presented in two evidence maps.

## Results

A search of four databases yielded 43,437 records. After removing duplicates, 27,852 records remained for title and abstract screening. After excluding irrelevant records, 244 were eligible for full text screening. After review, 133 records were excluded as they did not describe SCPs (*n* = 101), they included the wrong population (*n* = 4), wrong type of source (news items) (*n* = 3), or were abstracts with no associated full text article (*n* = 25). The hand search of the academic journal previously specified and supplemental search identified 240 additional records, with 26 reports sought for retrieval and screening, and six removed as they did not report SCPs. Details are provided in the Supplementary File 1.

In total, 131 reports met the eligibility criteria and were included in this review (111 were identified through medical databases, 20 from the hand and supplemental searching). The results of the search and inclusion are presented in a PRISMA 2020 flow diagram (Fig. 1), and extractions are available in Supplementary File 2.

The completed PRISMA Checklist, list of deviations from our protocol, the full search strategy, the Peer Review of Electronic Search Strategies (PRESS) assessment, data dictionary, list of documents excluded at full text screening and reasons for exclusion, data extraction table, and the details of included documents are available in Supplementary File 1. Two different representations of our findings in the form of evidence and gap maps are publicly available through the following links: (https://simulatedclinicalplacementsegm.netlify.app/; https://simulatedclinicalplacementegm1.netlify.app/). Links to additional files and project registration can be located on the Open Science Framework website (https://osf.io/q39am/).

### Characteristics of included documents

The included documents differed in terms of study focus and design, field, year of publication, sample size and geographic location (Table 2). The most common fields represented in this scoping review were nursing (*n* = 77; 58.8%), physiotherapy (*n* = 10; 7.6%), and radiography (*n* = 9; 7.6%). Countries from where SCPs were reported included the USA (*n* = 58; 44.3%), Australia (*n* = 22; 16%), and the UK (*n* = 15; 11.5%). The most frequent study designs included case studies (*n* = 18; 13.7%), prospective cohort studies (*n* = 14; 10.7), cross-sectional studies (*n* = 11; 8.4%), and quasi-experimental studies (*n* = 11; 8.4%). The most popular methods of data collection were surveys (*n* = 64; 48.9%), student assessments (*n* = 18; 13.7%), and secondary evidence reports (i.e., reviews) (*n* = 14; 10.7%). The sample sizes included studies with as few as 20 participants (*n* = 12; 6.1%) and as many as 2000 participants (*n* = 1; 0.8%).

**Table 2:** Document characteristics

Document Characteristics	n	%	Document Characteristics	n	%
Sample Size			Field		
0–20	12	6.1	Health professionals, not-specific	8	6.1
20–50	29	17.6	Dietetics	1	0.8
50–100	24	9.2	Genetic Counselling	1	0.8
100–500	27	22.1	Medicine	6	4.6
500–1000	7	18.3	Myotherapy	1	0.8
1000–2000	1	0.8	Nursing	77	58.8
Not Mentioned	8	6.1	Occupational Therapy	6	4.6
Not Applicable	23	17.6	Osteopathy	2	1.5
Study Design			Paramedicine	1	0.8
Randomised Control Trial	9	6.9	Pharmacy	1	0.8
Quasi-experimental	11	8.4	Physiotherapy	10	7.6
Pre-Post study design	10	7.6	Radiography	9	6.9
Cross-sectional	11	8.4	Speech Pathology	8	6.1
Prospective cohort	14	10.7	**Country**		
Retrospective cohort	5	3.8	Australia	21	16.0
Case-control	1	0.8	Canada	5	3.8
Qualitative	13	9.9	China	1	0.8
Case study	18	13.7	Germany	1	0.8
Cost analysis	1	0.8	Indonesia	1	0.8
Delphi	1	0.8	Japan	1	0.8
Discussion	9	6.9	Norway	1	0.8
Economic evaluation	1	0.8	Oman	1	0.8
Mixed Methods	13	9.9	Saudi Arabia	1	0.8
Review	12	9.2	South Korea	1	0.8
Editorial	2	1.5	Qatar	1	0.8
Method of Data Collection			United Kingdom	15	11.5
Focus groups	9	6.9	United States of America	58	44.3
Interviews	12	9.2	Multiple Countries	2	1.5
Observation	7	5.3	NA	21	16.0
Secondary evidence	14	10.7	**Year**		
Student assessments	37	28.2	1995–2000	2	1.5
Survey	64	48.9	2001–2010	6	4.6
Workshops	0	0.0	2011–2015	17	13.0
No methods described	4	3.1	2016–2020	36	27.5
Not applicable	15	11.5	2021–2024	70	53.4
Other	7	5.3			

The earliest eligible documents were published in 1996 (*n* = 2; 1.5%). As seen in Table 2, there was a gradual increase in publications until 2020, after which there was a sharp increase in the number of published papers.

### Evidence and gap map

**Fig. 2:**
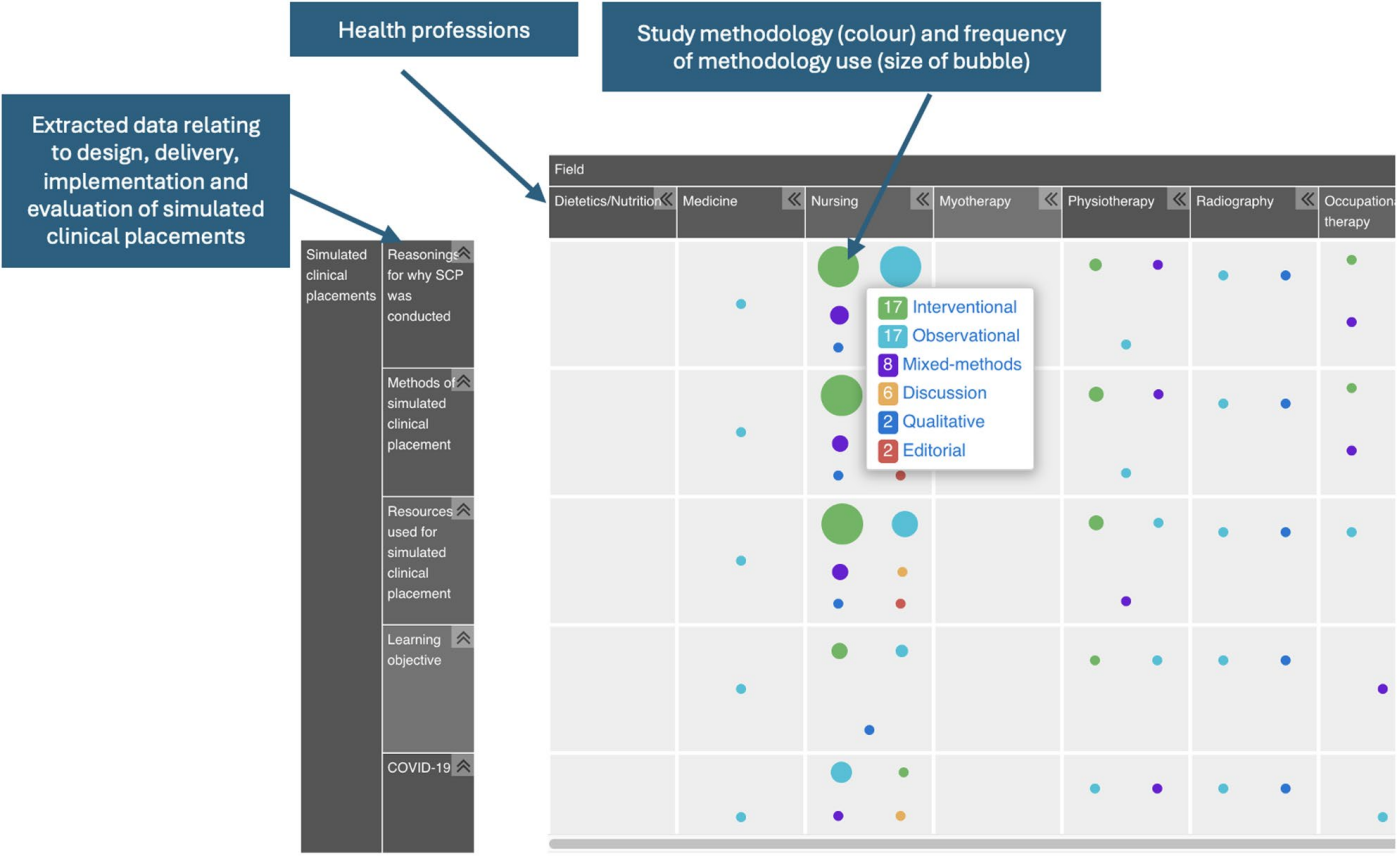
Example of the Evidence and Gap Map of SCP evidence

We developed an evidence and gap map of all the documents included in this review to provide a visual representation of the evidence relating to SCPs. Figure 2 provides a snapshot of the interactive map. The map has been organised by health field and is segmented by study design. Users can click on the colour-coded study designs to obtain a list of all the relevant documents which are coded to that section.

### Design of SCPs

#### Defining and framing SCPs

Eight documents provided definitions for SCPs; these are presented in Table 3. There was limited consistency in these identified definitions. There were no instances of a definition being repeated. However, there were some shared concepts in the identified definitions, including:

1. Virtual and SCPs share a common goal with traditional clinical placements of scaffolding student learning;
2. Patients are simulated;
3. SCPs use various simulation resources to replicate clinical placement environments and interactions;
4. Simulated interactions should be as authentic and complex as they are on traditional clinical placements;
5. SCPs share common assessable components with traditional clinical placements.

**Table 3:** Definitions of SCP

Authors (year of publication)	Definition (verbatim)	Key features of definition
Brook et al., [7]	Virtual placements, or placements conducted outside of the clinical environment that seek to scaffold student learning within the clinical environment.	- Virtual and SCPs share a common goal with traditional clinical placements to scaffold student learning
Cornelius, [11]	A learning experience where the nursing student utilizes knowledge of nursing care to interact with a mannequin (sic.) that exhibits the signs and symptoms of a medical condition.	- Patients are simulated (in this case with a manikin)
Grant et al., [22]	An experience offering high levels of authenticity and complexity which should directly replicate real placement interactions and should be assessed in a similar manner.	- Simulated interactions should be as authentic and complex as they are on traditional clinical placements - SCPs share assessable components with traditional placement
Hill et al., [25]	An alternative learning environment to clinical placements for the development of professional competencies.	- Provide opportunities for developing professional competencies
Ketterer et al., [36]	Simulation placements include a range of simulation resources to provide students with an experience as closely matched to clinical placement as possible.	- SCPs use various simulation resources to replicate clinical placement environments and interactions
Massias, [41]	High-fidelity simulated hospital experience is direct student contact with high-fidelity mannequins (sic.) in a simulated acute care setting. A simulation mimics the reality of the clinical environment and [uses] techniques such as role-playing and mannequins (sic.).	- SCPs use various simulation resources to replicate clinical placement environments and interactions
Morgan et al., [44]	Simulated practice is an educational method, using a variety of modalities to support student learning in developing their knowledge, behaviours and skills, with the opportunity for repetition, feedback, evaluation and reflection to achieve their programme outcomes. … Simulation is a technique to replace or amplify real experience with guided experience, or to evoke or replicate practice in an interactive manner. … As such there are a variety of different simulation methods that can be used, of which this is virtual practice.	- SCPs use various simulation resources to replicate clinical placement environments and interactions - SCPs provide learning opportunities where students can receive feedback on their performance - Virtual reality (amongst other modalities) can be used to replace or amplify traditional clinical placement experiences
Samson et al., [54]	[Virtual Simulated Placement] is defined in this research as a computer-generated version of a practice placement.	- Traditional placement environment is replaced with a virtual environment

#### Theoretical underpinnings and conceptual frameworks

As seen in Table 4, the theories and conceptual frameworks that were used in the design of SCPs were described in 26 documents. Nine theoretical lenses were identified, with experiential learning, social constructivist, and cognitive-behavioural theories the most frequently reported. Thirty-four documents reported the conceptual frameworks that were used in the design of SCPs. Nine conceptual frameworks were identified, with the most frequent being the National League for Nursing’s NLN/Jeffries Simulation Framework (*n* = 16).

**Table 4:** Theoretical underpinnings and conceptual frameworks

Theoretical Underpinnings	n	Conceptual Framework	n
Experiential Learning	14	NLN/Jeffries Simulation Framework	16
Social Constructivism	8	Kerns’ Model of Curriculum Development	5
Behavioural/ Cognitive	4	Simulation-based Learning Frameworks	5
Situated Learning	3	Tanner’s Clinical Judgement Model	2
Andragogy (Adult Learning)	3	Miller’s Pyramid of Clinical Competency	2
Skill Acquisition/Expertise	3	Kirkpatrick’s Model	2
Blended Learning	1	Schon’s Reflective Practice Model	2
Reflective Learning	1	Blooms Taxonomy	1
Problem- Based Learning	1	Normative Ethics	1

#### Intended learning outcomes

There were 34 documents which reported intended learning outcomes (ILOs). ILOs were grouped into three categories: *behavioural*,* social and cognitive skills*,* procedural and technical skills*, and *theory to practice*. A description of each category, an extracted example, and the number of ILOs coded to each category is presented in Table 5. Where relevant, ILOs were assigned to multiple categories.

**Table 5:** Intended learning outcomes

Category	Description	Extracted example	*n*
Behavioural, social and cognitive skills	Intended learning outcomes that primarily require learners to demonstrate behavioural, social and/or cognitive skills.	Communicate effectively with the patient and family in a simulated obstetric emergency scenario. Actively participate and engage as a healthcare team member by demonstrating mutual respect, understanding, and values to meet patient care needs [42].	176
Procedural and technical skills	Intended learning outcomes that primarily require learners to demonstrate procedural and/or technical.	Effectively conduct a clinical swallowing examination including the use of compensatory strategies [24].	103
Theory to practice	Intended learning outcomes that primarily require learners to link theoretical knowledge to practice.	Evaluate the patient/client examination-information, using clinical reasoning, and evidence-based practice including outcomes assessment and select an appropriate diagnosis and prognosis [14].	53

#### Integration into the broader educational program

There were 60 documents that described how SCPs were integrated into broader educational programs. The extracted data were organised into six categories: *Curriculum integration*; *Integration guidance; Innovation and future direction; Resource optimisations*; *Blended learning model*; and *Regulatory integration*. Further details for each category and an example of extracted data are described in Table 6.

**Table 6:** Integration into the curriculum

Category	Description	Example of extracted data	*n*
Curriculum integration	Refers to how simulation is embedded within academic programs to complement or replace traditional learning approaches, ensuring alignment with curriculum goals and/or learning outcomes.	Others liked the concept of simulation but recommended that it may be best utilised as part of the academic teaching program rather than taking time away from placement in the clinical setting: simulation week might be better placed more as a university focus thing rather than taking it from the clinical, a way of tying in university and the clinical situation rather than taking away from their time on the clinical placement Blackford et al., [3].	23
Integration guidance	Provides information/support for integration into educational ecosystem without clear evidence of actual integration.	The future of HS in curriculum development is in its infancy and will not replace the traditional clinical environment. University faculty must stress to administration the importance of HS to seek funding sources for its incorporation into the curriculum. Once available, it is the responsibility of creative faculty members to incorporate simulation experiences throughout the curriculum. The nursing profession and health care community will reap the benefits of increased competence and improved patient safety [26].	18
Innovation and future direction	Simulation may not be yet integrated, but study highlights the potential role of simulation in driving educational innovations, addressing emerging challenges, or exploring future applications in healthcare education, thus supporting integration.	Partial replacement of therapeutic radiography clinical placement weeks is feasible and therefore could reduce clinical training burden on both departments and students. Future investigations into the impact of interventions on clinical skills should be based on performance data and not rely on self-reported confidence levels. More work is also needed to identify the role of simulated placements with regard to advanced learners and complex skills development. Ketterer et al., [36].	16
Resource optimisation	Refers to how simulation does/could address practical challenges, i.e., placement shortages, resource constraints, or optimisation of educational delivery.	As students return to patient care in EDs, educators must be prepared for the possibility that volumes will not be sufficient to ensure a robust educational experience without supplemental material taught in a nonclinical setting. Having a prepared contingency plan for an online-based curriculum for students who are experiencing interruptions in their education due to quarantine, isolation, or other unforeseen events or absences not related to COVID-19 is a beneficial resource. A bank of chief complaint-based activities and resources can help auGHent an in-person emergency department clerkship if there is a specific, patient encounter deficiency identified by the student or educator. Redinger and Greene, [50]	11
Blended learning model	Refers to a focus on hybrid approaches combining simulation with traditional placements/teaching to enhance learning outcomes, promote engagement, and offer a balanced educational experience.	Virtual simulation provides opportunities for teaching and learning nursing concepts, practicing clinical skills in a safe environment, integrate decision-making and clinical reasoning which leads to fulfillment of clinical core competencies. Nursing faculty can assist students to translate classroom didactic concepts and connect them to the practice setting, bridging core and clinical competencies. Sharoff, [55]	8
Regulatory integration	Refers to how simulation aligns with, or is, shaped by regulatory frameworks, licensing requirements, and/or accreditation standards, ensuring compliance with professional and institutional guidelines.	In Singapore, for professional licensing of registered nurses, special approval was obtained from Singapore’s nursing regulatory board to replace 160 consolidated clinical practice hours with 80 h of simulation-based learning during the pandemic, to develop graduating students’ competence in patient care management and fulfil the accreditation requirements. Thereafter, graduating students were permitted to resume clinical postings. Anggraini et al., [1]	6

### Delivery of SCPs

#### Simulation modalities

**Fig. 3:**
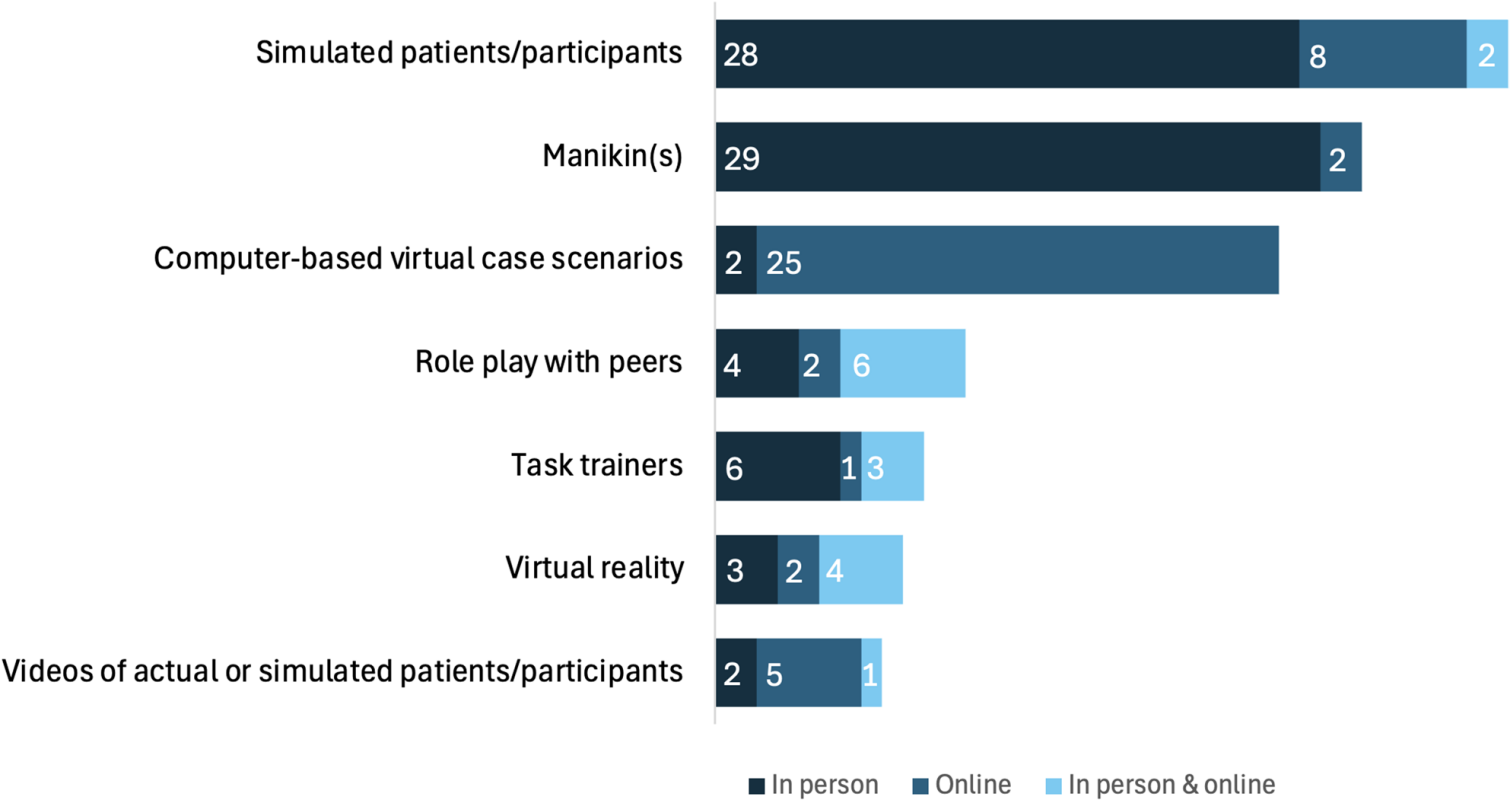
Simulation modalities used in SCPs

SCPs were typically experienced in-person (*n* = 57), or online (*n* = 46), with 18 documents reporting both in-person and online options. As seen in Fig. 3a variety of techniques and modalities of simulation were used in SCPs, including simulated patients (SPs), manikins, computer-based virtual case scenarios, role play with peers, task trainers, virtual reality, and videos. It was more common for in-person SCPs to use a mixture of modalities when compared to online SCPs.

#### Hours allocated to SCPs

We could not synthesise data relating to the range of hours allocated to SCPs because there was no reporting consistency across sources. Ninety-seven documents reported the number of hours students attended, or were projected to attend SCPs. These can be separated based on whether the document described a SCP (*n* = 79) or if it articulated recommendations or expert opinion relating to the number of SCP hours that could replace traditional clinical placements (*n* = 21). The length of time students spent attending SCPs was reported in hours (*n* = 59), weeks (*n* = 26), days (*n* = 18), percentage (*n* = 21), ratio (*n* = 11) or years (*n* = 1).

#### Percentage of traditional clinical placements replaced with SCPs

**Fig. 4:**
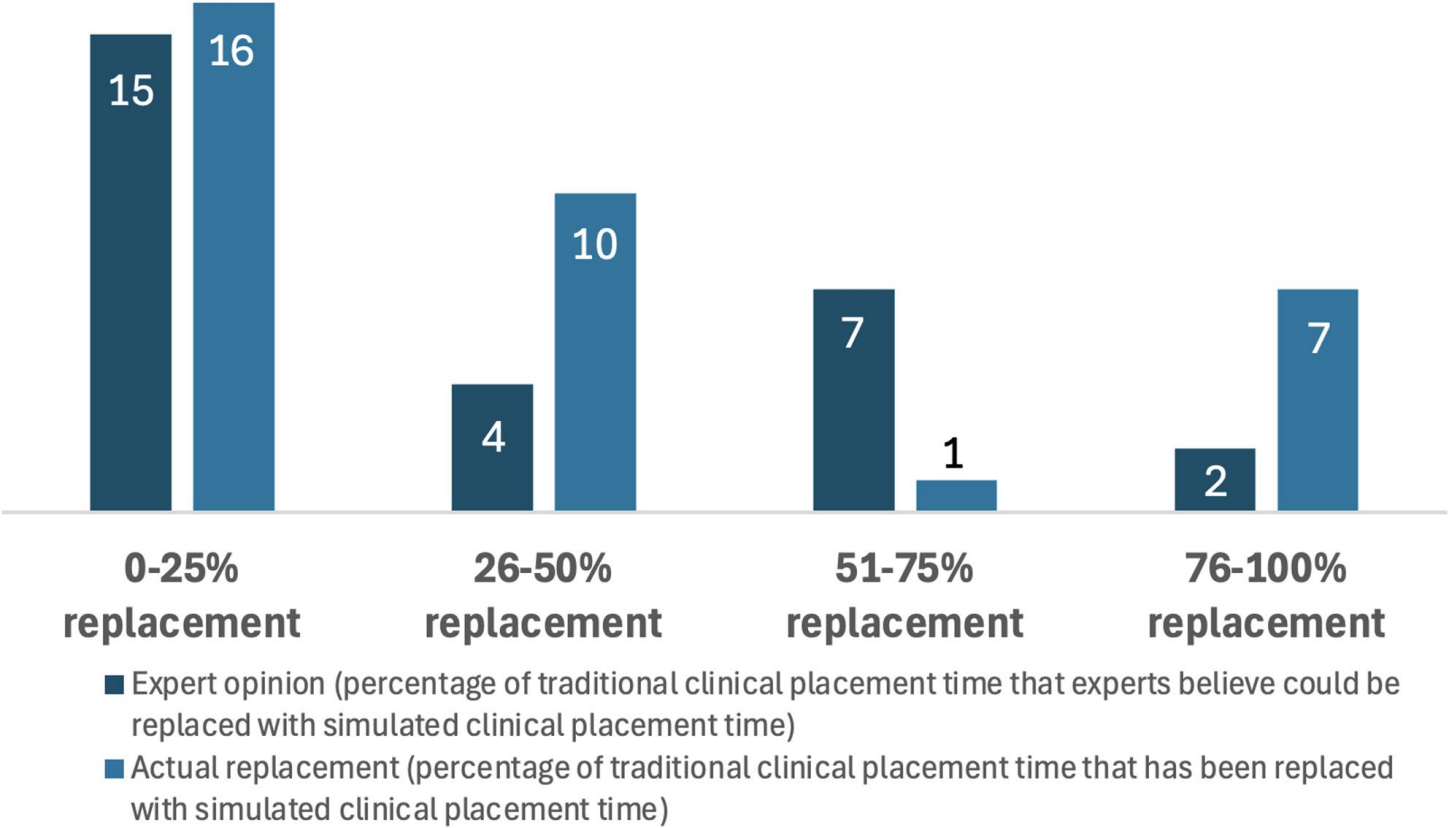
Percentage of SCP

Documents often stipulated that a percentage of traditional clinical placements could be, or were, replaced with SCPs. These percentages were reported as either expert opinion of what could feasibly be permitted in specific educational programs (*n* = 25) or what occurred (*n* = 28). Documents that reported expert opinion vs. actual replacement were separated to highlight any potential differences as identified in Fig. 4. Expert opinion reported that up to 50% of a placement could be replaced by simulation. In practice, the replacement percentage was often between 0 and 50%, with 7 documents reporting a replacement of between 76 and 100% of total placement hours with SCPs.

#### Student assessment in SCPs

There were 65 documents that reported conducting student assessments in SCPs. Documents reported various methods for conducting summative and formative assessments. Our review identified 20 different validated assessment tools used to assess student outcomes. Tools that were used to assess students in Objective Structured Clinical Exam (OSCE), quizzes, questionnaires and reflective essays were not always explicitly described. The full list of validated tools and assessment types can be seen in Table 7.

**Table 7:** Student assessment details in SCP

Validated and/or industry assessment tools	Types of assessment
1. Assessment of Physiotherapy Practice (APP) 2. ATI Adult Medical-Surgical Proctored Assessment 3. Assessment Technologies Institute (ATI) Registered Nurse (RN) Comprehensive Predictor 4. Assessment Technologies Institute (ATI) RN Mental Health Mastery Examination 5. Clinical Performance Instrument 6. Competence Assessment in Speech Pathology (COMPASS) 7. Creighton Competency Evaluation Instrument (CCEI) 8. Critical Thinking Process Test (CTPT) 9. Health Education Systems Incorporated (HESI) medical-surgical specialty exam 10. Health Science Reasoning Test (HSRT) 11. Lasater’s Clinical JudGHent Rubric 12. National Board for Certification in Occupational Therapy (NBCOT) 13. National Council Licensing Exam for Registered Nurses (NCLEX-RN) 14. Next Generation National Council Licensure Exam (NCLEX) 15. Physical Therapist Clinical Performance Instrument 16. Quint Levelled Clinical Competency Tool 17. Standardised Patient Interview Rating Scale 18. Standardized Tools for the Assessment of Radiology Students (STARS) 19. Student Oral Case Analysis (SOCA) 20. Student Practice Evaluation Form Revised Edition (SPEF-R)	1. Attitude and performance assessment 2. Clinical performance evaluations 3. Computer generated score 4. Formative evaluation from faculty on performance 5. Graded scenarios 6. Knowledge test 7. Objective Structured Clinical Exam (OSCE) 8. Online quizzes 9. Online questionnaire 10. Multiple choice quiz/text 11. Performance assessment 12. Performance rubrics 13. Practical examination 14. Reflective essay 15. Simulation evaluations 16. Virtual clinical assignments 17. Workbook 18. Written assignments 19. Written examination

### Implementation of SCPs

From the 131 included documents, 127 described reasons why SCPs were conducted. These were grouped into six categories: *limited capacity in clinical settings to provide placements; to protect faculty*,* clinical staff*,* students and patients during the COVID-19 pandemic; standardised and predictable approach for meeting student competency requirements; providing a safer environment in which to practice clinical skills for students*,* faculty*,* and patients; cost effective and cost saving when compared to traditional clinical placements and; to meet student learning requirements*. Details and illustrations for each of these categories are seen in Table 8.

**Table 8:** Reasons why SCPs were implemented

Category	n	Extracted example
Limited capacity in clinical settings to provide placements	52	*Currently*,* academic leaders are facing the challenges of an increasing number of students*,* the difficulty of recruiting teachers and preceptors to accompany nursing students*,* and fewer clinical settings that can accommodate many interns at once. To come to terms with these changes*,* which include fewer resources*,* the idea of replacing clinical hours with simulation has emerged” (* [39], p. *152).*
To protect faculty, clinical staff, students and patients during the COVID-19 pandemic	47	*“With the advent of the COVID-19 pandemic in 2020*,* and the limited supply of real fieldwork opportunities*,* there became an increased urgency to create a simulation-based model that incorporated the entire [penultimate year of study] student cohort in a cost-effective way. During the pandemic*,* this cohort was not the only group impacted by the paucity of traditional fieldwork placements. Physiotherapy students in their final year of study… faced delayed graduation with incomplete clinical training” (* [15], p. *2).*
Standardised and predictable approach for meeting student competency requirements	16	*“Simulation can also provide standardized clinical experiences to meet curricular needs when the experience was not being met at a traditional clinical site” (* [57], p. *3).*
Providing a safer environment in which to practice clinical skills for students, faculty, and patients	6	*“In simulation*,* students learn in a realistic clinical environment where they practise skills without risk to patients and then apply these skills in placement” (* [2], p. *293).*
Cost effective and cost saving when compared to traditional clinical placements	5	*“Although the initial funded model was costly*,* the university recognized the merit of the placement in terms of student outcomes that were found to be comparable to traditional real preparatory placements” (* [15], p. *2)*
To meet student learning requirements	1	*“In Qatar*,* a predominantly Muslim country*,* social and cultural norms prohibit male BN students from practicing in maternity settings. However*,* male physician and paramedic students*,* although limited in scope*,* are permitted to practice in this setting. All BN students at the University of Calgary in Qatar (UCQ) are required to complete a maternity clinical in order to meet program outcomes. While male students who choose to work in Qatar do not anticipate working in maternity settings*,* it is nonetheless a requirement for completion of their program” (* [34], p. *1).*

#### Regulatory and accreditation determinations and guidelines

In total, 66 documents described regulatory and accreditation determinations and guidelines. These were grouped into four categories (as seen in Table 9) including: *Directives and recommendations from regulatory and accreditation bodies* (*n* = 36); *COVID-19 as a catalyst for placement flexibility*, (*n* = 16); *Lack of regulator and accreditor specification and direction for replacement of traditional clinical placements with SCPs* and *Evidence being sought by regulators*,* accreditors and universities*. There was acknowledgement in the included documents that regulatory agencies and accreditation bodies sometimes have no specifications or directions to guide the replacement of traditional clinical placement with SCPs (*n* = 15).

**Table 9:** Regulatory and accreditation determinations and guidelines

Category	Description	Example of extracted data	*n*
Directives and recommendations from regulatory and accreditation bodies	Information that provided a direction or recommendation by regulatory or accreditation bodies for the use of SCP.	In Australia, the professional accrediting body for occupational therapy currently allows up to 200 of the mandatory 1000 clinical placement hours to be completed via simulation activities [20].	36
COVID-19 as a catalyst for placement flexibility	Information where regulatory or accreditation bodies have identified that their practice needs to change either permanently or temporarily due to COVID-19.	During the pandemic, simulated placements were vital in helping students make up placement hours. The Nursing and Midwifery Council granted special dispensation to allow 300 of the required 2300 placement hours to be in a simulated environment. Universities and other education providers have subsequently been permitted to apply to increase this to 600. These changes have been made permanent as other special pandemic measures were allowed to lapse [18].	16
Lack of regulator and accreditor specification and direction for replacement of traditional clinical placement with SCPs	Information where the authors of the included documents identified that their regulatory or accreditation body has not provided any directives or recommendations and the potential consequences from this silence.	The Health and Care Professions Council and the College of Radiographers do not stipulate the minimum practice hours required in pre-registration programmes, instead focussing on appropriate curriculum and practice placements to support students to reach the required standards. In contrast, CORU, the regulatory body of diagnostic radiographers in Ireland require approved programmes to have 1200 h of practice placements [64].	15
Evidence being sought by regulators, accreditors and universities	Any information where regulators, or accreditors queried the current evidence surrounding SCPs, or the need for further research within this area prior to providing a directive of recommendation.	The National Council of State Boards of Nursing (US; NCSBN) has challenged state boards of nursing to develop specific guidelines regarding the use of simulation in prelicensure nursing programs. However, there is insufficient research on which to develop evidence-based practices to create new models of clinical education delivery that incorporate simulation as a component. In particular, because the NCSBN National Simulation Study did not study the influence of the sequence of clinical learning environments and simulated learning environments, this warrants further examination [23].	8

#### Resources used for simulated clinical placement

There were 93 documents that reported the type of resources that were used in SCPs. These were categorised into *capital*,* human*,* technological*, and *student resources*.

There was a plethora of *capital resources* (*n* = 132) identified in the included documents. These included manikins which were frequently used alongside SPs in scripted scenarios. Dedicated simulation facilities were frequently mentioned. These often-replicated clinical environments such as hospital wards, operating rooms, office and interview rooms, and rehabilitation spaces. Many simulation facilities had observation rooms adjacent to the simulation room (e.g. two-way mirror) or capacity to stream video recordings to a separate observation room. Simulation facilities were furnished with clinical equipment such as hospital beds, patient plinths, posters/signs (e.g. for handwashing), IV poles, and anaesthesia machines. Clinical artefacts, such as de-identified patient records (paper-based and electronic health records), X-rays, MRIs, and blood tests, patient charts, care plans, and audio clips, were also used in the simulations.

SCPs required extensive *human resources* (*n* = 118) to operate. Human resources included clinical educators and instructors, simulation coordinators (including program managers), health professionals, simulated patients/participants (in paid and voluntary capacities), and simulation technicians.

Documents reported various *technological resources* (*n* = 62) including software specific to health simulation (such as Simucase, vSim for Nursing, Shaderware, Digital Clinical Experience^®^, and Sentinel City^®^), learning management systems which supported student learning (Desire2Learn, Canvas, and Blackboard), and platforms which facilitated engagement and communication (Zoom, Microsoft Teams, WebEx, PollEverywhere, Flipgrid, and Google Forms).

Documents reported that students required resources to participate in SCPs, which were either at the student’s own cost or provided by the university. *Student resources* (*n* = 30) included personal technological devices (laptops, iPads, phones) with internet access and specified software. Students often had access to guides or pre-briefing documents, workbooks (which included expectations, case data, timelines, reflections etc.), case documents, reading materials (online or within textbooks), study questions, video recordings of simulated scenarios and quizzes. There were some documents that offered information sessions to alleviate concerns about the process of SCPs.

### Evaluation of SCPs

Eighty-two documents reported student outcomes. These were categorised into *Student performance outcomes*, *Student satisfaction and evaluation of SCPs* and *Student preparedness for practice*. Examples of study outcomes relating to each of these categories are reported in column two of Table 10. In column four, validated and recognised measurement tools that were used in one or more included documents are listed.

**Table 10:** Outcomes of SCP research

Student outcomes	Examples	*n*	Measurement tools
Student performance outcomes	- Student clinical performance - Student achievement of clinical education objectives and clinical performance - Student competency to practice as an entry-level physiotherapist in the cardiorespiratory field - Student performance - Student proficiency - Student reaction - Self-confidence - Self-efficacy - Self-study time	57	Acute Care Confidence Survey (ACCS); American Occupational Therapy Association Fieldwork Performance Evaluation: AMSER exam: Assessment of Physiotherapy Practice (APP) tool; ATI Content Mastery Series (CMS); ATI RN Comprehensive Predictor (2010); ATI RN Mental Health Mastery Examination 2013 ATI TEAS (Test of Essential Academic Skills) Basic Knowledge Assessment Tool-6; Critical Thinking Diagnostic; Creighton Competency Evaluation Instrument (CCEI**);** General Attitude Towards Clinical lO; Spielberger’s State-Trait Anxiety Inventory (State Score); General Self Efficacy Scale; Global Assessment of Clinical Competency and Readiness for Practice; Health Science Reasoning Test; Mental Health Nursing Clinical Confidence Scale (MHNCCS); National Board for Certification in Occupational Therapy (NBCOT) first time pass rate exam data
Student satisfaction and evaluation of SCP	- Quality of Simulated clinical placement - Satisfaction - Satisfaction level with sEHR, e-PBL, and online-VMI useful for learning - Student needs - Student perceptions of educational practices - Student perceptions of the simulation experience - Students’ attitudes - Student engagement	45	Clinical Experience/Relevance Scale; Clinical Learning Environment Comparison Survey; The Student Satisfaction with Learning Scale and the Self- Confidence in Learning Using Simulations Scale; measure of quality of giving feedback scale; Satisfaction with Simulated Experience Scale
Students’ preparedness for practice	- Prepared for entry-level nursing practice. - Preparedness in professional and clinical skills at the beginning and end of the students’ placements - Student clinical readiness	7	Clinical Experience/Relevance Scale Clinical Learning Environment Comparison Survey; the New Graduate Nurse Performance Survey (NGNPS)

#### Costs related to SCPs

There were 18 documents that provided data about direct and/or indirect costs related to SCPs. Direct costs include products (e.g. software, manikins, clinical environment set-up) or labour costs. The included documents identified set-up, capital, and personnel costs, cost per student and costs to student. Information about direct cost savings were also reported.

High initial set up costs were acknowledged by three documents [19, 20, 51]. On average, developing one scenario was costed at AUD$1700. Associated establishment costs included the purchasing of hospital equipment (IV poles, hospital beds, gowns, clinic signs, patient files), office equipment (chairs, tables, stationary), technology (iPads, health apps, simulation software, videos), and simulators (mannikins) [20, 35, 62].

In both Gospodarevskaya et al., [20] and Ward et al., and [62], human resources were the highest contributor to the cost of SCPs. Human resources included SPs, simulation clinical educators, simulation co-ordinators [62]. Gospodarevskaya et al., [20] identified that human resources cost AUD $370 per student during the simulation week. The average time staff spent preparing for a SCP added an additional AUD$344 per student. The cost of staff contributed to 76.6% of the cost per student for SCPs, and 59.71% when all costs were accounted. Fitzgerald et al., [19] estimated the cost of employing SPs for their SCPs was approximately AUD$300-$400 per case. This cost included time in the simulation, and their training.

Gospodarevskaya et al., [20] conducted an economic evaluation comparing SCPs to traditional clinical placements in occupational therapy education to complement an Australian randomised control trial conducted across six universities. In this study, the cost per student undertaking a SCPs ranged from AUD$460 to AUD$1511 compared to between AUD$144 to AUD$1112 for students undertaking a traditional clinical placement. Accounting for staff hours, and other direct and indirect costs, the mean average cost to the university per student, for a 40- hour simulation week, was AUD$893 across the six universities. The average cost per student on a traditional clinical placement was lower at AUD$677 across the six universities.

Ward et al., [62] examined the cost associated with delivering a 5-day SCP for speech–language pathology students from six Australian universities, however, did not compare these with traditional clinical placements. The cost of conducting the SCPs per programme across the universities ranged from AUD$4717 to AUD$11,425. The approximate cost per student ranged from AUD$683 to AUD$1087, with an average of AUD$859.

SCPs were reported, in some instances, to reduce the costs of attending placements for the student. Sadd’s [53] commentary suggests that nursing students have significant travel costs relating to clinical placements. This was supported by Villa et al., [61] who identified that a virtual clerkship for students at 22 institutions incurred no costs, whilst costs for students who undertook traditional placements, were approximately USD$1000 per rotation.

Gospodarevskaya et al., [20] reported that although initially costs associated with staff ratios may be high, once set up, SCPs can easily scale up, as the preparation time remains the same despite the number of students. For example, Fitzgerald et al., [19] reported that the staff ratio could be 1:10 for three simulated sessions costing the institution approximately AUD$9600 per semester, whilst traditional placement requires a 1:5 ratio, ten placement blocks for an additional three weeks costing the institution AUD$19,200 per semester. Therefore, SCPs can support high numbers of students that cannot be facilitated in traditional placements and the cost per student is reduced. Utilising existing materials or institutional property was also a cost-saving measure that some SCPs undertook. For example, the Korayem and Alboghdadly [37] study, which described a 5-week pharmacy SCP, acknowledged that using existing facilities at Princess Noura bint Abdulrahman University saved them 25,000 Saudi Riyals of the training costs per rotation.

There were four studies that advocated for virtual SCPs as a cost saving measure ([19]; [32, 40, 65]). These studies reported that virtual SCPs did not require room hire and potentially have less administrative burden. This cost saving measure is dependent on the methods used to deliver the virtual simulation. For example, it was reported that developing an asynchronous DVD simulation is a one-off investment, with very little associated costs for its continuing delivery [65]. Other cost saving measures included using open-source simulations [16] and staff and students sharing resources [30].

#### Barriers, facilitators, strengths, and weaknesses of SCP

**Fig. 5:**
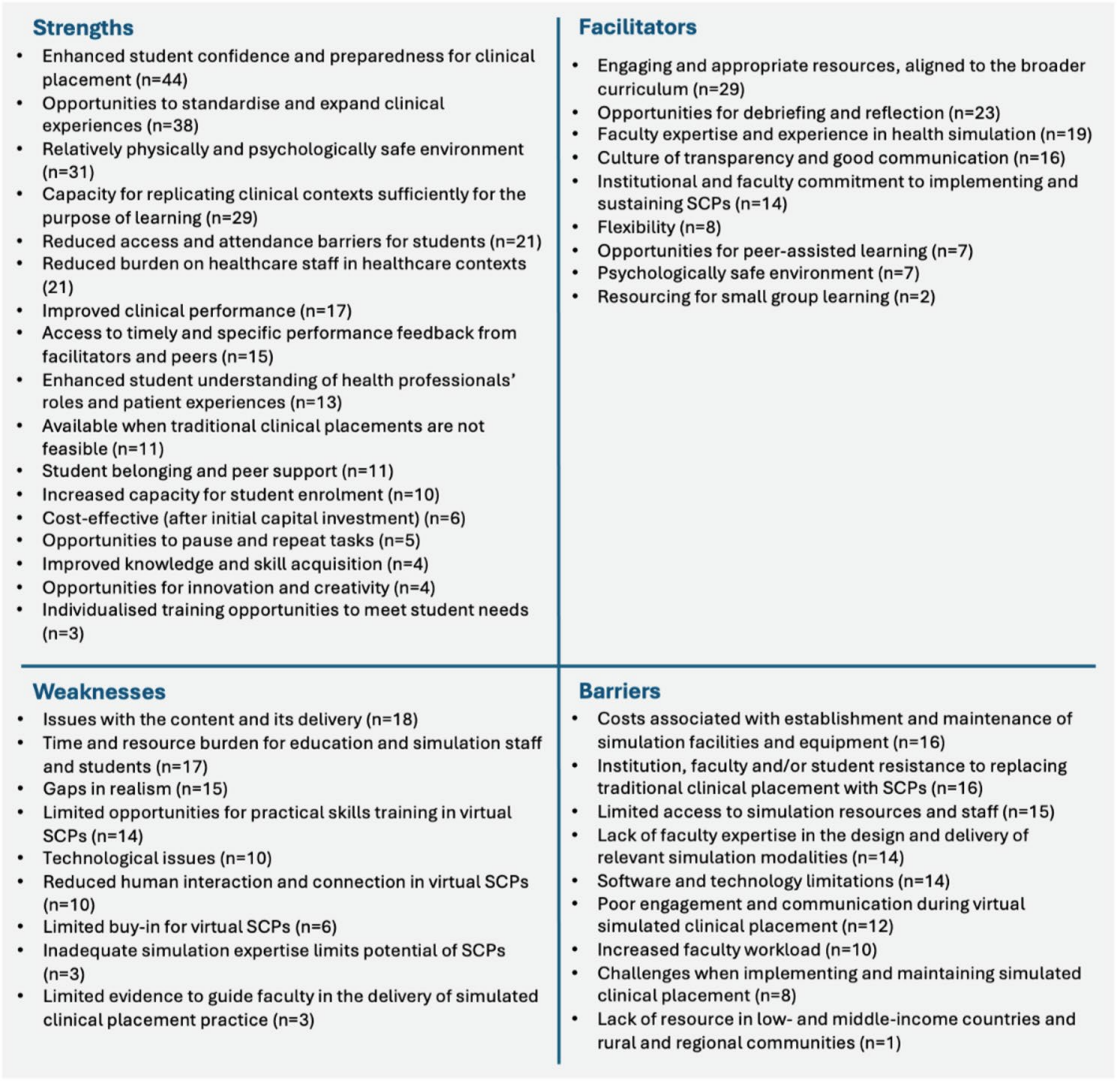
Strengths, weaknesses, facilitators, barriers and weaknesses of SCPs

There were substantial data collected regarding the strengths, weaknesses, facilitators, and barriers of SCPs (refer to Fig. 5). Ninety documents described strengths of SCPs and were grouped into 17 categories. The most frequently reported strengths of SCPs were *enhanced student confidence and preparedness for clinical placement* (*n* = 44), and *opportunities to standardise and expand clinical experiences* (*n* = 38). Fifty-three documents reported weaknesses of SCPs and were grouped into nine categories. The most frequently identified weaknesses were *issues with content and delivery* (*n* = 18), and *time and resource burden for education and simulation staff and students* (*n* = 17). Facilitators of SCPs were identified in 68 documents and organised into nine categories. The most frequently reported facilitators of SCPs were *engaging and appropriate resources aligned to the curriculum* (*n* = 29), and *opportunities for debriefing and reflection* (*n* = 23). There were 68 documents which discussed barriers to SCPs, and these were organised into nine categories. The most frequent barriers to SCPs included the *costs associated with the establishment and maintenance of simulation facilities and equipment* (*n* = 16), and *institution*,* faculty or student resistance to SCPs* (*n* = 16).

## Discussion

This scoping review sought to identify and synthesise evidence relating to SCPs that have been used to replace traditional clinical placements. Specifically, the review aimed to map the evidence relating to the design, delivery, implementation and evaluation of SCPs, with consideration to how they are integrated into the broader curricula. In this scoping review, 131 documents met eligibility criteria, including original research documents, reviews, discussion papers and editorials. Data relating to multiple facets of the design, delivery, implementation and evaluation of SCPs were extracted to address the review questions. Synthesised data indicate that SCPs continue to be conducted in a variety of ways to meet a variety of contextual needs, employing a variety of methods, simulation modalities and resources [52].

### Conceptualisation, design and delivery of SCPs

In their meta-narrative review, Roberts et al., [52] found that definitions for traditional and SCPs were unclear and inconsistent. Clarity does not appear to have improved in the subsequent five years, with no consistent definition of SCPs emerging from this review, and very few documents explicitly defining SCPs at all. Developing a standardised definition of SCPs, informed by clear theoretical and pedagogical principles, would improve research comparability and regulatory coherence. Further, an agreed-upon definition would support the implementation of SCPs that have clear learning objectives and structured competency frameworks, rather than as ad hoc solutions to placement shortages.

Alongside definition clarity, we sought to examine whether, and how, theoretical and conceptual frameworks have been used to support implementation, design and delivery of SCPs. A range of theoretical and conceptual frameworks to support the design and delivery of SCPs were described in the documents reviewed. Not unexpectedly, the most frequently cited theories fell into the categories of experiential learning and social constructivism, which are theories often linked to other health simulation activities [17, 49, 68].

Many of the reviewed documents did not mention a theoretical or conceptual framework, which may indicate a lack of explicit alignment between SCP design and learning theory. Given the importance of pedagogical models for influencing, for example, skill acquisition, clinical decision-making, and professional socialisation, this raises questions about whether design and implementation of SCPs need to be built around specific theories and frameworks. Future research needs to investigate whether or how those theories/frameworks inform SCPs and promote the development of key skills such as clinical reasoning, interprofessional competencies, and real-world problem-solving skills.

The ways in which SCPs have been delivered are incredibly diverse, with in-person, online and hybrid models deploying a range of simulation modalities, encompassing simulated patients, manikins and part-task trainers, computer-based simulations, virtual reality simulations and peer role play. Given the sheer range of options available, we pause to give due credit to the many hundreds of simulation educators who develop and use their expertise to optimise student experience and learner outcomes. We also pause to advocate for the recognition of expertise required to design and deliver all simulation activities. We note that in some institutions it was reported that staff were not afforded the time required to pivot from traditional to SCPs. If SCPs are to continue to be embedded in programs, institutional investment in sufficient human resources to establish and sustain these programs is required.

### Implementation and evaluation of SCPs

What is immediately evident in this review is that SCPs are increasingly being established globally and across multiple health professions programs in efforts to meet learner and institutional needs [39]. Implementation of SCPs appears to have accelerated significantly in response to reduced access to clinical environments and ‘placement hours’ during the COVID-19 pandemic – three quarters of included documents in this review mention the pandemic. While limitations in the number of available traditional clinical placements were already a factor for SCP adoption [39, 41, 52], the unanticipated and unprecedented impact of the pandemic catalysed their evolution, creating opportunity to address longstanding placement challenges while preserving educational outcomes.

As we emerge from the Covid-19 pandemic years, we have opportunities to learn from the adaptations that were necessitated by the pandemic. SCPs are now positioned not only as emergency replacements for traditional placements, but as a long-term solution to placement shortages, education equity, and standardised skill acquisition. With a better understanding of the benefits and detractions of different ways of preparing health professions students for clinical practice, decisions can be made to enhance clinical placement models and the sustainability of health professions education.

The quality of SCPs and its measures were not a focus in this review, but there is an acknowledged need for further investigation of SCP quality and methods for measuring this quality [56]. As recommended by previous systematic reviews on this topic, standardised simulation curricula, coupled with consistent evaluative methods would be helpful for measuring the quality, effectiveness and impact of SCPs on student learning, student performance and student readiness for clinical practice [4, 39]. For example, establishing a core outcome set to standardised evaluation would be invaluable for synthesising evidence to guide decision-making and resource allocation. Further to this, determining the characteristics of high-quality SCPs, as recommended by Smith et al., [56], would be beneficial for benchmarking between and within placement programs in different professions and different institutions. In addition to standardised evaluation tools and benchmarking considerations, guidance on how to report the cost and return on investment of SCPs would be invaluable to institutions wanting to establish or optimise SCPs.

### Strategic role and future directions

The extent to which SCPs should replace traditional placements remains an open question. Findings suggest that while SCPs offer unique advantages in controlling learning experiences, ensuring standardised competency development, and providing relatively safe environments for skill acquisition, their ability to develop professional adaptability, patient interaction skills, and interprofessional collaboration requires further investigation.

The potential for SCPs to enhance, rather than simply replace traditional placements, should be a key focus of future research. Decisions about SCPs require more than just addressing the question, ‘how many hours of traditional clinical placement can be replaced with simulation?’ A more sophisticated approach is needed: one that considers evidence about the design, delivery, implementation, and evaluation of SCPs is required. This deeper consideration must also include integration into the broader curriculum and articulation with other experiential learning opportunities.

SCPs represent a significant shift in clinical education models, offering potential solutions to placement shortages, inconsistencies in learning opportunities, and evolving regulatory requirements. However, their long-term role in workforce readiness and professional competency development remains underexplored. Regulatory frameworks must be clarified to ensure consistency in SCP accreditation and integration into health professions curricula.

We believe that the successful future of SCPs hinges on careful alignment of curriculum goals, learner development, and educational standards. SCPs should not be viewed merely as stopgaps or temporary responses to placement shortages, but as opportunities to innovate and enhance clinical education. Their potential lies in structured, competency-driven experiences that are underpinned by strong pedagogical principles, expert simulation educators and institutional support.

Jeffries, [29] captures this imperative when stating that “the type of simulation used needs to take into account the level of the student, the concepts being taught, and the theoretical knowledge required” (p 100). They further stress the necessity for regulation, noting that simulation must be used in ways that ensure consistent quality across institutions to produce graduates who are ready for clinical practice. This underscores the urgency for clearer national standards, shared definitions, and robust quality assurance mechanisms that protect the integrity and outcomes of SCPs.

### Limitations

This review provides a comprehensive examination of SCPs and was conducted in a transparent and rigorous manner consistent with the conduct of scoping reviews. However, as there is no definition of SCPs, we took a broad approach to inclusion which may have led to subjective interpretation by our team. To mitigate this issue, we ensured that each document was reviewed by two reviewers who were able to consult with content experts on the authorship team to come to a conclusion regarding their eligibility.

An additional limitation of this review is its breadth. As we were interested in mapping the landscape of the literature relating to SCPs, our net was cast widely, and we included studies and articles that do not allow for easy comparison. Our ability, and that of the readers, to draw conclusions about what approach to developing SCPs are most appropriate is constrained by the heterogeneity of included articles, as well as by the methodological choices underpinning this review, – i.e. a scoping review. A systematic review of articles that meet narrower criteria may support some direct comparison between SCP programs, rather than the narrative overview that is presented here.

### Concluding statement

Universities, healthcare services and accrediting bodies are exploring how to use simulation effectively to ensure students are graduating with sufficient clinical experience and skills. SCPs are increasingly integrated into health professions education programs to meet learner needs and requirements for clinical experience. This review has mapped the current state of literature that has described and discussed SCPs across various health professions. The design, delivery, implementation and evaluation of SCPs across the health landscape is variable. Bespoke SCP programs have been developed for a broad range of pragmatic and educational reasons, comprehensive of limited capacity in health services to accommodate students, cost considerations of SCPs vs. traditional clinical placements, and the predictability of clinical skill attainment and case exposure afforded by SCPs. The length, expectations, simulation modalities, settings and outcome measures of SCPs varies considerably, with little opportunity to draw direct comparison between delivery and outcomes.

SCPs have the capacity to transform clinical education. They offer important approaches and affordances for achieving excellence and equity in health professions education, while also addressing the systemic pressures of placement provision and workforce expansion. It seems likely that SCPs will continue to be established. As they are established, we advocate for continued consideration of the theoretical and conceptual underpinnings of SCPs; for appropriate alignment between learning outcomes and the modalities of simulation that are selected; and for investment in the human and capital resources to support SCPs.

## Supplementary Information

Supplementary Material 1.

Supplementary Material 2.

## Data Availability

The datasets generated and/or analysed during the current study are available in the projects Open Science Framework: https://osf.io/q39am/.
