# Supplementary material for: Design, delivery, implementation, and evaluation of simulated clinical placements: a scoping review and evidence and gap map of the current state of evidence
Source: Adv Simul (Lond). 2026 Feb 18;11:31. doi: 10.1186/s41077-026-00420-3 (PMC13067688; doi:10.1186/s41077-026-00420-3)
Supplement: Supplementary file 1 — Supplementary Material 1. [file 41077_2026_420_MOESM1_ESM.pdf]

Simulated clinical placements scoping review - supplementary file

Contents

Simulated clinical placements scoping review - supplementary file 1
Data extraction file 2
PRISMA-ScR 2
Deviations from Protocol 3
Data dictionary 3
Database search strategy and results 6
Right-hand search through academic journal results 11
Supplemental search 13
List of reviews 13
Studies screened after duplicates removed 14
Contacting authors 24
List of excluded studies 24
Queried articles 33
All included studies 34
Data extraction instrument 38
References 40

Data extraction file

The data extraction file can be located on the Open Science Framework website: https://osf.io/q39am/.

PRISMA-ScR

SECTION	ITEM	PRISMA-ScR CHECKLIST ITEM	REPORTED ON PAGE #
**TITLE**			
Title	1	Identify the report as a scoping review.	1
**ABSTRACT**			
Structured summary	2	Provide a structured summary that includes (as applicable): background, objectives, eligibility criteria, sources of evidence, charting methods, results, and conclusions that relate to the review questions and objectives.	1
**INTRODUCTION**			
Rationale	3	Describe the rationale for the review in the context of what is already known. Explain why the review questions/objectives lend themselves to a scoping review approach.	3
Objectives	4	Provide an explicit statement of the questions and objectives being addressed with reference to their key elements (e.g., population or participants, concepts, and context) or other relevant key elements used to conceptualize the review questions and/or objectives.	3
**METHODS**			
Protocol and registration	5	Indicate whether a review protocol exists; state if and where it can be accessed (e.g., a Web address); and if available, provide registration information, including the registration number.	3
Eligibility criteria	6	Specify characteristics of the sources of evidence used as eligibility criteria (e.g., years considered, language, and publication status), and provide a rationale.	4
Information sources*	7	Describe all information sources in the search (e.g., databases with dates of coverage and contact with authors to identify additional sources), as well as the date the most recent search was executed.	5
Search	8	Present the full electronic search strategy for at least 1 database, including any limits used, such that it could be repeated.	Supplementary materials pages 7-11
Selection of sources of evidence†	9	State the process for selecting sources of evidence (i.e., screening and eligibility) included in the scoping review.	5-6
Data charting process‡	10	Describe the methods of charting data from the included sources of evidence (e.g., calibrated forms or forms that have been tested by the team before their use, and whether data charting was done independently or in duplicate) and any processes for obtaining and confirming data from investigators.	6
Data items	11	List and define all variables for which data were sought and any assumptions and simplifications made.	Supplementary Materials pages 55-58
Critical appraisal of individual sources of evidence§	12	If done, provide a rationale for conducting a critical appraisal of included sources of evidence; describe the methods used and how this information was used in any data synthesis (if appropriate).	NA
Synthesis of results	13	Describe the methods of handling and summarizing the data that were charted.	6
**RESULTS**			
Selection of sources of evidence	14	Give numbers of sources of evidence screened, assessed for eligibility, and included in the review, with reasons for exclusions at each stage, ideally using a flow diagram.	7
Characteristics of sources of evidence	15	For each source of evidence, present characteristics for which data were charted and provide the citations.	8
Critical appraisal within sources of evidence	16	If done, present data on critical appraisal of included sources of evidence (see item 12).	NA
Results of individual sources of evidence	17	For each included source of evidence, present the relevant data that were charted that relate to the review questions and objectives.	Uploaded to OSF
Synthesis of results	18	Summarize and/or present the charting results as they relate to the review questions and objectives.	9-20
**DISCUSSION**			
Summary of evidence	19	Summarize the main results (including an overview of concepts, themes, and types of evidence available), link to the review	20-23

SECTION	ITEM	PRISMA-ScR CHECKLIST ITEM	REPORTED ON PAGE #
		questions and objectives, and consider the relevance to key groups.	
Limitations	20	Discuss the limitations of the scoping review process.	23
Conclusions	21	Provide a general interpretation of the results with respect to the review questions and objectives, as well as potential implications and/or next steps.	23
**FUNDING**			
Funding	22	Describe sources of funding for the included sources of evidence, as well as sources of funding for the scoping review. Describe the role of the funders of the scoping review.	Listed in declarations under Funding

JBI = Joanna Briggs Institute; PRISMA-ScR = Preferred Reporting Items for Systematic reviews and Meta-Analyses extension for Scoping Reviews.
* Where *sources of evidence* (see second footnote) are compiled from, such as bibliographic databases, social media platforms, and Web sites.
† A more inclusive/heterogeneous term used to account for the different types of evidence or data sources (e.g., quantitative and/or qualitative research, expert opinion, and policy documents) that may be eligible in a scoping review as opposed to only studies. This is not to be confused with *information sources* (see first footnote).
‡ The frameworks by Arksey and O'Malley (6) and Levac and colleagues (7) and the JBI guidance (4, 5) refer to the process of data extraction in a scoping review as data charting.
§ The process of systematically examining research evidence to assess its validity, results, and relevance before using it to inform a decision. This term is used for items 12 and 19 instead of "risk of bias" (which is more applicable to systematic reviews of interventions) to include and acknowledge the various sources of evidence that may be used in a scoping review (e.g., quantitative and/or qualitative research, expert opinion, and policy document).*From:* Tricco AC, Lillie E, Zarin W, O'Brien KK, Colquhoun H, Levac D, et al. PRISMA Extension for Scoping Reviews (PRISMAScR): Checklist and Explanation. Ann Intern Med. 2018;169:467–473. doi: 10.7326/M18-0850.

Deviations from Protocol

The following deviations from the protocol occurred during the conduct of this scoping review.

- Forward and backward citation searching of the included sources was NOT conducted. Examination of the reference list included reviews did occur.

Data dictionary

Term	Definition
**Definitions and extraction expectations specific for the concept and context**	
**Health professional**	This review will adopt the definition of *health professional* developed by the International Standard Classification of Occupations, 2008 revision (ISCO-08) [1] The review will include the ISCO-08 minor groups of health professionals shown below. These are classified under the sub-major group 22 (health professionals), which is part of the major group 2 (professionals).<br>ISCO-08 minor groups of health professionals included in this review:<br>• 221– Medical doctors<br>• 222 – Nursing and midwifery professionals<br>• 223 – Traditional and complementary medicine professionals<br>• 224 – Paramedical practitioners<br>• 225 – Veterinarians<br>• 226 – Other health professionals
**Simulated Clinical Placement (SCP)**	This review will only include sources that self-identify as relating specifically to SCPs. This will include SCPs used as a sole replacement for traditional clinical placements, or if SCPs have been used in combination with traditional clinical placements. Both in-person and virtual SCPs, or a mixture of both modalities, will be included. For this review, SCPs must be practical placements, – i.e. discrete tasks that use simulation that are not a component of a SCP, and presentations about SCPs (e.g. SCP-related coursework) will be excluded.
**Traditional clinical placement (in-person)**	"A clinical placement can be defined as any arrangement where a student is present, for educational purposes, in an environment that provides healthcare or related services to patients or the public. Placements can take place in primary, secondary, community or other health and social care settings (with some aspects being undertaken remotely such as consultations) and are not confined to the working environment of doctors but should include the wider multidisciplinary team. Students can be actively involved and contribute to patient care as part of the team." [2]
**Simulation-based learning education**	"An array of structured activities that represent actual or potential situations in education and practice. These activities allow participants to develop or enhance their knowledge, skills, and attitudes, or to analyze and respond to realistic situations in a simulated environment" [3].

**Simulation modalities**	"A term used to refer to the type(s) of simulation being used as part of the simulation activity, for example, task trainers, manikin based, standardized/simulated patients, computer based, virtual reality, and hybrid."[4]
**Methods of the SCP**	How the SCP was developed and conducted.<br>In this review, the description of how the SCP was developed and conducted will be extracted.
**Resources used during the SCP**	Describes the labour and capital (equipment, tools) required to complete the SCP.<br>Examples of labour resources: the training required of trainers, the hours trainers need to prepare and conduct the SCP.<br>Examples of capital resources: the type of mannequins, software, location used, health and medical equipment.<br>In this review, information on resources used during the SCP will be extracted.
**Methods of simulations used in the SCP**	What specific simulation-based education activities were components of the SCP. This could include role-playing with a person/actor or mannequin, use of mannequins or phantoms, or virtual reality.<br>In this review the name and description of the specific simulation-based education activities that were components of the SCP will be extracted.
**Outcomes used in the evaluation of the SCP**	"A result of an activity the learners demonstrate by the end of an educational activity in terms of knowledge, skills, and attributes (KSAs) acquired." (pg 27 [5]))..<br>In this review, each type of outcome used in the evaluation of the SCP will be extracted.
**Outcome measures used for the evaluation of the SCP**	The scales or tools that measured the outcome used for the evaluation of the SCP. For example, the SICKS scale measures students' confidence.<br>For this review, the name of any specific scales or tools for specified outcome measures will be extracted.
**Theoretical underpinnings**	Theoretical underpinnings can also be described as learning theories and are the "lens in which to understand how learning occurs (e.g. cognitive, constructivist, sociocultural perspectives)" [6].<br>In this review, the name of the theoretical underpinnings of the SCP will be extracted.
**Conceptual frameworks**	Conceptual frameworks are "ways of thinking about a problem or a study, or ways of representing how complex things work the way they do" (p. 313)[7].<br>In this review:<br>- Only conceptual frameworks that specifically relate or have been applied to SCPs will be extracted.<br>- The name and description of the conceptual framework will also be extracted.
**Learning objectives**	"Expected goal of a curriculum, course, lesson or activity in terms of demonstrable skills or knowledge that will be acquired by a student as a result of instruction" pg 27[5].<br>In this review, all learning objectives of the SCP listed within the source will be extracted.
**How a student is assessed within the SCP**	In this review, a list and description of the formative and summative assessments with which a student has been evaluated within the SCP will be extracted.
**Hours allocated for the SCP**	The duration in hours of the SCP for the student.<br>For this review, the number of hours the student will need to complete to pass the SCP will be extracted.
**Costs of the SCP**<br>- **Direct costs**<br>- **Indirect costs**	Costs can be direct or indirect.<br>Direct costs are costs that are directly associated with the SCP. These include products (e.g. software, mannequins, clinical environment set-up) or labor costs.<br>Indirect costs are those not directly associated within the SCP but that still need to be considered (e.g. administrative, utilities).<br>In this review, the name and expenditure of each direct and indirect cost will be extracted.
**Major findings of the study**	In this review, the findings that have been discussed within the abstract of each source will be extracted.
**Definition of the SCP**	Information in each source that specifically attempts to define what is a SCP will be extracted for this review.
**Regulatory, legislative, accreditation or qualification implications**	In this review, all information that discusses any regulatory, legislative, accreditation or qualification implications of SCPs will be extracted.

**Whether the SCP meets the accreditation needs for the particular health profession**	This will be a yes/no/not mentioned response for this review.<br><br>This refers to whether the evidence source has explicitly stated that students undertaking the SCP will meet the requirements for registration set by their health profession's accreditation body.
**Whether an evaluation of the SCP has taken place**	This will be a yes/no/planned/not mentioned response for this review.<br>This refers to whether the evidence source has explicitly stated that an evaluation of the SCP has taken place.
**Description of the evaluation of the SCP**	A description of how the value, nature, character, or quality of the SCP was determined. An evaluation may be formal or informal.<br><br>For this review, the extraction of the description of the evaluation will include information on how it occurred and what it evaluated.
**Integration of SCP within broader educational programs and practical ecosystems**	Whether the SCP is discussed or implied as being the entire educational process or a part of the wider curriculum. Whether the wider curriculum in which the SCP takes place is described within the discussion of the SCP. Whether or not the SCP is presented as part of a wider process of learning and teaching.<br>In this review, information on the integration of the SCP within broader educational programs and practical ecosystems, will be extracted.
**Strengths of the SCP**	The advantages of the SCP. Such as, patient-safety and diverse learning experiences.<br>In this review, information on either strengths explicitly stated by the author or aspects perceived as strengths by the reviewer, will be extracted.
**Weaknesses of the SCP**	The disadvantages of the SCP.<br>In this review, information on either weaknesses explicitly stated by the author or aspects perceived as weaknesses by the reviewer, will be extracted.
**Facilitators of the SCP**	A factor that makes the process of the SCP easy or easier. This could include software, administrative processes, attitudes, or knowledge. This does not include the specific role of a 'facilitator' who may conduct simulation-based education, unless the source specifically states that the facilitator made the process of the SCP easy or easier.<br>In this review, information on either factors that facilitate the process of the SCP that are explicitly stated by the author, or factors perceived by the reviewer to facilitate the process of the SCP, will be extracted.
**Barriers to the SCP**	A factor that makes the process of the SCP difficult or more difficult.<br>In this review, information on either barriers explicitly stated by the author or factors perceived as barriers by the reviewer, will be extracted.
**Study methods**	
**Experimental studies**	These include randomized controlled trials and their derivatives (e.g. cluster-randomized controlled trials, cross-over trials), pseudo-randomized controlled trials and quasi-experimental studies.
**Observational studies**	These include prospective and retrospective cohort studies, case-control studies, analytical cross-sectional studies and case series or case reports [8].
**Utility/health status value studies**	These include the following types of studies: standard gamble, time trade off, discrete choice, visual analogue scale, multi-attribute instruments, utility or health status values transformed from quality-of-life measurements [9].
**Close-ended questionnaires/surveys**	Surveys/questionnaires in which questions are presented that have an explicit/discrete/guided answer that is to be selected by the participants [10].
**Open-ended questionnaire/surveys**	Surveys/questionnaires in which questions are presented with no explicit/discrete/guided answer associated, and allow the participant to reflect personally on the question being asked [10].
**Focus groups**	Group interviews that capitalize on communication between research participants and the researcher to generate data [11].
**Interviews**	Semi-structured or unstructured communication between a single research participant and the researcher to generate data [12].
**Qualitative primary research**	Studies conducted to understand people's beliefs, experiences, attitudes, behaviors, and interactions. These include a range of methodologies and methods, for example phenomenology, action research and descriptive approaches.
**Mixed methods primary research**	Studies conducted using at least one qualitative and at least one quantitative method to answer a research problem.
**Discussion article**	Discussion articles present information from the perspective of the author(s) on a certain topic. They do not present information which has been collected through primary or secondary research methods.

**Literature review**	Traditional literature reviews are useful for describing an issue and its underlying concepts and theories. However, they rely heavily on the author's knowledge and experience and provide a limited presentation of a topic. Such reviews are often based on references chosen selectively from the evidence available, resulting in a review inherently at risk for bias or systematic error [13].
**Systematic review**	Systematic reviews seek to collate evidence that fits pre-specified eligibility criteria to answer a specific research question. They aim to minimize bias by using explicit, systematic methods documented in advance with a protocol [14].
**Scoping review**	Scoping reviews may be conducted where the purpose of the review is to identify knowledge gaps, scope a body of literature, clarify concepts or to investigate research conduct. While useful, scoping reviews may also be helpful precursors to systematic reviews and can be used to confirm the relevance of inclusion criteria and potential questions [15].

Database search strategy and results

**Topic**	The evidence for simulated clinical placements in health: A scoping review and evidence and gap map protocol
**Databases**	PubMed (NCBI), Embase (Elsevier), CINAHL Plus with Full Text (EBSCO), PsycINFO (EBSCO)
**Date Run**	May 2, 2024
**Results**	PubMed (NCBI): 11,980<br>Embase (Elsevier): 19,004<br>CINAHL Plus with Full Text (EBSCO): 9,120<br>PsycINFO: 3,333<br>TOTAL: 43,437
**Search Created By**	Carrie Price, MLS, Albert S. Cook Library, Towson University, Towson, Maryland, USA
**Search Peer Reviewed By**	John Reynolds, MLIS, Louise Calder Memorial Library, University of Miami, Miami, Florida, USA

**PubMed (NCBI)**		
**1**	("augmented reality"[majr] OR "simulation training"[majr] OR "high fidelity simulation training"[majr] OR "patient simulation"[majr] OR "virtual reality"[majr] OR "augmented realit*"[tiab] OR "simulation"[tiab] OR "simulations"[tiab] OR "simulate"[tiab] OR "simulated"[tiab] OR "SBE"[tiab] OR "HFST"[tiab] OR "mixed realit*" [tiab] OR "virtual placement*"[tiab] OR "virtual environment*"[tiab] OR "virtual realit*"[tiab])	715,170
**2**	("academia"[mesh:noexp] OR "curriculum"[mesh:noexp] OR "education"[mesh:noexp] OR "education"[sh] OR "education, graduate"[mesh:noexp] OR "education, professional"[mesh:noexp] OR "interdisciplinary studies"[mesh:noexp] OR "learning"[mesh:noexp] OR "teaching"[mesh:noexp] OR "academ*"[tiab] OR "curric*" [tiab] OR "education"[tiab] OR "learning"[tiab] OR "learner*"[tiab] OR "interdisciplinary studies"[tiab] OR "interprofessional"[tiab] OR "students"[mesh:noexp] OR "students"[tiab] OR "teaching"[tiab])	1,810,398
**3**	("Interdisciplinary Placement"[Mesh] OR "Clinical Competence"[Mesh] OR "placement"[tiab] OR "placements"[tiab] OR "program"[tiab] OR "programs"[tiab] OR "programme"[tiab] OR "programmes"[tiab] OR (("clinical"[tiab] OR "core"[tiab]) AND ("skill"[tiab] OR "skills*"[tiab] OR "competen*"[tiab] OR "expertise*"[tiab])))	1,327,335
**4**	("allied health personnel"[mesh:noexp] OR "allergists"[mesh:noexp] OR "anatomists"[mesh:noexp] OR "anesthesiologists"[mesh:noexp] OR "anesthetists"[mesh:noexp] OR "animal technicians"[mesh:noexp] OR "audiologists"[mesh:noexp] OR "cardiologists"[mesh:noexp] OR "caregivers"[mesh:noexp] OR "case managers"[mesh:noexp] OR "community health workers"[mesh:noexp] OR "coroners and medical examiners"[mesh:noexp] OR "dental auxiliaries"[mesh:noexp] OR "dental auxiliaries"[mesh:noexp] OR "dental staff"[mesh:noexp] OR "dental staff,	2,875,499

	hospital"[mesh:noexp] OR "dental staff, hospital"[mesh:noexp] OR "dentists"[mesh:noexp] OR "dermatologists"[mesh:noexp] OR "doulas"[mesh:noexp] OR "education, nursing, baccalaureate"[mesh:noexp] OR "education, pharmacy"[mesh:noexp] OR "emergency medical dispatcher"[mesh:noexp] OR "emergency medical technicians"[mesh:noexp] OR "endocrinologists"[mesh:noexp] OR "epidemiologists"[mesh:noexp] OR "faculty, dental"[mesh:noexp] OR "faculty, medical"[mesh:noexp] OR "faculty, nursing"[mesh:noexp] OR "foreign medical graduates"[mesh:noexp] OR "gastroenterologists"[mesh:noexp] OR "general practitioners"[mesh:noexp] OR "geriatricians"[mesh:noexp] OR "gynecologists"[mesh:noexp] OR "health educators"[mesh:noexp] OR "health facility administrators"[mesh:noexp] OR "health personnel"[mesh] OR "hospital administrators"[mesh:noexp] OR "hospital volunteers"[mesh:noexp] OR "hospitalists"[mesh:noexp] OR "infection control practitioners"[mesh:noexp] OR "licensed practical nurses"[mesh:noexp] OR "medical chaperones"[mesh:noexp] OR "medical laboratory personnel"[mesh:noexp] OR "medical record administrators"[mesh:noexp] OR "medical secretaries"[mesh:noexp] OR "medical staff"[mesh:noexp] OR "medical staff, hospital"[mesh:noexp] OR "midwifery"[mesh:noexp] OR "nephrologists"[mesh:noexp] OR "neurologists"[mesh:noexp] OR "nurse administrators"[mesh:noexp] OR "nurse anesthetists"[mesh:noexp] OR "nurse practitioners"[mesh:noexp] OR "nurse specialists"[mesh:noexp] OR "nurses"[mesh:noexp] OR "nurses, community health"[mesh:noexp] OR "nurses, international"[mesh:noexp] OR "nurses, male"[mesh:noexp] OR "nurses, public health"[mesh:noexp] OR "nursing assistants"[mesh:noexp] OR "nursing staff"[mesh:noexp] OR "nursing staff, hospital"[mesh:noexp] OR "nursing staff, hospital"[mesh:noexp] OR "nutritionists"[mesh:noexp] OR "obstetricians"[mesh:noexp] OR "occupational health physicians"[mesh:noexp] OR "occupational therapists"[mesh] OR "oncologists"[mesh:noexp] OR "operating room technicians"[mesh:noexp] OR "ophthalmologists"[mesh:noexp] OR "optometrists"[mesh:noexp] OR "osteopathic physicians"[mesh:noexp] OR "otolaryngologists"[mesh:noexp] OR "paramedics"[mesh:noexp] OR "pathologists"[mesh:noexp] OR "pediatricians"[mesh:noexp] OR "personnel, hospital"[mesh:noexp] OR "pharmacists"[mesh:noexp] OR "pharmacy technicians"[mesh:noexp] OR "physiatrists"[mesh:noexp] OR "physical therapist assistants"[mesh:noexp] OR "physical therapists"[mesh:noexp] OR "physician executives"[mesh:noexp] OR "physician assistants"[mesh:noexp] OR "physicians"[mesh:noexp] OR "physicians, family"[mesh:noexp] OR "physicians, primary care"[mesh:noexp] OR "physicians, women"[mesh:noexp] OR "psychiatrists"[mesh:noexp] OR "psychotherapists"[mesh:noexp] OR "pulmonologists"[mesh:noexp] OR "radiologists"[mesh:noexp] OR "rheumatologists"[mesh:noexp] OR "surgeons"[mesh:noexp] OR "traditional medicine practitioners"[mesh:noexp] OR "urologists"[mesh:noexp] OR "veterinarians"[mesh:noexp] OR "acupuncturist*"[tiab] OR "allergist*"[tiab] OR "allied health"[tiab] OR "anatomist*"[tiab] OR "anesthesiologist*"[tiab] OR "anesthetist*"[tiab] OR "animal technician*"[tiab] OR "arts therapist*"[tiab] OR "audiologist*"[tiab] OR "cardiologists"[tiab] OR "caregiver*"[tiab] OR "case manager*"[tiab] OR "chiropractor*"[tiab] OR "community health worker*"[tiab] OR "coroner*"[tiab] OR "dental auxiliar*"[tiab] OR "dental staff"[tiab] OR "dental assistant*"[tiab] OR "dental technician*"[tiab] OR "dental hygienist*"[tiab] OR "dentist*"[tiab] OR "dermatologist*"[tiab] OR "dietician*"[tiab] OR "dietitian*"[tiab] OR "dietetic*"[tiab] OR "doctor*"[tiab] OR "doula*"[tiab] OR "emergency medical dispatcher*"[tiab] OR "emergency medical technician*"[tiab] OR "endocrinologist*"[tiab] OR "epidemiologist*"[tiab] OR "gastroenterologists"[tiab] OR "general practitioner*"[tiab] OR "geriatrician*"[tiab] OR "gerontologist*"[tiab] OR "gynecologist*"[tiab] OR "health educator*"[tiab] OR "health facility administrator*"[tiab] OR "health personnel"[tiab] OR "health profession*"[tiab] OR "hospital administrator*"[tiab] OR "hospitalist*"[tiab] OR "medical chaperone*"[tiab] OR "medical examiner*"[tiab] OR "medical laboratory personnel"[tiab] OR "medical record administrator*"[tiab] OR "medical secretar*"[tiab] OR "medical staff"[tiab] OR "midwife*"[tiab] OR "midwifery"[tiab] OR "midwive*"[tiab] OR "naturopath*"[tiab] OR "nephrologist*"[tiab] OR "neurologist*"[tiab] OR "nurse*"[tiab] OR "nursing"[tiab] OR "nutritionist*"[tiab] OR "obstetrician*"[tiab] OR "occupational therapist*"[tiab] OR "occupational therapy"[tiab] OR "oncologist*"[tiab] OR "operating room technician*"[tiab] OR "ophthalmologist*"[tiab] OR "optometrist*"[tiab] OR "optician*"[tiab] OR<br>"osteopath*"[tiab] OR "otolaryngologist*"[tiab] OR "paramedic*"[tiab] OR "pathologist*"[tiab] OR "pediatrician*"[tiab] OR "pharmacist*"[tiab] OR "pharmacy technician*"[tiab] OR "pharmacy"[tiab] OR "physiatr*"[tiab] OR "physical therapist*"[tiab] OR "physical therapy"[tiab] OR "physician*"[tiab] OR "physiotherap*"[tiab] OR "podiatrist*"[tiab] OR "practitioner*"[tiab] OR "psychiatrist*"[tiab] OR "psychotherapist*"[tiab] OR "psychologist*"[tiab] OR "therapist*"[tiab] OR "counsellor*"[tiab] OR "counselor*"[tiab] OR "pulmonologist*"[tiab] OR "radiation technician*"[tiab] OR "radiolog*"[tiab] OR	

	"rehabilitation"[tiab] OR "rheumatologist*"[tiab] OR "social worker*"[tiab] OR "speech language"[tiab] OR "speech therapist*"[tiab] OR "surgeon*"[tiab] OR "urologist*"[tiab] OR "veterinarian*"[tiab])	
**5**	#1 AND #2 AND #3 AND #4	11,980

**Embase (Elsevier)**		
**1**	('augmented reality'/exp/mj OR 'simulation training'/exp/mj OR 'high fidelity simulation training'/exp/mj OR 'patient simulation'/exp/mj OR 'virtual reality'/exp/mj OR 'augmented realit*':ti,ab,kw OR 'simulation':ti,ab,kw OR 'simulations':ti,ab,kw OR 'simulate':ti,ab,kw OR 'simulated':ti,ab,kw OR 'sbe':ti,ab,kw OR 'hfst':ti,ab,kw OR 'mixed realit*':ti,ab,kw OR 'virtual placement*':ti,ab,kw OR 'virtual environment*':ti,ab,kw OR 'virtual realit*':ti,ab,kw)	764,477
**2**	('academia'/de OR 'curriculum'/de OR 'education'/de OR 'education' OR 'graduate education'/de OR 'vocational education'/de OR 'interdisciplinary education'/de OR 'learning'/de OR 'teaching'/de OR 'academ*':ti,ab,kw OR 'curric*':ti,ab,kw OR 'education':ti,ab,kw OR 'learning':ti,ab,kw OR 'learner*':ti,ab,kw OR 'interdisciplinary studies':ti,ab,kw OR 'interprofessional':ti,ab,kw OR 'student'/de OR 'students':ti,ab,kw OR 'teaching':ti,ab,kw)	3,639,649
**3**	('collaborative learning'/exp OR 'clinical competence'/exp OR 'placement':ti,ab,kw OR 'placements':ti,ab,kw OR 'program':ti,ab,kw OR 'programs':ti,ab,kw OR 'programme':ti,ab,kw OR 'programmes':ti,ab,kw OR (('clinical':ti,ab,kw OR 'core':ti,ab,kw) AND ('skill':ti,ab,kw OR 'skills*':ti,ab,kw OR 'competen*':ti,ab,kw OR 'expertise*':ti,ab,kw)))	1,751,798
**4**	('paramedical personnel'/de OR 'immunologist'/de OR 'anatomy'/de OR 'anesthesiologist'/de OR 'anesthesist'/de OR 'animal technician'/de OR 'audiologist'/de OR 'cardiologist'/de OR 'caregiver'/de OR 'case manager'/de OR 'health auxiliary'/de OR 'coroner'/de OR 'medical examiners'/de OR 'dental auxiliary'/de OR 'dental auxiliary'/de OR 'dental staff'/de OR 'dental staff'/de OR 'dental staff'/de OR 'dentist'/de OR 'dermatologist'/de OR 'doula'/de OR 'nursing education'/de OR 'pharmacy education'/de OR 'emergency medical dispatcher'/de OR 'rescue personnel'/de OR 'endocrinologist'/de OR 'epidemiologist'/de OR 'dental education'/de OR 'medical school'/de OR 'nursing education'/de OR 'foreign medical graduate'/de OR 'gastroenterologist'/de OR 'general practitioner'/de OR 'geriatrician'/de OR 'gynecologist'/de OR 'health educator'/de OR 'health care facility'/de OR 'health care personnel'/exp OR 'hospital administrator'/de OR 'hospital volunteer'/de OR 'medical staff'/de OR 'infection control practitioner'/de OR 'licensed practical nurse'/de OR 'medical chaperone'/de OR 'clinical laboratory personnel'/de OR 'medical record administrator'/de OR 'medical secretary'/de OR 'medical staff'/de OR 'medical staff'/de OR 'midwife'/de OR 'nephrologist'/de OR 'neurologist'/de OR 'nurse administrator'/de OR 'nurse anesthetist'/de OR 'nurse practitioner'/de OR 'nurse specialist'/de OR 'nurse'/de OR 'nurse'/de OR 'foreign nurse'/de OR 'male nurse'/de OR 'nurse'/de OR 'nursing assistant'/de OR 'nursing staff'/de OR 'nursing staff'/de OR 'nursing staff'/de OR 'dietitian'/de OR 'obstetrician'/de OR 'occupational physician'/de OR 'occupational therapist'/exp OR 'oncologist'/de OR 'operating room personnel'/de OR 'ophthalmologist'/de OR 'optometrist'/de OR 'osteopathic physician'/de OR 'otolaryngologist'/de OR 'paramedical personnel'/de OR 'pathologist'/de OR 'pediatrician'/de OR 'hospital personnel'/de OR 'pharmacist'/de OR 'pharmacy technician'/de OR 'physiatrist'/de OR 'physiotherapist assistant'/de OR 'physiotherapist'/de OR 'medical director'/de OR 'physician assistant'/de OR 'physician'/de OR 'general practitioner'/de OR 'general practitioner'/de OR 'female physician'/de OR 'psychiatrist'/de OR 'psychotherapist'/de OR 'pulmonologist'/de OR 'radiologist'/de OR 'rheumatologist'/de OR 'surgeon'/de OR 'traditional healer'/de OR 'urologist'/de OR 'veterinarian'/de OR 'acupuncturist*':ti,ab,kw OR 'allergist*':ti,ab,kw OR 'allied health':ti,ab,kw OR 'anatomist*':ti,ab,kw OR 'anesthesiologist*':ti,ab,kw OR 'anesthetist*':ti,ab,kw OR 'animal technician*':ti,ab,kw OR 'arts therapist*':ti,ab,kw OR 'audiologist*':ti,ab,kw OR 'cardiologists':ti,ab,kw OR 'caregiver*':ti,ab,kw OR 'case manager*':ti,ab,kw OR 'chiropractor*':ti,ab,kw OR 'community health worker*':ti,ab,kw OR 'coroner*':ti,ab,kw OR 'dental auxiliar*':ti,ab,kw OR 'dental staff':ti,ab,kw OR 'dental assistant*':ti,ab,kw OR 'dental technician*':ti,ab,kw OR 'dental hygienist*':ti,ab,kw OR 'dentist*':ti,ab,kw OR 'dermatologist*':ti,ab,kw OR 'dietician*':ti,ab,kw OR 'dietitian*':ti,ab,kw OR 'dietetic*':ti,ab,kw OR 'doctor*':ti,ab,kw OR 'doula*':ti,ab,kw OR 'emergency medical dispatcher*':ti,ab,kw OR 'emergency medical technician*':ti,ab,kw OR 'endocrinologist*':ti,ab,kw OR 'epidemiologist*':ti,ab,kw OR 'gastroenterologists':ti,ab,kw	4,656,016

	OR 'general practitioner*':ti,ab,kw OR 'geriatrician*':ti,ab,kw OR 'gerontologist*':ti,ab,kw OR 'gynecologist*':ti,ab,kw OR 'health educator*':ti,ab,kw OR 'health facility administrator*':ti,ab,kw OR 'health personnel':ti,ab,kw OR 'health profession*':ti,ab,kw OR 'hospital administrator*':ti,ab,kw OR 'hospitalist*':ti,ab,kw OR 'medical chaperone*':ti,ab,kw OR 'medical examiner*':ti,ab,kw OR 'medical laboratory personnel':ti,ab,kw OR 'medical record administrator*':ti,ab,kw OR 'medical secretar*':ti,ab,kw OR 'medical staff':ti,ab,kw OR 'midwife*':ti,ab,kw OR 'midwifery':ti,ab,kw OR 'midwive*':ti,ab,kw OR 'naturopath*':ti,ab,kw OR 'nephrologist*':ti,ab,kw OR 'neurologist*':ti,ab,kw OR 'nurse*':ti,ab,kw OR 'nursing':ti,ab,kw OR 'nutritionist*':ti,ab,kw OR 'obstetrician*':ti,ab,kw OR 'occupational therapist*':ti,ab,kw OR 'occupational therapy':ti,ab,kw OR 'oncologist*':ti,ab,kw OR 'operating room technician*':ti,ab,kw OR 'ophthalmologist*':ti,ab,kw OR 'optometrist*':ti,ab,kw OR 'optician*':ti,ab,kw OR 'osteopath*':ti,ab,kw OR 'otolaryngologist*':ti,ab,kw OR 'paramedic*':ti,ab,kw OR 'pathologist*':ti,ab,kw OR 'pediatrician*':ti,ab,kw OR 'pharmacist*':ti,ab,kw OR 'pharmacy technician*':ti,ab,kw OR 'pharmacy':ti,ab,kw OR 'physiatr*':ti,ab,kw OR 'physical therapist*':ti,ab,kw OR 'physical therapy':ti,ab,kw OR 'physician*':ti,ab,kw OR 'physiotherap*':ti,ab,kw OR 'podiatrist*':ti,ab,kw OR 'practitioner*':ti,ab,kw OR 'psychiatrist*':ti,ab,kw OR 'psychotherapist*':ti,ab,kw OR 'psychologist*':ti,ab,kw OR 'therapist*':ti,ab,kw OR 'counsellor*':ti,ab,kw OR 'counselor*':ti,ab,kw OR 'pulmonologist*':ti,ab,kw OR 'radiation technician*':ti,ab,kw OR 'radiolog*':ti,ab,kw OR 'rehabilitation':ti,ab,kw OR 'rheumatologist*':ti,ab,kw OR 'social worker*':ti,ab,kw OR 'speech language':ti,ab,kw OR 'speech therapist*':ti,ab,kw OR 'surgeon*':ti,ab,kw OR 'urologist*':ti,ab,kw OR 'veterinarian*':ti,ab,kw)	
**5**	#1 AND #2 AND #3 AND #4	19,004

**CINAHL Plus with Full Text (EBSCO)**<br><br>**Unqualified searches search: author, subject, keywords, title, abstract (https://connect.ebsco.com/s/article/What-is-an-unqualified-search?language=en_US)**		
**1**	("augmented reality" OR "simulation training" OR "high fidelity simulation training" OR "patient simulation" OR "virtual reality" OR "augmented realit*" OR "simulation" OR "simulations" OR "simulate" OR "simulated" OR "SBE" OR "HFST" OR "mixed realit*" OR "virtual placement*" OR "virtual environment*" OR "virtual realit*")	92,125
**2**	("academia" OR "curriculum" OR "education" OR "education"[sh] OR "education, graduate" OR "education, professional" OR "interdisciplinary studies" OR "learning" OR "teaching" OR "academ*" OR "curric*" OR "education" OR "learning" OR "learner*" OR "interdisciplinary studies" OR "interprofessional" OR "students" OR "students" OR "teaching")	1,193,923
**3**	("Interdisciplinary Placement" OR "Clinical Competence" OR "placement" OR "placements" OR "program" OR "programs" OR "programme" OR "programmes" OR (("clinical" OR "core") AND ("skill" OR "skills*" OR "competen*" OR "expertise*")))	701,264
**4**	("allied health personnel" OR "allergists" OR "anatomists" OR "anesthesiologists" OR "anesthetists" OR "animal technicians" OR "audiologists" OR "cardiologists" OR "caregivers" OR "case managers" OR "community health workers" OR "coroners and medical examiners" OR "dental auxiliaries" OR "dental auxiliaries" OR "dental staff" OR "dental staff, hospital" OR "dental staff, hospital" OR "dentists" OR "dermatologists" OR "doulas" OR "education, nursing, baccalaureate" OR "education, pharmacy" OR "emergency medical dispatcher" OR "emergency medical technicians" OR "endocrinologists" OR "epidemiologists" OR "faculty, dental" OR "faculty, medical" OR "faculty, nursing" OR "foreign medical graduates" OR "gastroenterologists" OR "general practitioners" OR "geriatricians" OR "gynecologists" OR "health educators" OR "health facility administrators" OR "health personnel" OR "hospital administrators" OR "hospital volunteers" OR "hospitalists" OR "infection control practitioners" OR "licensed practical nurses" OR "medical chaperones" OR "medical laboratory personnel" OR "medical record administrators" OR "medical secretaries" OR "medical staff" OR "medical staff, hospital" OR "midwifery" OR "nephrologists" OR "neurologists" OR "nurse administrators" OR "nurse anesthetists" OR "nurse practitioners" OR "nurse specialists" OR "nurses" OR "nurses, community health" OR "nurses, international" OR "nurses, male" OR "nurses, public health" OR "nursing assistants" OR "nursing staff" OR "nursing staff, hospital" OR "nursing staff, hospital" OR "nutritionists" OR "obstetricians" OR "occupational health physicians" OR "occupational therapists"[mesh] OR "oncologists" OR "operating room technicians" OR "ophthalmologists" OR "optometrists" OR "osteopathic	2,165,297

	physicians" OR "otolaryngologists" OR "paramedics" OR "pathologists" OR "pediatricians" OR "personnel, hospital" OR "pharmacists" OR "pharmacy technicians" OR "physiatrists" OR "physical therapist assistants" OR "physical therapists" OR "physician executives" OR "physician assistants" OR "physicians" OR "physicians, family" OR "physicians, primary care" OR "physicians, women" OR "psychiatrists" OR "psychotherapists" OR "pulmonologists" OR "radiologists" OR "rheumatologists" OR "surgeons" OR "traditional medicine practitioners" OR "urologists" OR "veterinarians" OR "acupuncturist*" OR "allergist*" OR "allied health" OR "anatomist*" OR "anesthesiologist*" OR "anesthetist*" OR "animal technician*" OR "arts therapist*" OR "audiologist*" OR "cardiologists" OR "caregiver*" OR "case manager*" OR "chiropractor*" OR "community health worker*" OR "coroner*" OR "dental auxiliar*" OR "dental staff" OR "dental assistant*" OR "dental technician*" OR "dental hygienist*" OR "dentist*" OR "dermatologist*" OR "dietician*" OR "dietitian*" OR "dietetic*" OR "doctor*" OR "doula*" OR "emergency medical dispatcher*" OR "emergency medical technician*" OR "endocrinologist*" OR "epidemiologist*" OR "gastroenterologists" OR "general practitioner*" OR "geriatrician*" OR "gerontologist*" OR "gynecologist*" OR "health educator*" OR "health facility administrator*" OR "health personnel" OR "health profession*" OR "hospital administrator*" OR "hospitalist*" OR "medical chaperone*" OR "medical examiner*" OR "medical laboratory personnel" OR "medical record administrator*" OR "medical secretar*" OR "medical staff" OR "midwife*" OR "midwifery" OR "midwive*" OR "naturopath*" OR "nephrologist*" OR "neurologist*" OR "nurse*" OR "nursing" OR "nutritionist*" OR "obstetrician*" OR "occupational therapist*" OR "occupational therapy" OR "oncologist*" OR "operating room technician*" OR "ophthalmologist*" OR "optometrist*" OR "optician*" OR "osteopath*" OR "otolaryngologist*" OR "paramedic*" OR "pathologist*" OR "pediatrician*" OR "pharmacist*" OR "pharmacy technician*" OR "pharmacy" OR "physiatr*" OR "physical therapist*" OR "physical therapy" OR "physician*" OR "physiotherap*" OR "podiatrist*" OR "practitioner*" OR "psychiatrist*" OR "psychotherapist*" OR "psychologist*" OR "therapist*" OR "counsellor*" OR "counselor*" OR "pulmonologist*" OR "radiation technician*" OR "radiolog*" OR "rehabilitation" OR "rheumatologist*" OR "social worker*" OR "speech language" OR "speech therapist*" OR "surgeon*" OR "urologist*" OR "veterinarian*")	
**5**	#1 AND #2 AND #3 AND #4	9,120

PsycINFO (EBSCO)

Unqualified searches search: author, subject, keywords, title, abstract (https://connect.ebsco.com/s/article/What-is-an-unqualified-search?language=en_US)

**1**	("augmented reality" OR "simulation training" OR "high fidelity simulation training" OR "patient simulation" OR "virtual reality" OR "augmented realit*" OR "simulation" OR "simulations" OR "simulate" OR "simulated" OR "SBE" OR "HFST" OR "mixed realit*" OR "virtual placement*" OR "virtual environment*" OR "virtual realit*")	104,439
**2**	("academia" OR "curriculum" OR "education" OR "education"[sh] OR "education, graduate" OR "education, professional" OR "interdisciplinary studies" OR "learning" OR "teaching" OR "academ*" OR "curric*" OR "education" OR "learning" OR "learner*" OR "interdisciplinary studies" OR "interprofessional" OR "students" OR "students" OR "teaching")	1,686,778
**3**	("Interdisciplinary Placement" OR "Clinical Competence" OR "placement" OR "placements" OR "program" OR "programs" OR "programme" OR "programmes" OR (("clinical" OR "core") AND ("skill" OR "skills*" OR "competen*" OR "expertise*")))	723,690
**4**	("allied health personnel" OR "allergists" OR "anatomists" OR "anesthesiologists" OR "anesthetists" OR "animal technicians" OR "audiologists" OR "cardiologists" OR "caregivers" OR "case managers" OR "community health workers" OR "coroners and medical examiners" OR "dental auxiliaries" OR "dental auxiliaries" OR "dental staff" OR "dental staff, hospital" OR "dental staff, hospital" OR "dentists" OR "dermatologists" OR "doulas" OR "education, nursing, baccalaureate" OR "education, pharmacy" OR "emergency medical dispatcher" OR "emergency medical technicians" OR "endocrinologists" OR "epidemiologists" OR "faculty, dental" OR "faculty, medical" OR "faculty, nursing" OR "foreign medical graduates" OR "gastroenterologists" OR "general practitioners" OR "geriatricians" OR "gynecologists" OR "health educators" OR "health facility administrators" OR "health personnel" OR "hospital administrators" OR "hospital volunteers" OR "hospitalists" OR "infection control practitioners" OR "licensed practical nurses" OR "medical chaperones" OR "medical laboratory personnel" OR "medical record administrators" OR "medical secretaries" OR "medical staff" OR "medical staff, hospital" OR "midwifery" OR "nephrologists" OR	1,087,106

	"neurologists" OR "nurse administrators" OR "nurse anesthetists" OR "nurse practitioners" OR "nurse specialists" OR "nurses" OR "nurses, community health" OR "nurses, international" OR "nurses, male" OR "nurses, public health" OR "nursing assistants" OR "nursing staff" OR "nursing staff, hospital" OR "nursing staff, hospital" OR "nutritionists" OR "obstetricians" OR "occupational health physicians" OR "occupational therapists"[mesh] OR "oncologists" OR "operating room technicians" OR "ophthalmologists" OR "optometrists" OR "osteopathic physicians" OR "otolaryngologists" OR "paramedics" OR "pathologists" OR "pediatricians" OR "personnel, hospital" OR "pharmacists" OR "pharmacy technicians" OR "physiatrists" OR "physical therapist assistants" OR "physical therapists" OR "physician executives" OR "physician assistants" OR "physicians" OR "physicians, family" OR "physicians, primary care" OR "physicians, women" OR "psychiatrists" OR "psychotherapists" OR "pulmonologists" OR "radiologists" OR "rheumatologists" OR "surgeons" OR "traditional medicine practitioners" OR "urologists" OR "veterinarians" OR "acupuncturist*" OR "allergist*" OR "allied health" OR "anatomist*" OR "anesthesiologist*" OR "anesthetist*" OR "animal technician*" OR "arts therapist*" OR "audiologist*" OR "cardiologists" OR "caregiver*" OR "case manager*" OR "chiropractor*" OR "community health worker*" OR "coroner*" OR "dental auxiliar*" OR "dental staff" OR "dental assistant*" OR "dental technician*" OR "dental hygienist*" OR "dentist*" OR "dermatologist*" OR "dietician*" OR "dietitian*" OR "dietetic*" OR "doctor*" OR "doula*" OR "emergency medical dispatcher*" OR "emergency medical technician*" OR "endocrinologist*" OR "epidemiologist*" OR "gastroenterologists" OR "general practitioner*" OR "geriatrician*" OR "gerontologist*" OR "gynecologist*" OR "health educator*" OR "health facility administrator*" OR "health personnel" OR "health profession*" OR "hospital administrator*" OR "hospitalist*" OR "medical chaperone*" OR "medical examiner*" OR "medical laboratory personnel" OR "medical record administrator*" OR "medical secretar*" OR "medical staff" OR "midwife*" OR "midwifery" OR "midwive*" OR "naturopath*" OR "nephrologist*" OR "neurologist*" OR "nurse*" OR "nursing" OR "nutritionist*" OR "obstetrician*" OR "occupational therapist*" OR "occupational therapy" OR "oncologist*" OR "operating room technician*" OR "ophthalmologist*" OR "optometrist*" OR "optician*" OR "osteopath*" OR "otolaryngologist*" OR "paramedic*" OR "pathologist*" OR "pediatrician*" OR "pharmacist*" OR "pharmacy technician*" OR "pharmacy" OR "physiatr*" OR "physical therapist*" OR "physical therapy" OR "physician*" OR "physiotherap*" OR "podiatrist*" OR "practitioner*" OR "psychiatrist*" OR "psychotherapist*" OR "psychologist*" OR "therapist*" OR "counsellor*" OR "counselor*" OR "pulmonologist*" OR "radiation technician*" OR "radiolog*" OR "rehabilitation" OR "rheumatologist*" OR "social worker*" OR "speech language" OR "speech therapist*" OR "surgeon*" OR "urologist*" OR "veterinarian*")	
**5**	#1 AND #2 AND #3 AND #4	3,333

Right-hand search through academic journal results

We hand-searched the *Focus on Health Professional Education: A Multi-Professional Journal* as it is a popular journal that is not currently indexed. This identified 50 results, and we included 2 from these.

Date searched: August 5, 2024

Search term 'placement'		
Results: 30		
No.	Title	Link
1	Establishing and sustaining a new interprofessional allied health student placement	https://fohpe.org/FoHPE/article/view/330
2	Providing quality allied health placements in palliative care	https://fohpe.org/FoHPE/article/view/133
3	The big city by-pass: Origin is important in medical students' preference for future practice in regional cities and large towns	https://fohpe.org/FoHPE/article/view/187
4	Simulation speaks for itself: Building speech-language pathology students' confidence through high quality simulation within a workplace clinical placement	https://fohpe.org/FoHPE/article/view/218
5	Words in action: Examining what clinical education placement documents contribute to thinking, acting and feeling like a health professional	https://fohpe.org/FoHPE/article/view/380
6	Clinical supervisors' perceptions of podiatry students' preparedness for clinical placement and graduates'	https://fohpe.org/FoHPE/article/view/339

	preparedness for podiatry practice in Australia: An exploratory study	
7	A snapshot of placement activities and learning outcomes assessed through logbook submission for clinical exercise physiology students	https://fohpe.org/FoHPE/article/view/208
8	Lining up the ducks: aligning the hidden, formal and informal curricula in an immersed learning environment	https://fohpe.org/FoHPE/article/view/229
9	Novel interprofessional learning for healthcare students: An escape room pilot	https://fohpe.org/FoHPE/article/view/306
10	The quantitative impact of placements on allied health time use and productivity in healthcare facilities: a systematic review with meta-analysis	https://fohpe.org/FoHPE/article/view/315
11	Preparing dental graduates to provide care for frail and care-dependent older patients: An educational intervention	https://fohpe.org/FoHPE/article/view/419
12	Report: The University Health Clinic: Definition, educational practices and outcomes	https://fohpe.org/FoHPE/article/view/213
13	Practice performance and performance anxiety: Preparing osteopathic students for practice	https://fohpe.org/FoHPE/article/view/196
14	"Group drum helps placement working": Unpacking the value of DRUMBEAT for interprofessional group learning during practice placement	https://fohpe.org/FoHPE/article/view/633
15	The health service impact of an occupational therapy practice placement model: Student-resourced service delivery of groups	https://fohpe.org/FoHPE/article/view/525
16	A student-led, interprofessional care, community-based healthcare service: Student, clinical educator and client perceptions of interprofessional care and education	https://fohpe.org/FoHPE/article/view/476
17	Paramedic student and preceptor experiences of a clinical facilitator model during ambulance clinical placements: A qualitative study	https://fohpe.org/FoHPE/article/view/761
18	Peer mentoring for clinical educators: A case study in physiotherapy	https://fohpe.org/FoHPE/article/view/175
19	Navigating the opportunities of service-learning placements: An exploration of benefits to host organisations of allied health service-learning placements	https://fohpe.org/FoHPE/article/view/733
20	Models of pre-registration student supervision in allied health: a scoping review	https://fohpe.org/FoHPE/article/view/559
21	Discussion Paper: Children in out-of-home care: What do medical students learn about them?	https://fohpe.org/FoHPE/article/view/199
22	Factors influencing provision of clinical placements for health students: A scoping review	https://fohpe.org/FoHPE/article/view/562
23	Physiotherapy students’ perceptions of engagement with people from culturally and linguistically diverse communities during clinical placement	https://fohpe.org/FoHPE/article/view/748
24	Co-designing an educational escape room: Integrating the knowledge and perspective of Aboriginal and Torres Strait Islander people with the design of an educational activity for healthcare students	https://fohpe.org/FoHPE/article/view/533
25	The transformation of a student-led health clinic in rural Australia from a face-to-face service to a telehealth model: Evaluation of student and client experiences during a COVID 19 driven transition	https://fohpe.org/FoHPE/article/view/554
26	Health professional service-learning innovations in rural contexts	https://fohpe.org/FoHPE/article/view/644
27	The role of simulation in developing clinical knowledge and increasing clinical confidence in first-year radiography students	https://fohpe.org/FoHPE/article/view/83
28	A survey of strategies for increasing the number of medical learners in all Tasmanian general practices	https://fohpe.org/FoHPE/article/view/225
29	Enhancing patient education skills of physiotherapy students in a clinical education setting	https://fohpe.org/FoHPE/article/view/205
30	A collaborative approach to engaging undergraduate students in learning and developing evidence-based practice	https://fohpe.org/FoHPE/article/view/575
Search term 'simulation'		
Results: 20		
No.	Title	Link

1	The use of interprofessional simulation interventions in medical student education: A scoping review	https://fohpe.org/FoHPE/article/view/459
2	Take-home laparoscopic simulators to develop surgical skills: Analysing attitudes to, and barriers and enablers of, their use in gynaecology training	https://fohpe.org/FoHPE/article/view/352
3	Simulation speaks for itself: Building speech-language pathology students' confidence through high quality simulation within a workplace clinical placement	https://fohpe.org/FoHPE/article/view/218
4	Benefits of providing an acute simulated learning environment to speech pathology students: An exploratory study	https://fohpe.org/FoHPE/article/view/186
5	Focus on Methodology: Eliciting rich data: A practical approach to writing semi-structured interview schedules	https://fohpe.org/FoHPE/article/view/387
6	Using OSCEs with simulation to maximise student learning and assess competencies in psychology: A pilot study	https://fohpe.org/FoHPE/article/view/140
7	The role of simulation in developing clinical knowledge and increasing clinical confidence in first-year radiography students	https://fohpe.org/FoHPE/article/view/83
8	Evaluation of affective learning in a gamified pharmacy simulation	https://fohpe.org/FoHPE/article/view/572
9	Mask-EdTM: A scoping review	https://fohpe.org/FoHPE/article/view/414
10	Interprofessional learning in general practice: A pilot study using in-practice emergency simulation	https://fohpe.org/FoHPE/article/view/89
11	Impact of vicarious learning through peer observation during simulation on student behavioural measures	https://fohpe.org/FoHPE/article/view/99
12	Speech pathology students' perceived learning outcomes following participation in a fluency booster program	https://fohpe.org/FoHPE/article/view/412
13	First-year physiotherapy students who elect to participate in simulation-based learning activities benefit from the experience	https://fohpe.org/FoHPE/article/view/109
14	The use of clinical simulation to support development of interprofessional skills and understanding: Perspectives from allied health students	https://fohpe.org/FoHPE/article/view/616
15	Nurses' experiences in a rural interprofessional simulation course	https://fohpe.org/FoHPE/article/view/577
16	Developing palliative care skills in undergraduate allied health students using an interprofessional simulation-based learning experience	https://fohpe.org/FoHPE/article/view/718
17	Enhancing patient education skills of physiotherapy students in a clinical education setting	https://fohpe.org/FoHPE/article/view/205
18	Skills for the aspiring surgeon in Australia: A needs assessment	https://fohpe.org/FoHPE/article/view/658
19	A collaborative approach to engaging undergraduate students in learning and developing evidence-based practice	https://fohpe.org/FoHPE/article/view/575
20	Recommendations for the design of interprofessional education: Findings from a narrative scoping review	https://fohpe.org/FoHPE/article/view/608

Supplemental search

We conducted a supplemental search since we did not do forwards and backwards citation searching on all of our included sources. We went through all of our included reviews and collated a list of all the studies that these reviews had included. Two independent reviewers then screened through these studies, and resolved any conflicts at the end through discussion and a third reviewer.

List of reviews

Bogossian (2019) Locating "gold standard" evidence for simulation as a substitute for clinical practice in prelicensure health professional education: A systematic review
Cant (2017) Use of simulation-based learning in undergraduate nurse education: An umbrella systematic review
Grant (2021) The use of simulation in occupational therapy education: A scoping review
Nagdee (2022) Simulations as a mode of clinical training in healthcare professions: A scoping review to guide planning in speech-language pathology and audiology during the COVID-19 pandemic and beyond
Roberts (2019) Simulation to Replace Clinical Hours in Nursing: A Meta-narrative Review
Samson (2023) Virtual Simulated Placements in Healthcare Education: A scoping review
Squires (2022) Mapping Simulated-Based Learning Experiences Incorporated Into Professional Placements in Allied Health Programs: A Scoping Review
Larue (2015) Simulation in preparation or substitution for clinical placement: A systematic review of the literature

Lobão (2023) Changes in Clinical Training for Nursing Students during the COVID-19 Pandemic: A Scoping Review
NARRATIVE REVIEW NOT SCREENED: Jimenez (2023) Can simulation-based education or other education interventions replace clinical placement in medical radiation sciences? A narrative review
NARRATIVE REVIEW NOT SCREENED: Wood (2019) Myotherapy student clinical placements: A review beyond the teaching clinic

Studies screened after duplicates removed

Number of duplicates removed: 13

Total included: 17

1	Adamson, K., 2015. A systematic review of the literature related to the NLN/Jeffries simulation framework. Nurs. Educ. Perspect. 36, 281–291.	Exclude
2	Al-Dahir S, Bryant K, Kennedy KB, Robinson DS. Online virtual-patient cases versus traditional problem-based learning in advanced pharmacy practice experiences. Am J Pharm Educ 2014;78(4):76.	Exclude
3	Alpert J, Young M, Lala S, et al. Medical student engagement and educational value of a remote clinical radiology learning environment: creation of virtual read-out sessions in response to the COVID-19 pandemic. Academic Radiology 2021; 28:112-118.	Exclude
4	Au, M. L., Lo, M. S., Cheong, W., Wang, S. C., & Van, I. K. (2016). Nursing students' perception of high-fidelity simulation activity instead of clinical placement: A qualitative study. Nurse Education Today, 39(Supplement C), 16-21. https://doi.org/10.1016/j.nedt.2016.01.015.	Excluded at full text in covidence
5	Baillie, L., & Curzio, J. (2009). Students' and facilitators' perceptions of simulation in practice learning. Nurse Education in Practice, 9, 297– 306. https://doi.org/10.1016/j.nepr.2008.08.007	**Include**
6	Barker M, Fejzic J, Mak AS. Simulated learning for generic communication competency development: a case study ofAustralian postgraduate pharmacy students. Higher Educ Res Dev 2018;37(6): 1109–1123.	Exclude
7	Beddingfield, S., Davis, B. W., Gilmore, M., & Jenkins, L. (2011). The effect of high-fidelity simulation on examination performance. Teaching and Learning in Nursing, 6(2), 46-49. https://doi.org/10.1016/j.teln.2010.10.001	Exclude
8	Bennett, S., Rodger, S., Fitzgerald, C., & Gibson, L. (2017). Simulation in occupational therapy curricula: A literature review. Australian Occupational Therapy Journal, 64(4), 314–327. https://doi.org/10.1111/1440-1630.12372	Exclude
9	Berndt, J., 2014. Patient safety and simulation in prelicensure nursing education: an integrative review. Teach. Learn. Nurs. 9, 16–22.	Exclude
10	Bethea, D. P., Castillo, D. C., & Harvison, N. (2014). Use of simulation in occupational therapy education: Way of the future? American Journal of Occupational Therapy, 68(S2), S32–S39. https://doi.org/10.5014/ajot.2014.012716	Exclude
11	Bhashyam A, Dyer, G. "Virtual" boot camp: Orthopaedic intern education in the time of COVID-19 and beyond. Journal of the American Academy of Orthopaedic Surgeons 2020; 28(17), e735-e743. doi: 10.5435/JAAOS-D-20-00559.	Exclude
12	Blackford J, Alison J, McAllister L, Nisbet G. The clinical educators' experience of replacing physiotherapy student clinical education time with simulation. Int J Pract Based LearnHealth Soc Care 2020;8(1):60–72.	**Include**
13	Blackford J, McAllister L, Alison J. Simulated learning in the clinical education of novice physiotherapy students. Int J Pract Based Learn Health Soc Care 2015;3(1):77–93.	**Include**
14	Blackstock FC, Watson KM, Morris NR, et al. Simulation can contribute a part of cardiorespiratory physiotherapy clinical education: two randomized trials. Simul Healthc 2013;8(1):32–42.	Already included
15	Blum, C.A., Parcells, D.A., 2012. Relationship between high-fidelity simulation and patient safety in prelicensure nursing education: a comprehensive review. J. Nurs. Educ. 51(8):429–435. http://dx.doi.org/10.3928/01484834-20120523-01.	Exclude
16	Boudiche, S., Zelfani, S., Hammamia, M.B., Mghaieth, F., Ouaghlani, K., Halima, M.B., ... Mourali, M.S. (2020). Simulation training for continuing professional development of nurses in cardiology and cardiovascular surgery. La Tunisie Medicale, 98(2), 116–122. Retrieved from https://pubmed.ncbi.nlm.nih.gov/32395800/	Exclude

17	Bradley, G., Whittington, S., & Mottram, P. (2013). Enhancing occupational therapy education through simulation. British Journal of Occupational Therapists, 76(1), 43–46. https://doi.org/10.4276/030802213X13576469254775	Exclude
18	Brien, L.-A., Charette, M., & Goudreau, J. (2017). Nursing students’ perceptions of the contribution of high-fidelity simulation and clinical placement in a critical care course. Clinical Simulation in Nursing, 13(9), 436-441. https://doi.org/10.1016/j.ecns.2017.05.005.	Already included
19	Brown T,Williams B. The use ofDVD simulation as an interprofessional education tool with undergraduate occupational therapy students. Br J Occup Ther 2009;72(6):266–274.	Exclude
20	Büyük, S., Bermede, O., Erkoç, S., Alkış, N., Lilot, M., & Meço, B. (2021). Use of simulation to teach in the operating room: Don’t let the COVID-19 pandemic to interrupt education: An observational clinical trial. Brazilian Journal of Anesthesiology (English Edition), 72(2), 185–188. https://doi.org/10.1016/j.bjane.2021.11.010	Exclude
21	Cahill, S. M. (2015). Perspectives on the use of standardized parents to teach collaboration to graduate occupational therapy students. American Journal of Occupational Therapy, 69, https://doi.org/10.5014/ajot.2015.017103.	Exclude
22	Cant, R.P., & Cooper, S.J. (2017). The value of simulation-based learning in pre- licensure nurse education: A state-of-the-art review and meta-analysis. Nurse Education in Practice, 27, 45–62. https://doi.org/10.1016/j.nepr.2017.08.012	Exclude
23	Cant, R.P., Cooper, S.J., 2010. Simulation-based learning in nurse education: systematic review. J. Adv. Nurs. 66:3–15. http://dx.doi.org/10.1111/j.1365-2648.2009.<br>05240.x.	Exclude
24	Cant, R.P., Cooper, S.J., 2014. Simulation in the internet age: the place of web-based simulation in nursing education. An integrative review. Nurse Educ. Today 34:<br>1435–1442. http://dx.doi.org/10.1016/j.nedt.2014.1408.1001.	Exclude
25	Choong, J., & Tan, Z.Y. (2019). The role of simulation in burns education. British Journal of Hospital Medicine, 80(12), 716–719. https://doi.org/10.12968/hmed.2019.80.12.716	Exclude
26	Creagh S, Pigg N, Gordillo C, et al. Virtual medical student radiology clerkships during the COVID-19 pandemic: Distancing is not a barrier. Clinical Imaging 2021; 80: 420-423.	**Include**
27	Curl, E. D., Smith, S., Chisholm, L. A., McGee, L. A., & Das, K. (2016). Effectiveness of integrated simulation and clinical experiences compared to traditional clinical experiences for nursing students. Nursing Education Perspectives, 37(2), 72-77. https://doi.org/10.5480/15-1647.	Already included
28	Datta, R., Upadhyay, K., & Jaideep, C. (2012). Simulation and its role in medical education. Medical Journal Armed Forces India, 68(2), 167–172. https://doi.org/10.1016/S03771237(12)60040-9	Exclude
29	De Ponti, R., Marazzato, J., Maresca, A.M., Rovera, F., Carcano, G., & Ferrario, M.M. (2020). Pre-graduation medical training including virtual reality during COVID-19 pandemic: A report on students’ perception. BMC Medical Education, 20(1), 332. https://doi.org/10.1186/s12909-020-02245-8	Exclude
30	Durfee S, Goldenson R, Gill R, et al. Medical student education roadblock due to COVID-19: Virtual radiology core clerkship to the rescue. Academic Radiology 2020. 27(10),1461-1466.	**Include**
31	Eide, W.M., Johansson, L., & Eide, L.S.P. (2020). First-year nursing students’ experiences of simulation involving care of older patients. A descriptive and exploratory study. Nurse Education in Practice, 45, 102797. https://doi.org/10.1016/j.nepr.2020.102797	Exclude
32	Elshami W, Abuzaid M. Transforming magnetic resonance imaging education through simulation-based training. J Med Imaging Radiat Sci 2017;48(2):151–158.	Exclude
33	Eng AJ, Namba JM, Box KW, et al. High-fidelity simulation training in advanced resuscitation for pharmacy residents. Am J Pharm Educ 2014; 78(3):59.	Exclude
34	Farahat E, Rice G, Daher N, Heine N, Schneider L, Connell B. Objective Structured Clinical Examination (OSCE) improves perceived readiness for clinical placement in nutrition and dietetic students. JAlliedHealth 2015;44(4):208–214.	Exclude
35	Fehl M, Gehres V, Geier A, et al. Medical students' adoption and evaluation of a completely digital general practice clerkship - cross-sectional survey and cohort comparison with face-to-face teaching. Medical Education Online 2022;27(1): 2028334. Doi: 10.1080/10872981.2022.2028334.	**Include**
36	Fejzic J, Barker M, Hills R, Priddle A. Communication capacity building through pharmacy practice simulation. Am J Pharm Educ 2016;80(2):28.	Exclude

37	Fejzic J, Barker M. Implementing simulated learning modules to improve students' pharmacy practice skills and professionalism. Pharm Pract (Granada) 2015;13(3):583.	Exclude
38	Fisher, D., King, L., 2013. An integrative literature review on preparing nursing students through simulation to recognize and respond to the deteriorating patient. J. Adv. Nurs. 69, 2375–2388.	Exclude
39	Foronda, C., Liu, S., Bauman, E.B., 2013. Evaluation of simulation in undergraduate nurse education: an integrative review. Clin. Simul. Nurs. 9, e409–e416.	Exclude
40	Franklin, A.E., Lee, C.S., 2014. Effectiveness of simulation for improvement in self-efficacy among novice nurses: a meta-analysis. J. Nurs. Educ. 53:607–614. http://dx.doi.org/ 10.3928/01484834–20141023-01484803.	Exclude
41	Ganji J, Shirvani M, Motahari-Tabari N, et al. & Tayebi, T. (2022). Design, implementation and evaluation of a virtual clinical training protocol for midwifery internship in a gynecology course during COVID-19 pandemic: A semi-experimental study. Nurse Education Today 2022;111:105293. doi: 10.1016/j.nedt.2022.105293.	Exclude
42	Gee, B. M., Thompson, K., Strickland, J., & Miller, L. J. (2017). The development of a measurement tool evaluating knowledge related to sensory processing among graduate occupational therapy students: A process description. Occupational Therapy International, 2017, https://doi.org/10.1155/2017/6713012	Exclude
43	Gibbs H, George K, Barkley R, Meyer M. Using multiple-patient simulations to facilitate interprofessional communication between dietetic and nursing students and improve nutrition care process skills. Top Clin Nutr 2015;30(3):230–238.	Exclude
44	Gibbs, D. M., Dietrich, M., & Dagnan, E. (2017). Using high fidelity simulation to impact occupational therapy student knowledge, comfort, and confidence in acute care. The Open Journal of Occupational Therapy, 5, 1. https://doi.org/10.15453/2168-6408.1225	Exclude
45	Giblett, N., Rathore, R., & Carruthers, D. (2017). Simulating the surgical patient pathway for undergraduates. Journal of Surgical Education, 74, 271–276. 210.1016/j.jsurg.2016.1010.1003	Exclude
46	Giles, A. K., Carson, N. E., Breland, H. L., Coker-Bolt, P., & Bowman, P. J. (2014). Use of simulated patients and reflective video analysis to assess occupational therapy students' preparedness for fieldwork. American Journal of Occupational Therapy, 68, S57–S66. https://doi.org/10.5014/ajot.2014.685S03	Exclude
47	Gomez E, Azadi J, Magid D. Innovation born in isolation: Rapid transformation of an in person medical student radiology elective to a remote learning experience during the COVID-19 pandemic. Academic Radiology 2020; 27(9):1285-1290.	Exclude
48	Grabowski, A., Chuisano, S.A., Strock, K., Zielinski, R., Anderson, O.S., & Sadovnikova, A. (2021). A pilot study to evaluate the effect of classroom-based high-fidelity simulation on midwifery students' self-efficacy in clinical lactation and perceived translation of skills to the care of the breastfeeding mother-infant dyad. Midwifery, 102, 103078. https://doi.org/10.1016/j.midw.2021.103078	Exclude
49	Green D, Appleyard R. The influence ofVERT™ characteristics on the development of skills in skin apposition techniques. Radiography 2011; 17(3):178–182.	Exclude
50	Gunn, T., Rowntree, P., Starkey, D., & Nissen, L. (2021). The use of virtual reality computed tomography simulation within a medical imaging and a radiation therapy undergraduate programme. Journal of Medical Radiation Sciences, 68(1), 28–36. https://doi.org/10.1002/jmrs.436	Exclude
51	Haracz, K., Arrighi, G., & Joyce, B. (2015). Simulated patients in a mental health occupational therapy course: A pilot study. British Journal of Occupational Therapy, 78(12), 757–766. https://doi.org/10.1177/0308022614562792	Exclude
52	Hayden, J., Smiley, R., Alexander, M., Kardong-Edgren, S., & Jeffries, P. (2014b). The NCSBN national simulation study: A longitudinal, randomized, controlled study replacing clinical hours with simulation in prelicensure nursing education. Journal of Nursing Regulation, 5(2), S3-S40. https://doi.org/10.1016/S2155-8256(15)30062-4.	Already included
53	He M, Tang X, Zhang H, et al. Remote clinical training practice in the neurology internship during the COVID-19 pandemic. Medical Education Online 2021;26:1899642. doi: 10.1080/10872981.2021.1899642.	Exclude

54	Henry BW, Duellman MC, Smith TJ. Nutrition-based standardised patient sessions increased counseling awareness and confidence among dietetic interns. Top Clin Nutr 2009;24(1):25–34.	Exclude
55	Hernández-Padilla, J.M., Granero-Molina, J., Márquez-Hernández, V.V., Cortés- Rodríguez, A.E., & Fernández-Sola, C. (2016). Effects of a simulation-based workshop on nursing students' competence in arterial puncture. Acta Paulista de Enfermagem, 29(6), 678–685. https://doi.org/10.1590/1982-0194201600095	Exclude
56	Hill AE, Davidson BJ, Theodoros DG. Speech-language pathology students' perceptions ofa standardised patient clinic. JAlliedHealth 2013; 42(2):84–91.	Already included
57	Hill AE, Ward E, Heard R, et al. Simulation can replace part of speech- language pathology placement time: a randomised controlled trial. Int J Speech Lang Pathol 2020;23:92–102.	Exclude
58	Holmberg M, Dela Cruz E, Longino A, et al. Development of a single-institution virtual internal medicine subinternship with near-peer teaching in response to the COVID-19 pandemic. Academic Medicine 2021;96(12):1706-1710.	Exclude
59	Hu, F., Yang, J., Yang, B.X., Zhang, F.-J., Yu, S.-H., Liu, Q., ... Chen, J. (2021). The impact of simulation-based triage education on nursing students' self-reported clinical reasoning ability: A quasi-experimental study. Nurse Education in Practice, 50, 102949. https://doi.org/10.1016/j.nepr.2020.102949	Exclude
60	Imms, C., Chu, E. M. Y., Guinea, S., Sheppard, L., Froude, E., Carter, R., Darzins, S., Ashby, S., Gilbert-Hunt, S., Gribble, N., NicolaRichmond, K., Penman, M., Gospodarevskaya, E., Mathieu, E., & Symmons, M. (2017). Effectiveness and cost-effectiveness of embedded simulation in occupational therapy clinical practice education: Study protocol for a randomised controlled trial. Trials, 18(1), 17–29. https://doi.org/10.1186/s13063-017-2087-0	Already included
61	Imms, C., Froude, E., Chu, E. M. Y., Sheppard, L., Darzins, S., Guinea, S., Gospodarevskaya, E., Carter, R., Symmons, M. A., Penman, M., Nicola-Richmond, K., Gilbert Hunt, S., Gribble, N., Ashby, S., & Mathieu, E. (2018). Simulated versus traditional occupational therapy placements: A randomised controlled trial. Australian Occupational Therapy Journal, 65(6), 556–564. https://doi.org/10.1111/1440-1630.12513	Exclude
62	Johnson, R.L., Cannon, E.K., Mantilla, C.B., & Cook, D.A. (2013). Cricoid pressure training using simulation: A systematic review and meta-analysis. British Journal of Anaesthesia, 111(3), 338–346. https://doi.org/10.1093/bja/aet121	Exclude
63	Johnston CLP, Wilson JCB, Wakely LP, Walmsley SP, Newstead CJB. Simulation as a component of introductory physiotherapy clinical placements. NZ J Physiother 2018;46(3):95–104.	Exclude
64	Joung J, Kang K. Can Virtual Simulation Replace Clinical Practical Training for Psychiatric Nursing? Issues Ment Health Nursing 2022;43(8):706-711.	Already included
65	Judd BK, Alison JA, Waters D, Gordon CJ. Comparison of psychophysiological stress in physiotherapy students undertaking simulation and hospital-based clinical education. Simul Healthc 2016; 11(4):271–277.	**Include**
66	Kapoor, A., Kapoor, A., & Badyal, D.K. (2021). Simulated patients for competency- based undergraduate medical education post COVID-19: A new normal in India. Indian Pediatrics, 58(9), 881–887. https://doi.org/10.1007/s13312-021-2312-5	Exclude
67	Kasai H, Shikino K, Saito G, et al. Alternative approaches for clinical clerkship during the COVID-19 pandemic: online simulated clinical practice for inpatients and outpatients-A mixed method. BMC Medical Education 2021;21(1):149. doi: 10.1186/s12909-021-02586-y.	**Include**
68	Kelly DG, Brown DS, Perritt L, Gardner DL. A descriptive study comparing achievement of clinical education objectives and clinical performance between students participating in traditional and mock clinics. J Phys Ther Educ 1996;10(1):26–31.	Already included
69	Kelly T, Surjan Y, Rinks M, Warren-Forward H. Effect of communication skills training on radiation therapy student's confidence and interactions during their first clinical placement. Radiography (Lond) 2021;27:59–66.	Exclude

70	Ketterer SJ, Callender J, Warren M, et al. Simulated versus traditional therapeutic radiography placements: a randomised controlled trial. Radiography (Lond) 2020;26(2):140–146.	Already included
71	Kimhi, E., Reishtein, J. L., Cohen, M., Friger, M., Hurvitz, N., & Avraham, R. (2016). Impact of simulation and clinical experience on self-efficacy in nursing students: Intervention study. Nurse Educator, 41(1), E1–E4. https://doi.org/10.1097/NNE.0000000000000194	Exclude
72	Kononowicz, A.A., Woodham, L.A., Edelbring, S., Stathakarou, N., Davies, D., Saxena, N., ... Zary, N. (2019). Virtual patient simulations in health professions education: Systematic review and meta-analysis by the digital health education collaboration. Journal of Medical Internet Research, 21(7), e14676. https://doi.org/10.2196/14676	Exclude
73	Korayem, G.B., & Alboghdadly, A.M. (2020). Integrating simulation into advanced pharmacy practice experience curriculum: An innovative approach to training. Saudi Pharmaceutical Journal, 28(7), 837–843. https://doi.org/10.1016/j.jsps.2020.06.004	**Include**
74	Kubin L, Fogg N, Trinka M. Transitioning child health clinical content from direct care to online instruction. The Journal of Nursing Education 2021;60(3):177-179.	Exclude
75	Lapkin, S., Levett-Jones, T., Bellchambers, H., Fernandez, R., 2010. Effectiveness of patient simulation manikins in teaching clinical reasoning skills to undergraduate nursing students: a systematic review. Clin. Simul. Nurs. 6, e207–e222	Exclude
76	Larue, C., Pepin, J., Allard, Ã., 2015. Simulation in preparation or substitution for clinical placement: a systematic review of the literature. J. Nurs. Educ. Pract. 5, 132	Already included
77	Lee K, Baird M, Lewis S, McInerney J, Dimmock M. Computed tomography learning via high-fidelity simulation for undergraduate radiography students. Radiography (Lond) 2020;26(1):49–56.	Exclude
78	Lee, J., Oh, P.J., 2015. Effects of the use of high-fidelity human simulation in nursing education: a meta-analysis. J. Nurs. Educ. 54, 501–507.	Exclude
79	Lewis, A., Rudd, C. J., & Mills, B. (2018). Working with children with autism: An interprofessional simulation-based tutorial for speech pathology and occupational therapy students. Journal of Interprofessional Care, 32(2), 242–244. https://doi.org/10.1080/13561820.2017.1388221	Exclude
80	Lewis, R., Strachan, A., McKenzie Smith, A., 2012. Is high fidelity simulation the most effective method for the development of non-technical skills in nursing? A review of the current evidence. Open Nurs. J. 6, 82–89.	Exclude
81	Liley T, Ryan E, Lee K, Dimmock M, Robinson J, Lewis SJ. Student perceptions of remote access simulated learning in computed tomography. Interact Learn Environ 2018;1–11.	Exclude
82	Lucas C, Williams K, Bajorek B. Virtual pharmacy programs to prepare pharmacy students for community and hospital placements. Am JPharm Educ 2019;83(10):7011.	Exclude
83	Luo Y, Geng C, Pei X, et al. The evaluation of the distance learning combining webinars and virtual simulations for senior nursing students during the COVID-19 Period. Clinical Simulation in Nursing 2021;57:31-40.	Exclude
84	Mackenzie, D. E., Collins, K. E., Guimond, M. J., Hunter, A. C., Jurcina, K. J., Mcdonald, J. L., Mackenzie, D. E., Collins, K. E., Guimond, M. J., Hunter, A. C., Jurcina, K. J., Richards, J. L., Sinclair, N. L., & Taylor, S. H. (2018). Co-constructing Simulations with Learners: Roles, Responsibilities, and Impact. The Open Journal of Occupational Therapy, 6(1), 1. https://doi.org/10.15453/2168-6408.1335	Exclude
85	MacKenzie, D., Creaser, G., Sponagle, K., Gubitz, G., MacDougall, P., Blacquiere, D., Miller, S., & Sarty, G. (2017). Best practice interprofessional stroke care collaboration and simulation: The student perspective. Journal of Interprofessional Care, 31(6), 793–796. https://doi.org/10.1080/13561820.2017.1356272	Exclude

86	Martin-Delgado L, Goni-Fuste B, Monforte-Royo C, et al. A teaching role practicum during the COVID-19 for final year nursing students in Spain: A qualitative study. Journal of Professional Nursing 2022;42:51-57.	Exclude
87	McGaghie, W.C., Issenberg, S.B., Cohen, E.R., Barsuk, J.H., & Wayne, D.B. (2011). Does simulation-based medical education with deliberate practice yield better results than traditional clinical education? A meta-analytic comparative review of the evidence. Academic Medicine, 86(6), 706–711. https://doi.org/10.1097/ACM.0b013e318217e119	Exclude
88	Messer S, Griffiths M. An online clinical governance learning package for student radiographers. Radiography 2007;13(2):95–102.	Exclude
89	Meyer, M. N., Connors, H., Hou, Q., & Gajewski, B. (2011). The effect of simulation on clinical performance: A junior nursing student clinical comparison study. Simulation in Healthcare, 6(5), 269–277	**Include**
90	Mileder, L.P., Bereiter, M., & Wegscheider, T. (2021). Telesimulation as a modality for neonatal resuscitation training. Medical Education Online, 26(1), 1892017. https://doi.org/10.1080/10872981.2021.1892017	Exclude
91	Miles, A., Friary, P., Jackson, B., Sekula, J., & Braakhuis, A. (2016). Simulation-based dysphagia training: Teaching interprofessional clinical reasoning in a hospital environment. Dysphagia, 31(3), 407–415. https://doi.org/10.1007/s00455-016-9691-0	Exclude
92	Miller AH, Tomlinson S, Tomlinson JD, Readinger J. Addition of a patient examination module to address student preparedness for the first full-time clinical experience. J Phys Ther Educ 2017;31(2).	Exclude
93	Mills, B., Hansen, S., Nang, C., Mcdonald, H., Lyons-, P., Hunt, J., Sullivan, T. O., Mills, B., Hansen, S., Nang, C., Mcdonald, H., Mills, B., Hansen, S., Sullivan, T. O., Nang, C., Mcdonald, H., Lyons-wall, P., & Hunt, J. (2019). A pilot evaluation of simulation-based interprofessional education for occupational therapy, speech pathology and dietetic students: Improvements in attitudes and confidence confidence. Journal of Interprofessional Care, 00(00), 1–9. https://doi.org/10.1080/13561820.2019.1659759	Exclude
94	Nguyen W, Fromer I, Remskar M, et al. Development and Implementation of Video500 Recorded Simulation Scenarios to Facilitate Case-Based Learning Discussions for Medical Students' Virtual Anesthesiology Clerkship. MedEdPORTAL 2023;4(19):e11306. doi: 10.15766/mep_2374-8265.11306.	**Include**
95	Norman, J., 2012. Systematic review of the literature on simulation in nursing education. ABNF J. 23, 24–28	Exclude
96	Offiah, G., Ekpotu, L.P., Murphy, S., Kane, D., Gordon, A., O'Sullivan, M., ... Condron, C.M. (2019). Evaluation of medical student retention of clinical skills following simulation training. BMC Medical Education, 19(1), 263. https://doi.org/10.1186/s12909-019-1663-2	Exclude
97	Oh, P.-J., Jeon, K.D., Koh, M.S., 2015. The effects of simulation-based learning using standardized patients in nursing students: a meta-analysis. Nurse Educ. Today 35: e6–e15. http://dx.doi.org/10.1016/j.nedt.2015.1001.1019	Exclude
98	Opie C, Elsner K. Using simulations to train students in treatment planning. Radiat Ther 2010;19(1):18–26.	Exclude
99	Ozelie, R., Both, C., Fricke, E., & Maddock, C. (2016). High-fidelity simulation in occupational therapy curriculum: Impact on level II fieldwork performance. The Open Journal of Occupational Therapy, 4(4). https://doi.org/10.15453/2168-6408.1242	Exclude
100	Parker, R. A., McNeill, J., & Howard, J. (2015). Comparing pediatric simulation and traditional clinical experience: Student perceptions, learning outcomes, and lessons for faculty. Clinical Simulation in Nursing, 11(3), 188-193. https://doi.org/10.1016/j.ecns.2015.01.002.	**Include**

101	PfaffMA. Learning together: the image gently interprofessional simulation for nursing and allied health students. Teach Learn Nurs 2014; 9(3):108–114.	Exclude
102	Phillips ES, Wood GJ, Yoo J, et al. A virtual field practicum: building core competencies prior to agency placement. JSoc Work Educ 2018;54(4): 620–640.	Exclude
103	Pitout, H., Human, A., Treadwell, I., & Sobantu, N. A. (2016). Healthcare students' perceptions of a simulated interprofessional consultation in an outpatient clinic. Innovations in Education and Teaching International, 53(3), 338–348. https://doi.org/10.1080/14703297.2014.993417	Exclude
104	Quail M, Brundage SB, Spitalnick J, Allen PJ, Beilby J. Student self- reported communication skills, knowledge and confidence across standardised patient, virtual and traditional clinical learning environments. BMCMed Educ 2016;16(1):73.	**Include**
105	Rahm A, Tollner M, Hubert M, et al. Effects of realistic e-learning cases on students' learning motivation during COVID-19. PloS one 2021;16(4):e0249425. doi:10.1371/journal.pone.0249425.	Exclude
106	Redinger K, Greene J. Virtual emergency medicine clerkship curriculum during the COVID-19 pandemic: Development, application, and outcomes. Western Journal of Emergency Medicine: Integrating Emergency Care with Population Health 2021;22(3):792-798.	**Include**
107	Reed HE. An Examination of Critical Thinking Skills in Traditional and Simulated Environments for Occupational Therapy Students [Ed.D.].Ann Arbor, MI: San Diego State University; 2014.	**Include**
108	Reichl, K., Baird, J. M., Chisholm, D., & Terhorst, L. (2019). Measuring and describing occupational therapists' perceptions of the impact of high-fidelity, high-technology simulation experiences on performance. American Journal of Occupational Therapy, 73(6),7306205090p1. https://doi.org/10.5014/ajot.2019.034694	Exclude
109	Ricketts, B., Clair, M., Louise, S., 2013. Simulated practice learning in a preregistration programme. Br. J. Nurs. 21, 435–440.	Excluded at full text in covidence
110	Roberts, E., Kaak, V., & Rolley, J. (2019). Simulation to replace clinical hours in nursing: A meta-narrative review. Clinical Simulation in Nursing, 37, 5–13. https://doi.org/10.1016/j.ecns.2019.07.003	Already included
111	Ross, J.G., 2012. Simulation and psychomotor skill acquisition: a review of the literature. Clin. Simul. Nurs. 8, e429–e435.	Exclude
112	Sabus, C., Sabata, D., & Antonacci, D. (2011). Use of a virtual environment to facilitate instruction of an interprofessional home assessment. Journal of Allied Health, 40(4), 199–205.	Exclude
113	Samueli B, Sror N, Jotkowitza A, et al. Remote pathology education during the COVID511 19 era: Crisis converted to opportunity. Annals of Diagnostic Pathology 2020;49:151612. doi: 10.1016/j.anndiagpath.2020.151612.	Exclude
114	Sando KR, Elliott J, StantonML, DotyR. An educational tool for teaching medication history taking to pharmacy students. Am J Pharm Educ 2013; 77(5):105.	Exclude
115	Schlairet, M. C., & Pollock, J. (2010). Equivalence testing of traditional and simulated clinical experiences: Undergraduate nursing students' knowledge acquisition. Journal of Nursing Education, 49(1), 43–47. https://doi.org/10.3928/01484834-20090918-08	Already included
116	Shea, C.-K. (2015). High-fidelity simulation: A tool for occupational therapy education. The Open Journal of Occupational Therapy, 3. https://doi.org/10.15453/2168-6408.1155	Exclude
117	Shearer, J.E., 2013. High-fidelity simulation and safety: an integrative review. J. Nurs. Educ. 52 (1):39–45. http://dx.doi.org/10.3928/01484834-20121121-01.	Exclude
118	Shiner N. Can simulation impact on first year diagnostic radiography students' emotional preparedness to encounter open wounds on their first clinical placement: a pilot study. Radiography 2019;25(4):294–300.	Exclude

119	Shoemaker, M. J., Beasley, J., Cooper, M., Perkins, R., Smith, J., & Swank, C. (2011). A method for providing high-volume interprofessional simulation encounters in physical and occupational therapy education programs. Journal of Allied Health, 40(1), e15–e21. https://www.ingentaconnect.com/content/asahp/jah/2011/00000040/00000001/art00012#	Exclude
120	Shoemaker, M. J., Platko, C. M., Cleghorn, S. M., & Booth, A. (2014). Virtual patient care: An interprofessional education approach for physician assistant, physical therapy and occupational therapy students. Journal of Interprofessional Care, 28(4), 365–367. https://doi.org/10.3109/13561820.2014.891978	Exclude
121	Shorey, S., & Ng, E.D. (2021). The use of virtual reality simulation among nursing students and registered nurses: A systematic review. Nurse Education Today, 98, 104662. https://doi.org/10.1016/j.nedt.2020.104662	Exclude
122	Shorland J, Morris C, Stephens D. Simulation speaks for itself: building speechlanguage pathology students' confidence through high quality simulation within a workplace clinical placement. Focus Health ProfEduc 2018;2:53.	Excluded at full text in covidence
123	Shrestha, R., Badyal, D., Shrestha, A.P., & Shrestha, A. (2020). In-situ simulation-based module to train interns in resuscitation skills during cardiac arrest. Advances in Medical Education and Practice, 2011(11), 271–285. https://doi.org/10.2147/AMEP.S246920	Exclude
124	Silberman NJ, Litwin B, Panzarella KJ, Fernandez-Fernandez A. High fidelity human simulation improves physical therapist student self-efficacy for acute care clinical practice. J Phys Ther Educ 2016;30(1):14–24.	Exclude
125	Silberman NJ, Litwin B, Panzarella KJ, Fernandez-Fernandez A. Student clinical performance in acute care enhanced through simulation training. J Acute Care Phys Ther 2016;7(1):25–36.	Exclude
126	Silberman NJ, Panzarella KJ, Melzer BA. Using human simulation to prepare physical therapy students for acute care clinical practice. JAllied Health 2013;42(1):25–32.	Exclude
127	Skrable, L., Fitzsimons, V., 2014. Simulation in associate degree nursing education: a literature review. Teach. Learn. Nurs. 9, 120–125.	Exclude
128	Smith J, Jones P. The COVID-19 e-lective: using innovation to manage disrupted medical student clinical placements. BMC Medical Education 2023;623(1):92. doi: 10.1186/s12909-023-04067-w.	**Include**
129	Soccio, D. A. (2017). Effectiveness of mental health simulation in replacing traditional clinical hours in baccalaureate nursing education. Journal of Psychosocial Nursing and Mental Health Services, 55, 36-43. https://doi.org/10.3928/02793695-20170905-03.	Already included
130	Springfield, E., Honnery, M., & Bennett, S. (2018). Evaluation of a simulation clinic for improving occupational therapy students' perceptions of interaction with parents and infants. British Journal of Occupational Therapy, 81(1), 51–58. https://doi.org/10.1177/0308022617736504.	Exclude
131	Steehler A, Pettitt-Schieber B, Studer M, et al. Implementation and evaluation of a virtual elective in otolaryngology in the time of COVID-19. Otolaryngology Head Neck Surgery Journal 2020;164(3):556–561.	Exclude
132	Stroup, C., 2014. Simulation usage in nursing fundamentals: integrative literature review. Clin. Simul. Nurs. 10, e155–e164.	Exclude
133	Sun, Y., Pan, C., Li, T., & Gan, T.J. (2017). Airway management education: Simulation based training versus non-simulation based training – A systematic review and meta-analyses. BMC Anesthesiology, 17(1), 17. https://doi.org/10.1186/s12871-017-0313-7	Exclude
134	Taylor N, Wyres M, Green A, et al. Developing and piloting a simulated placement experience for students. Brtitish Journal of Nursing 2021;30(13), S19–S24. doi: 10.12968/bjon.2021.30.13.S19.	Already included
135	Taylor S, Fatima Y, Lakshman N, Roberts H. Simulated interprofessional learning activities for rural health care services: perceptions ofhealth care students. JMultidiscip Healthc 2017;10:235–241.	Exclude

136	Teles, M.G., Mendes-Castillo, A.M.C., De Souza Oliveira-Kumakura, A.R., & Silva, J.L.G. (2020). Clinical simulation in teaching pediatric nursing: Students' perception. Revista Brasileira de Enfermagem, 73(2), e20180720. https://doi.org/10.1590/0034-7167-2018-0720	Exclude
137	They included this in their table but didn't reference it: Wu, R., & Shea, C. (2020). Examining Occupational Therapy Students' Responses to Integrative Seminars. The Open Journal of Occupational Therapy, 8(2), 1-11. https://doi.org/10.15453/2168-6408.1684	Exclude
138	Thomas, E. M., Rybski, M. F., Apke, T. L., Kegelmeyer, D. A., & Kloos, A. D. (2017). An acute interprofessional simulation experience for occupational and physical therapy students: Key findings from a survey study. Journal of Interprofessional Care, 31(3), 317–324. https://doi.org/10.1080/13561820.2017.1280006	Exclude
139	Treadwell, I., & Havenga, H. S. (2013). Ten key elements for implementing interprofessional learning in clinical simulations. African Journal of Health Professions Education, 5(2), 80. https://doi.org/10.7196/ajhpe.233	Exclude
140	Turner RE, Evers WD, Wood OB, Lehman JD, Peck LW. Computer- based simulations enhance clinical experience of dietetics interns. Jam Diet Assoc 2000;100(2):183–190.	Exclude
141	Tuttle N, Bialocerkowski A. Developing student skills to actively engage in feedback conversations: a pilot study. Int J Allied Health Sci Pract 2017; 15(4). DOI 10.46743/1540-580X/2017.1681	Exclude
142	Tuttle N, Horan SA. The effect of replacing 1 week of content teaching with an intensive simulation-based learning activity on physiotherapy student clinical placement performance. Adv Simul 2019;4(suppl 1):14.	Exclude
143	van Vuuren, S. (2016). Reflections on simulated learning experiences of occupational therapy students in a clinical skills unit at an institution of higher learning. South African Journal of Occupational Therapy, 46(3), 80–84. https://doi.org/10.17159/2310-3833/2016/v46n3/a13	Exclude
144	Van Wyk, R., Labuschagne, M. J., & Joubert, G. (2020). Simulation as an educational strategy to deliver interprofessional education. African Journal of Health Professions Education, 12(2), 74. https:// doi.org/10.7196/AJHPE.2020.v12i2.1213	Exclude
145	Velde, B. P., Lane, H., & Clay, M. (2009). Hands on learning: The use of simulated clients in intervention cases. Journal of Allied Health, 38(1), 17–22.	Exclude
146	Vermeulen, J., Beeckman, K., Turcksin, R., Van Winkel, L., Gucciardo, L., Laubach, M., ... Swinnen, E. (2017). The experiences of last-year student midwives with high-fidelity perinatal simulation training: A qualitative descriptive study. Women and Birth, 30(3), 253–261. https://doi.org/10.1016/j.wombi.2017.02.014	Exclude
147	Villa S, Janeway H, Preston-Suni K, et al. An Emergency medicine virtual clerkship: Made for COVID, here to stay. Western Journal of Emergency Medicine 2021;23(1):33-39.	**Include**
148	Vincent, M.A., Sheriff, S., Mellott, S., 2015. The efficacy of high-fidelity simulation on psychomotor clinical performance improvement of undergraduate nursing students. Comput. Inform. Nurs. 33:78–84. http://dx.doi.org/10.1097/CIN.0000000000000136.	Exclude
149	Vyas D, Wombwell E, Russell E, Caligiuri F. High-fidelity patient simulation series to supplement introductory pharmacy practice experiences. Am J Pharm Educ 2010;74(9):169.	Exclude
150	Walls, D. J., Fletcher, T. S., & Brown, D. P. (2019). Occupational Therapy Students. Perceived Value of Simulated Learning Experiences', Journal of Allied Health, 48(1), e21–e25.	Exclude
151	Warren, J.N., Luctkar-Flude, M., Godfrey, C., & Lukewich, J. (2016). A systematic review of the effectiveness of simulation-based education on satisfaction and learning outcomes in nurse practitioner programs. Nurse Education Today, 46, 99–108. https://doi.org/10.1016/j.nedt.2016.08.023	Exclude
152	Watchorn, V., Larkin, H., Ang, S., & Hitch, D. (2013). Strategies and effectiveness of teaching universal design in a cross-faculty setting. Teaching in Higher Education, 18(5), 477–490. https://doi.org/10.1080/13562517.2012.752730	Exclude

153	Watson, K., Wright, A., Morris, N., McMeeken, J., Rivett, D., Blackstock, F., ... Jull, G. (2012). Can simulation replace part of clinical time? Two parallel randomised controlled trials. Medical Education, 46(7), 657– 667. https://doi.org/10.1111/j.1365-2923.2012.04295.x	Already included
154	Weaver, A., 2011. High-fidelity patient simulation in nursing education: an integrative review. Nurs. Educ. Perspect. 32:37–40. http://dx.doi.org/10.5480/1536-5026-5432. 5481.5437.	Exclude
155	Weston J, Zauche L. Comparison of virtual simulation to clinical practice for prelicensure nursing students in pediatrics. Nurse Educator 2021;46(5), E95-E98. doi: 10.1097/NNE.0000000000000946.	Already included
156	White M, Birkness J, Salimian K, et al. Continuing undergraduate pathology medical education in the coronavirus disease 2019 (COVID-19) global pandemic: The Johns Hopkins virtual surgical pathology clinical elective. Archives of Pathology of Laboratory Medicine 2021;145(7):814-820.	Exclude
157	Wik V, Barfield S, Cornwall M, et al. Finding the right balance: Student perceptions of using virtual simulation as a community placement. International Journal of Nursing Education Scholarship 2022;19(1). doi: 10.1515/ijnes-2021-0135.	Already included
158	Williams B, Brown T, Scholes R, French J, Archer F. Can interdisciplinary clinical DVD simulations transform clinical fieldwork education for paramedic, occupational therapy, physiotherapy, and nursing students? J Allied Health 2010;39(1):3–10.	Exclude
159	Williams C, Familusi O, Ziemba J, et al. Adapting to the educational challenges of a pandemic: development of a novel virtual urology subinternship during the time of COVID536 19. Urology 2021;148:70–76.	Exclude
160	Williams, B., French, J., & Brown, T. (2009). Can interprofessional education DVD simulations provide an alternative method for clinical placements in nursing? Nurse Education Today, 29(6), 666-670. https://doi.org/10.1016/j.nedt.2009.02.008.	Excluded at full text in covidence
161	Wilson WJ, Hill A, Hughes J, Sher A, Laplante-Levesque A. Student audiologists' impressions of a simulation training program. Austr NZ J Audiol 2010;32(1):19–30.	Exclude
162	Wright, A., Moss, P., Dennis, D.M., Harrold, M., Levy, S., Furness, A.L., ... Reubenson, A. (2018). The influence of a full-time, immersive simulation-based clinical placement on physiotherapy student confidence during the transition to clinical practice. Advances in Simulation, 3(1), 3. https://doi.org/10.1186/s41077-018-0062-9	Already included
163	Yang, L., Li, Y., Liu, J., & Liu, Y. (2020). Effect of vascular simulation training on practice performance in residents: A retrospective cohort study. BMJ Open, 10(9), e037338. https://doi.org/10.1136/bmjopen-2020-037338	Exclude
164	Yeung, E., Dubrowski, A., & Carnahan, H. (2013). Simulationaugmented education in the rehabilitation professions: A scoping...: EBSCOhost. International Journal of Therapy and Rehabilitation. http://web.b.ebscohost.com.apollo.worc.ac.uk/ehost/pdfviewer/pdfviewer?vid=12&sid=21af7825-7e8d-417a-a3ad-fc2c7ea27529%40pdc-v-sessmgr06	Exclude
165	Yuan, H.B., Williams, B.A., Fang, J.B., 2012a. The contribution of high-fidelity simulation to nursing students' confidence and competence: a systematic review. Int. Nurs. Rev. 59, 26–33.	Exclude
166	Yuan, H.B., Williams, B.A., Fang, J.B., Ye, Q.H., 2012b. A systematic review of selected evidence on improving knowledge and skills through high-fidelity simulation. Nurse Educ. Today 32, 294–298.	Exclude
167	Zahara-Such, R.M., 2013. Improving medication calculations of nursing students through simulation: an integrative review. Comput. Inform. Nurs. 9, e379–e383.	Exclude

168	Zamjahn, J. B., Beyer, E. O., Alig, K. L., Mercante, D. E., Carter, K. L., & Gunaldo, T. P. (2018). Increasing awareness of the roles, knowledge, and skills of respiratory therapists through an interprofessional education experience. Respiratory Care, 63(5), 510. https://doi.org/10.4187/respcare.05869	Exclude
169	Zarifsanaiey, N., Amini, M., & Saadat, F. (2016). A comparison of educational strategies for the acquisition of nursing student's performance and critical thinking: Simulation-based training vs. integrated training (simulation and critical thinking strategies). BMC Medical Education, 16(1), 294. https://doi.org/10.1186/s12909-016-0812-0	Exclude
170	Zhang, M., Cheng, X., Xu, A., Luo, L., & Yang, X. (2015). Clinical simulation training improves the clinical performance of Chinese medical students. Medical Education Online, 20(1), 28796. https://doi.org/10.3402/meo.v20.28796	Exclude
171	Zhou T, Huang S, Cheng J, et al. The distance teaching practice of combined mode of massive open online course micro-video for interns in emergency department during the COVID-19 epidemic period. Telemedicine Journal of e-Health 2020;26, 584–588.	Exclude

Contacting authors

Full texts received through contacting authors of relevant abstracts.

Where there was a conference abstract that met title and abstract screening criteria, we contacted the authors of these abstracts to determine if there were any associated full texts. We contacted 25 authors and received twelve responses. We sent follow up emails to the remaining authors who did not respond. An additional four author's emails could not be located.

Out of these twelve responses, seven abstracts did not have associated full texts. However, five abstracts had a total of 14 full-text reports emanating from or related to these abstracts (Yu 2016, one publication[16]; Jull 2011, two publications[17,18]; Tuttle 2015, five publications[19-23]; Bridge 2019, six publications[24-29]; McEwen 2023, two publications[30,31]). After removing duplicates and checking if these sources were already identified and screened from our original search, six papers were sent to full-text screening.[16,20,21,23,26,29] One was included in this review.[29]

List of excluded studies

Studies excluded at full text screening stage (n = 122)

Title	Authors	Published Year	Notes
Realistic Radiation Therapist Training in a Simulated Clinical Department	Bridge, P.; Al-Samarraie, F.; Ball, B.; Calder, K. A.; Callender, J.; Edgerley, J.; Gordon, C.; Ketterer, S. J.; Kirby, M.; Pagett, M.; Pilkington, P.; Porritt, B.; Warren, M.	2019	Exclusion reason: Abstract only - no full text
ELECTIVE SIMULATION for UNDERGRADUATE NURSES	Harris, K.; Hamilton, C.	2020	Exclusion reason: Abstract only - no full text
Reinventing Standard Patient Experience During COVID: A Sustainable Approach...Association of Schools Advancing Health Professions (ASAHP) Annual Conference October 20-21, 2021, The Westin Long Beach, Long Beach, California	Majeski, Karen; VanOss, Tracy; Ceccolini, Gabbriel	2022	Exclusion reason: Abstract only - no full text
Role-play simulation in Australian entry-level physiotherapy clinical education: A qualitative evaluation involving students, staff, actors and administrators	Moss, P.; Wright, A.; Watson, K.; Rue, S.	2015	Exclusion reason: Abstract only - no full text

A Multi-Layer Model of Clinical Skill Training Using Virtual Simulation Technology...Association of Schools Advancing Health Professions, Live Virtual Series, September 30, 2020 - March 24, 2021	Neubauer, Natalie; Dayalu, Vikram; Grabowski, Caryn	2021	Exclusion reason: Abstract only - no full text
THE USE OF SIMULATION TO SUPPLEMENT PRACTICUM TIME...Canadian Society of Respiratory Therapists Annual Conference, May 11-13, 2023, Prince Edward Island, Canada	Witschen, Brian	2023	Exclusion reason: Abstract only - no full text
Using Virtual Clinical Placement to Enhance Student Learning and Readiness for Practice	McEwen, A.; Berkman, J.; Jacobs, C.	2023	Exclusion reason: Abstract only - no full text
COMPARISON OF SRT PEDIATRIC EDUCATION: STUDY DESIGN...Proceedings from the Canadian Society of Respiratory Therapists Annual Education Conference May 11 -13, 2017 • Halifax, Nova Scotia	Reise, K.; Correia, R	2017	Exclusion reason: Abstract only - no full text
Should DVD simulations replace hospital-based clinical placements?	Williams, B.; Brown, T.	2007	Exclusion reason: Abstract only - no full text
Implementation of a new teaching program including medical simulation for undergraduate medical students in intensive care unit (ICU): a case-control study	Martino, F.; Petchy, M. F.; Piednoir, P.; Eugene, V.; Thomar, P.; Ardisson, F.; Valette, M.	2023	Exclusion reason: Abstract only - no full text
Clinical Placements in Pharmacy: Development of an on-line learning module to orientate students to clinical practice	Bajorek, B.; Koulias, M.; Lobastov, M.; Woulfe, J.; Ryan, G.	2010	Exclusion reason: Abstract only - no full text
Can simulated learning environments successfully augment traditional clinical immersion in the education of physiotherapy students? A randomised controlled trial	Jull, G.; Watson, K.; Blackstock, F.; Morris, N.; Wright, A.; Rivett, D.; McMeeken, J.; Jones, A.; Haines, T.; O'Connor, V.; Peterson, R.	2011	Exclusion reason: Abstract only - no full text
Comparison of SRT pediatric education: Study design	Reise, K.; Correia, R.	2017	Exclusion reason: Abstract only - no full text
Increasing external clinical placement capacity and supplementing placement content through a centrally located telemedicine simulated learning environment	Tuttle, N.; Waite, M.; Bialocerkowksi, A.	2015	Exclusion reason: Abstract only - no full text
Student Perceptions of a Hip-Hop Based Community Models Level IFieldwork for Level II Fieldwork Preparedness: A Case Study...National Society of Allied Health Annual Conference and Meeting, March 10-12, 2022, Tallahassee, Florida	Jones, J.; Alexander, A.; Etheridge, J.	2022	Exclusion reason: Abstract only - no full text
A Simulated Hospital in a COVID-19 Pandemic Environment for Undergraduate Neurology Students	Toro, J.; Rivera, J. S.; Rodriguez, D.; Serna, L. A.; Gaitan, J.; Medina, T.; Cortés-Muñoz, F.; Yepes, M.	2022	Exclusion reason: Abstract only - no full text
Implementing a virtual placement scheme for physiotherapy students in the UK: The experiences of clinical educators...Physiotherapy UK Virtual Conference, November 5-6, 2021	Miller, P. K.; Godley, S.; Smith, S.; Twogood, R.	2022	Exclusion reason: Abstract only - no full text
The development and initial evaluation of a simulated clinical radiotherapy training centre	Ketterer, S.; Bridge, P.	2019	Exclusion reason: Abstract only - no full text
The importance of 'core' clinical placements upon graduate self-perceived confidence and effectiveness in the healthcare workplace	Wyres, M.; Toepritz, A.	2021	Exclusion reason: Abstract only - no full text
Adapting undergraduate teaching in the COVID 19 era	Armstrong, L.; O'Brien, S.; Weir, C.	2021	Exclusion reason: Abstract only - no full text
A profession-wide collaboration to embed role-play simulation into Australian entry-level physiotherapy clinical training	Wright, A.; Moss, P.; Watson, K.; Rue, S.; Jull, G.; Mandrusiak, A.; Reubenson, A.; Connaughton, J.; Redmond, C.; MacIntosh, S.; Alison, J.; Chipchase, L.; Clements, T.; Blackstock, F.; Morgan, P.; Laakso, L.; Van Der Zwan, K.; Corrigan, R.; Jones, A.; Teys, P.; Palmer, T.	2015	Exclusion reason: Abstract only - no full text

The evaluation of a Peer Enhanced eLearning Placement (PEEP) in MSc Physiotherapy (pre-registration) education	Stears, A.; Thomas, J.; Lane, J.; Salisbury, L.; Rhodes, J.; Ackerman, L.; Baer, G.	2022	Exclusion reason: Abstract only - no full text
Effectiveness and cost-effectiveness of embedded simulation in occupational therapy clinical practice education: Study protocol for a randomised controlled trial	Imms, C.; Chu, E. M. Y.; Guinea, S.; Sheppard, L.; Froude, E.; Carter, R.; Darzins, S.; Ashby, S.; Gilbert-Hunt, S.; Gribble, N.; Nicola-Richmond, K.; Penman, M.; Gospodarevskaya, E.; Mathieu, E.; Symmons, M.	2017	Exclusion reason: Abstract only - no full text
A Blended Learning Approach to Surgical Teaching; What We Can Learn From The Student Experience	McCormack, D.; Martin-Smith, J.; Cantillon, P.	2022	Exclusion reason: Abstract only - no full text
Development of a framework for radiographer online clinical education (FORCE)	Matthews, K.; Atutornu, J.; Bezzina, P.; Costa, P.; Dalen, N.; Huhtanen, J. T.; Jaronen, M.; Kamp, C.; Rainford, L.	2022	Exclusion reason: Not a simulated clinical placement
Shortages lead to virtual placements		2009	Exclusion reason: Not a simulated clinical placement
Using standardized patients in an undergraduate mental health simulation: A pilot study	Alexandera, Louise; Dearsley, Amy	2013	Exclusion reason: Not a simulated clinical placement
The co-creation of an innovative curriculum model: Balancing lab, simulation, and clinical	Ardern, Rachel	2022	Exclusion reason: Not a simulated clinical placement
Nursing students' perception of high-fidelity simulation activity instead of clinical placement: A qualitative study	Au, Mio Leng; Lo, Man Sao; Cheong, Wan; Wang, Si Chen; Van, Iat Kio	2016	Exclusion reason: Not a simulated clinical placement
A modified systematic review of research evidence about education for pre-registration nurses in palliative care	Bassah, N.; Seymour, J.; Cox, K.	2014	Exclusion reason: Not a simulated clinical placement
Use of Simulation in Occupational Therapy Education: Way of the Future?	Bethea, Dorothy Peterson; Castillo, Dahlia Cavazos; Harvison, Neil	2014	Exclusion reason: Not a simulated clinical placement
Using Virtual Interactive Digital Simulator to Enhance Simulation Experiences for Undergraduate Nursing Students	Betts, Lorraine; Schmid, Jacqueline; Sivaramalingam, Suba; Verkuyl, Margaret	2020	Exclusion reason: Not a simulated clinical placement
Baccalaureate Nursing Students' Perceptions of Learning in Mentored and Simulated Research Practica	Bird, Marissa; Tolan, Jesseca; Carter, Nancy	2019	Exclusion reason: Not a simulated clinical placement
The use of immersive simulation in paramedicine education: A scoping review	Birtill, Michael; King, James; Jones, Donovan; Thyer, Liz; Pap, Robin; Simpson, Paul	2021	Exclusion reason: Not a simulated clinical placement
Simulation: A Day in the Life of a Pediatric Nurse	Bishop, Samantha; Stewart, Patricia	2014	Exclusion reason: Not a simulated clinical placement

Student perception and assessment outcomes using a pilot condensed format ("boot camp") in a Fixed Prosthodontics course	Bompolaki, D.; Stafford, G.	2023	Exclusion reason: Not a simulated clinical placement
Simulation: Building Skills and Improving Outcomes	Bordelon, C. J.; Dudding, K.	2020	Exclusion reason: Not a simulated clinical placement
Simulation-based trauma education for medical students: A review of literature	Borggreve, A. S.; Meijer, J. M. R.; Schreuder, H. W. R.; Ten Cate, O.	2017	Exclusion reason: Not a simulated clinical placement
Integration of high-fidelity simulation into undergraduate nursing education in Aotearoa New Zealand and Australia: An integrative literature review	Bowen-Withington, Julie; Zambas, Shelaine; Macdiarmid, Rachel; Cook, Catherine; Neville, Stephen	2020	Exclusion reason: Not a simulated clinical placement
The effect of simulation on skill performance: a need for change in pediatric nursing education	Bowling, A. M.	2015	Exclusion reason: Not a simulated clinical placement
Medical students as simulation educators: Students' experience of a 7-week simulation-based education rotation	Brazil, V.; Caughley, M.; Middleton, L.; Powell, G.; Alsaba, N.	2021	Exclusion reason: Not a simulated clinical placement
Clinical learning needs: Student nurse perceptions of the traditional clinical environment and the simulation environment	Breymier, Tonya L.	2013	Exclusion reason: Not a simulated clinical placement
Preparing Nurse Practitioner Students to Practice in Rural Primary Care	Brommelsiek, Margaret; Peterson, Jane A.	2020	Exclusion reason: Not a simulated clinical placement
Implementing simulated practice learning for nursing students	Brooks, N.; Moriarty, A.; Welyczko, N.	2010	Exclusion reason: Not a simulated clinical placement
Preparing pediatric nurses: The role of simulation-based learning	Broussard, L.; Myers, R.; Lemoine, J.	2009	Exclusion reason: Not a simulated clinical placement
Simulation in Undergraduate Mental Health Nursing Education: A Literature Review	Brown, Allana M.	2015	Exclusion reason: Not a simulated clinical placement
Reimagining nursing education through virtual reality	Brown, Tori; Hall, Susan; Schmaltz, Ryan; McBride, John; Hamilton, Justin	2023	Exclusion reason: Not a simulated clinical placement
Enhancing the Pediatric Undergraduate Nursing Curriculum Through Simulation	Bultas, Margaret W.	2011	Exclusion reason: Not a simulated clinical placement
A LONGITUDINAL, MIXED METHODS STUDY INVESTIGATING THE EFFECTIVENESS OF SIMULATION TO PREPARE MEDICAL STUDENTS FOR PROFESSIONAL PRACTICE	Carpenter, C.; Brewster, L.; Vince, G.	2019	Exclusion reason: Not a simulated clinical placement

An Innovative Simulated Research Practicum for Undergraduate Nursing Students	Carter, Nancy; Tolan, Jesseca; Bird, Marissa	2019	Exclusion reason: Not a simulated clinical placement
Variability of clinical hours in prelicensure nursing programs: Time for a reevaluation?	Cipher, Daisha J.; LeFlore, Judy L.; Urban, Regina W.; Mancini, Mary E.	2021	Exclusion reason: Not a simulated clinical placement
Experience-based learning: how a crisis solution informed fundamental change in a clinical education curriculum	Costello, M.; Cantillon, P.; Geoghegan, R.; Byrne, D.; Lowery, A.; Walsh, S. M.	2022	Exclusion reason: Not a simulated clinical placement
Innovations in virtual education for clinical and simulation learning	Cowperthwait, Amy; Graber, Jennifer; Carlsen, Allan; Cowperthwait, Megan; Mekulski, Heather	2021	Exclusion reason: Not a simulated clinical placement
Implementation of best practice of simulation design	Creed-Hall, Sharon F.	2018	Exclusion reason: Not a simulated clinical placement
A longitudinal study exploring student nurses' perceptions of the impact of a simulated clinical environment on their clinical learning experience and transfer of learning	Crowley, Maureen A.	2013	Exclusion reason: Not a simulated clinical placement
Crating virtual placements for student nurses	Donnelly, A.	2003	Exclusion reason: Not a simulated clinical placement
Novel use of existing technology to deliver remote simulation in a nursing program during a global pandemic	Drahnak, Dawn; Katrancha, Elizabeth	2022	Exclusion reason: Not a simulated clinical placement
A comprehensive, simulation-based approach to teaching clinical skills: The medical studentsâ€™ perspective	Evans, L. V.; Crimmins, A. C.; Bonz, J. W.; Gusberg, R. J.; Tsyrulnik, A.; Dziura, J. D.; Dodge, K. L.	2014	Exclusion reason: Not a simulated clinical placement
Evaluation of virtual simulation in a master's-level nurse education certificate program	Foronda, C.; Lippincott, C.; Gattamorta, K.	2014	Exclusion reason: Not a simulated clinical placement
More of a good thing?	Foster, Sam	2023	Exclusion reason: Not a simulated clinical placement
Mapping the student experience of UK-wide virtual placement initiative in physiotherapy...Physiotherapy UK Virtual Conference, November 5-6, 2021	Godley, S.; Smith, S.; Twogood, R.; Miller, P. K.	2022	Exclusion reason: Not a simulated clinical placement
Simulation-enhanced pediatric clinical orientation	Harris, M. A.	2011	Exclusion reason: Not a simulated clinical placement
Impact of clinical simulation on the clinical competence of medical students clerkship training	Hassan, A. B.; Elamin, A.; Jaradat, A. A. K.	2018	Exclusion reason: Not a simulated clinical placement

A phenomenological study of the lived experiences of faculty who participate in simulation instruction in an undergraduate nursing program	Hernandez-Acevedo, Brenda	2017	Exclusion reason: Not a simulated clinical placement
Nursing faculty integrate simulation instruction into their teaching practice: A phenomenological study	Hernandez-Acevedo, Brenda	2021	Exclusion reason: Not a simulated clinical placement
Assessment of student competency in a simulated speech-language pathology clinical placement	Hill, Anne E.; Davidson, Bronwyn J.; McAllister, Sue; Wright, Judith; Theodoros, Deborah G.	2014	Exclusion reason: Not a simulated clinical placement
Undergraduate otolaryngology: enhancing interest and education with hands-on simulation	Hirniak, J.; Jain, A.; Van, M.; Kokotkin, I.; Vaghela, M.; Churchill, T.; Zargaran, A.	2021	Exclusion reason: Not a simulated clinical placement
Blended learning: Maintaining clinical placements for child nursing students during the COVID-19 pandemic	Ingleson, Hayley; Hunter, Amy	2022	Exclusion reason: Not a simulated clinical placement
Factors in the clinical learning environment that influence caring behaviors of undergraduate nursing students: An integrative review	Inocian, Ergie P.; Hill, Meredith B.; Felicilda-Reynaldo, Rhea Faye D.; Kelly, Susan H.; Paragas, Emmanuel D.; Turk, Melanie T.	2022	Exclusion reason: Not a simulated clinical placement
A framework for designing, implementing, and evaluating: simulations used as teaching strategies in nursing	Jeffries, P. R.	2005	Exclusion reason: Not a simulated clinical placement
Delivering introductory physiotherapy clinical placements incorporating simulated learning experiences in rural settings	Johnston, Catherine; Wakely, Luke	2021	Exclusion reason: Not a simulated clinical placement
A 1-week simulated internship course helps prepare medical students for transition to residency	Laack, T. A.; Newman, J. S.; Goyal, D. G.; Torsher, L. C.	2010	Exclusion reason: Not a simulated clinical placement
Online simulated clinical experiences in mental health nursing practice	Lane, Annette; Corcoran, Lynn; Weare, Adrienne; Perr, Beth	2017	Exclusion reason: Not a simulated clinical placement
Student Nurse Experiences of Learning from Clinical Practice and Simulations in New Zealand: A Descriptive Qualitative Study	LesÄ�, Raewyn Phyllis; Daniel, Ben Kei; Harland, Tony	2022	Exclusion reason: Not a simulated clinical placement
The development and psychometric testing of the Satisfaction with Simulation Experience Scale	Levett-Jones, Tracy; McCoy, Michelle; Lapkin, Samuel; Noble, Danielle; Hoffman, Kerry; Dempsey, Jennifer; Arthur, Carol; Roche, Jan	2011	Exclusion reason: Not a simulated clinical placement
Description and student self-evaluation of a pilot integrated small group learning and simulation programme for medical students in the first clinical year	Levinson, M.; Kelly, D.; Zahariou, K.; Johnson, M.; Jackman, C.; Mackenzie, S.	2017	Exclusion reason: Not a simulated clinical placement
Virtual Pharmacy Programs to Prepare Pharmacy Students for Community and Hospital Placements	Lucas, C.; Williams, K.; Bajorek, B.	2019	Exclusion reason: Not a simulated clinical placement

Simulated learning environments in speech-language pathology: An Australian response	MacBean, Naomi; Theodoros, Deborah; Davidson, Bronwyn; Hill, Anne E.	2013	Exclusion reason: Not a simulated clinical placement
Simulation Use in Paramedic Education Research (SUPER): A Descriptive Study	McKenna, Kim D.; Carhart, Elliot; Bercher, Daniel; Spain, Andrew; Todaro, John; Freel, Joann	2015	Exclusion reason: Not a simulated clinical placement
A simulated night shift for undergraduate paramedicine students: Lessons learnt and the perceived value towards learning	Miles, Alecka; Hansen, Sara; Mills, Brennen	2020	Exclusion reason: Not a simulated clinical placement
Quantification of opportunities for early-stage paramedicine students to practice clinical skills during clinical placements compared with an equal dose of simulation-based workshops	Mills, B. W.; Carter, O. B. J.; Rudd, C. J.; Mills, J. K.; Ross, N. P.; Ruck, J. D.	2015	Exclusion reason: Not a simulated clinical placement
A Registrar - Delivered Urology Experience Improves Medical Student Knowledge of the Subject and Increases Interest in a Surgical Career	Nelson, A.; McCann, K.	2022	Exclusion reason: Not a simulated clinical placement
Exploring the Effect of COVID-19 on Graduate Nursing Education	O'Keefe, R.; Auffermann, K.	2022	Exclusion reason: Not a simulated clinical placement
A Learning Package for Medical Students in a Busy Urology Department: Design, Implementation, and Evaluation	Owen, L. E.; Byrne, D. J.; Ker, J. S.	2008	Exclusion reason: Not a simulated clinical placement
Integration of Technology in Medical Education on Primary Care During the COVID-19 Pandemic: Students' Viewpoint	Paul, N.; Kohara, S.; Khera, G. K.; Gunawardena, R.	2020	Exclusion reason: Not a simulated clinical placement
Comparison of Quality and Safety Education for Nurses (QSEN)-Related Student Experiences During Pediatric Clinical and Simulation Rotations	Pauly-O'Neill, Susan; Prion, Susan; Nguyen, Helen	2013	Exclusion reason: Not a simulated clinical placement
The Virtual Maternity Clinic: A teaching and learning innovation for midwifery education	Phillips, Diane; Duke, Maxine; Nagle, Cate; Macfarlane, Susie; Karantzas, Gery; Patterson, Denise	2013	Exclusion reason: Not a simulated clinical placement
Simulated practice learning in a preregistration programme	Ricketts, Barry; Merriman, Clair; Stayt, Louise	2012	Exclusion reason: Not a simulated clinical placement
Virtual patient clinical placements improve student communication competence	Robinson, Kate E.; Allen, Peter J.; Quail, Michelle; Beilby, Janet	2020	Exclusion reason: Not a simulated clinical placement
A lowâ€cost ward team simulation method	Rose, Michael	2023	Exclusion reason: Not a simulated clinical placement
Creation of a Virtual Health System for Leadership Clinical Experiences	Ross, Amy Miner; Crusoe, Kristen L.	2014	Exclusion reason: Not a simulated clinical placement

An evaluation of clinical placement requirements in pharmacy and the role of the Australian Pharmacy Council in monitoring quality	Rowett, D.; Chapman, C.; Cooper, G.; Kirsa, S.; Mitchell, C.; Woolcock, K.; Adkins, R.	2013	Exclusion reason: Not a simulated clinical placement
An Audit of the Use of Simulation in Australian and New Zealand Physiotherapy Curricula	Ryall, Tayne; Preston, Elisabeth; Mahendran, Niruthikha; Bissett, Bernie	2021	Exclusion reason: Not a simulated clinical placement
Cocreation With Prelicensure Nursing Students of a Simulated Practice Placement	Salje, Joelle	2024	Exclusion reason: Not a simulated clinical placement
Student pharmacists' perspective on actual vs. simulated pharmacy practice experiences	Sansom, V. E.; Cox, E. A.	2013	Exclusion reason: Not a simulated clinical placement
Second LifeÂ® Virtual Learning in Public Health Nursing	Schaffer, Marjorie A.	2016	Exclusion reason: Not a simulated clinical placement
Faculty's Perception on Student Performance using vSim for NursingÂ® as a Teaching Strategy	Sharoff, Leighsa	2022	Exclusion reason: Not a simulated clinical placement
The Virtual Hospital, a European project in nursing education]	Sigaud, Marie-Alice; Capone, Tonino; Dupont, Marie-JosÃ©e; Grassi, Anne-Marie; Mellier, Annie; Poirier, Sylviane; Jean, JÃ©rÃ´me	2011	Exclusion reason: Not a simulated clinical placement
Rapid implementation and improvement of a virtual student placement model in response to the COVID-19 pandemic	Twogood, R.; Hares, E.; Wyatt, M.; Cuff, A.	2020	Exclusion reason: Not a simulated clinical placement
Reflections on simulated learning experiences of occupational therapy students in a clinical skills unit at an institution of higher learning	van Vuuren, Santie	2016	Exclusion reason: Not a simulated clinical placement
Controlled Postpartumâ€"Newborn Simulation With Objective Evaluation Exchanged for Clinical Learning	Veltri, Linda; Kaakinen, Joanna R.; Shillam, Casey; Arwood, Ellyn; Bell, Kathleen	2016	Exclusion reason: Not a simulated clinical placement
Virtual simulation in healthcare education: a multi-professional, pan-Canadian evaluation	Verkuyl, M.; Violato, E.; Harder, N.; Southam, T.; Lavoie-Tremblay, M.; Goldsworthy, S.; Ellis, W.; Campbell, S. H.; Atack, L.	2024	Exclusion reason: Not a simulated clinical placement
Online Virtual Nursing Placements: A Case Study on Placement Expansion	Wagg, Amanda J.; Morgan, Kate	2022	Exclusion reason: Not a simulated clinical placement
The Impact of COVID 19 on Nursing Student Clinical Practice: A Time for Clinical Innovation...Maryland Nurses Association's 117th Annual Convention (Virtual), October 1-2, 2020	Watties-Daniels, Denyce	2020	Exclusion reason: Not a simulated clinical placement
How John Barleycorn Spun Simulation Gold	Wheeler, Jana	2019	Exclusion reason: Not a simulated clinical placement

Can interprofessional education DVD simulations provide an alternative method for clinical placements in nursing?	Williams, B.; French, J.; Brown, T.	2009	Exclusion reason: Not a simulated clinical placement
Innovation in Clinical Course Delivery and Impact on Studentsâ€™ Clinical Decision-Making and Competence	Woda, Aimee; Schnable, Theresa; Alt-Gehrman, Penny; Bratt, Marilyn Meyer; Garnier-Villarreal, Mauricio	2019	Exclusion reason: Not a simulated clinical placement
A regional resident readiness course to facilitate the transition from student to doctor	Yu, H.; Evans, K.; Hampton, B.; Baecher-Lind, L.; Everett, E.	2016	Exclusion reason: Not a simulated clinical placement
Nursing Students' Experiences of Virtual and Hybrid Simulation in Gerontological Nursing: A Mixed-Methods Study	Lee H, Cho M, Choi E	2022	Exclusion reason: Not a simulated clinical placement
Evaluation of affective learning in a gamified pharmacy simulatio	Hope, D. L.; Rogers, G. D.; Grant, G. D.; King, M. A.	2023	Exclusion reason: Not a simulated clinical placement
Simulation speaks for itself: Building speech-language pathology students' confidence through high quality simulation within a workplace clinical placement	Shorland, Joanna; Morris, Clare; Stephens, Delwyne	2018	Exclusion reason: Not a simulated clinical placement
The Use of an Online Adaptive Learning Platform as an Adjunct to Live Simulated Clinical Encounters	Tuttle, Neil	2018	Exclusion reason: Not a simulated clinical placement
Developing Student Skills to Actively Engage in Feedback Conversations: A Pilot Study	Tuttle N, Bialocerkowski A.	2017	Exclusion reason: Not a simulated clinical placement
Feasibility of a Regional Approach to an Obstetrics and Gynecology Transition to Residency Course	Yu, H. Y.; Everett, E. N.; Baecher-Lind, L.; Hampton, B. S.	2019	Exclusion reason: Not a simulated clinical placement
The use of virtual reality simulation to improve technical skill in the undergraduate medical imaging student	Gunn, Therese; Jones, Lee; Bridge, Pete; Rowntree, Pam; Nissen, Lisa	2018	Exclusion reason: Not a simulated clinical placement
SHOULD SIMULATION COUNT TOWARD NURSE PRACTITIONER STUDENT CLINICAL PRACTICE HOURS?		2014	Exclusion reason: Not a simulated clinical placement
An Innovative Approach to Staffing a Simulation Center in a College of Health Professions	Berro, Elizabeth A.; Knoesel, Joanne M.	2016	Exclusion reason: Not a simulated clinical placement
Simulated learning for generic communication competency development: A case study of Australian post-graduate pharmacy students	Barker, Michelle; Fejzic, Jasmina; Mak, Anita S.	2018	Exclusion reason: Wrong patient population
A novel three-tiered approach to improving paediatric education for general practice doctors and trainees	Chase, C.; Edelman, J.; Barid, J.; Brown, N.; Farnon, T.; Sykes, K.; Pryde, K.	2015	Exclusion reason: Wrong patient population
State of research on simulation in nursing education programs	Fey, Mary K.; Kardong-Edgren, Suzan	2017	Exclusion reason: Wrong

			patient population
3D Simulation of a Hospital Environment and Ward Round to Augment a Summer School Program for Pre-Medical Students	Kulendran, M.; Taylor, M.; Taylor, D.; Darzi, A.	2014	Exclusion reason: Wrong patient population
Simulated 'skills labs' to ease pressure on training	Duffin, C.	2004	Exclusion reason: Wrong study design

Queried articles

There have been 18 queried articles that were originally included regarding their eligibility criteria. Two lead authors have completed an assessment of these and excluded the articles that should no longer be included.

Exclude: 11

Title	Query	Final decision
A review of standardized patients in clinical education: implications for speech-language pathology programs' by Hill 2010	Placement mentioned in introduction only.	EXCLUDE – Not a simulated clinical placement
EXAMINING PERCEPTIONS OF CLINICAL COMPETENCE OF UNDERGRADUATE NURSING STUDENTS DURING THE ERA OF SOCIAL DISTANCING - Foster 2021	It’s not explicitly stated that it replaces hours but is alluded too. Students were still able to attend traditional clinical placement - this is more of a supplement to learning than replacement of clin practical hours.	EXCLUDE – Not a simulated clinical placement
Christopher et al (2021) Interprofessional Healthcare Student Perceptions of Clinical vs. Simulation Learning Through Participation in Underserved Health Clinics	Exclude: comparing the clinical vs simulations but does not state that simulation is replacing clinical hours.	EXCLUDE – Not a simulated clinical placement
Perceived Effectiveness of Nursing Faculty of Clinical Video Simulation for Use in Nurse Practitioner Education	Queried - not placement replacement	EXCLUDE – Not a simulated clinical placement
"The Evidence in Simulation-Based Learning Experiences in Nursing Education and Practice: An Umbrella Review" by Mary Ann Cantrell.	Queried - has included one important review that we should actually include for our review, but other than that has not gone deeper into it.	EXCLUDE – Not a simulated clinical placement
Grant 2022 “I left feeling different about myself” – What students learn on their first practice placement.	Queried. Not about simulation - only mentions simulation as potential future research	EXCLUDE – Not a simulated clinical placement
Singh 2018 "Simulated learning environments to prepare for clinical placements: transition to placement (T2P) -	Queried. Discusses sim for preparing for a placement, not replacing a placement. Book chapter	EXCLUDE – Not a simulated clinical placement
Kulcon 2023 Identifying best practices for virtual nursing clinical education: A scoping review	Queried - this is discussing placement replacements, but not necessarily directly	EXCLUDE – Not a simulated clinical placement
Oermann 2015 Clinical simulations in nursing education: overview, essentials, and the evidence	Queried. Not placement specific, just overview of different simulation modalities	EXCLUDE – Not a simulated clinical placement
Virtual Wards: A Rapid Adaptation to Clinical Attachments in MBChB During the COVID-19 Pandemic	Book chapter. Although this has a lot of information	EXCLUDE – Wrong study design

Emerging Technologies and Work-Integrated Learning Experiences in Allied Health Education	Queried book chapter	EXCLUDE – Wrong study design

All included studies

1. USE OF SIMULATION IN RN AND PN NURSING EDUCATION PROGRAMS. Momentum (Ohio Board of Nursing). 2019; 17(1):22-4.
2. Anggraini S, Chrisnawati C, Warjiman W. The Effectiveness of the Implementation of the Hospital Clinical Practice Based Simulation Model on the Practice Learning Outcomes of Nurse Profession Students During the Covid-19. Indonesian Nursing Journal of Education & Clinic (INJEC). 2021; 6(2):185-91.
3. Baillie L, Curzio J. Students' and facilitators' perceptions of simulation in practice learning. Nurse Educ Pract. 2009; 9(5):297-306.
4. Bashir R, Kasim Y, Ward Philip T. Do we know enough? The need to research more about simulation placements in higher education institutions in order to understand its value from the perspectives of staff members and students. Journal of Clinical Nursing (John Wiley & Sons, Inc). 2022; 31(15/16):e23-e5.
5. Blackford J, Alison J, McAllister L, Nisbet G. Replacing Physiotherapy Student Clinical Education Time with Simulation - The Clinical Educators’ Experience. International Journal of Practice-based Learning in Health and Social Care. 2020; 8(1):60-72.
6. Blackford J, McAllister L, Alison JA. Simulated Learning in the Clinical Education of Novice Physiotherapy Students. International Journal of Practice-based Learning in Health and Social Care. 2015; 3(1):77-93.
7. Blackstock FC, Watson KM, Morris NR, Jones A, Wright A, McMeeken JM, et al. Simulation can contribute a part of cardiorespiratory physiotherapy clinical education: two randomized trials. Simulation in healthcare : journal of the Society for Simulation in Healthcare. 2013; 8(1):32-42.
8. Bogossian F, Cooper S, Kelly M, Levett-Jones T, McKenna L, Slark J, et al. Best practice in clinical simulation education − are we there yet? A cross-sectional survey of simulation in Australian and New Zealand pre-registration nursing education. Collegian. 2018; 25(3):327-34.
9. Bogossian F, McKenna L, Higgins M, Benefer C, Brady S, Fox-Young S, et al. Simulation based learning in Australian midwifery curricula: Results of a national electronic survey. Women & Birth. 2012; 25(2):86-97.
10. Bogossian FE, Cant RP, Ballard EL, Cooper SJ, Levett-Jones TL, McKenna LG, et al. Locating "gold standard" evidence for simulation as a substitute for clinical practice in prelicensure health professional education: A systematic review. Journal of clinical nursing. 2019; 28(21-22):3759-75.
11. Bradley Cynthia S, Johnson Brandon K, Dreifuerst Kristina T, White P, Conde Susan K, Meakim Colleen H, et al. Regulation of Simulation Use in United States Prelicensure Nursing Programs. Clinical Simulation in Nursing. 2019; 33:17-25.
12. Breymier Tonya L, Rutherford-Hemming T, Horsley Trisha L, Atz T, Smith Lisa G, Badowski D, et al. Substitution of Clinical Experience With Simulation in Prelicensure Nursing Programs: A National Survey in the United States. Clinical Simulation in Nursing. 2015; 11(11):472-8.
13. Bridge P, Adeoye J, Edge Christopher N, Garner Vicky L, Humphreys A-L, Ketterer S-J, et al. Simulated Placements as Partial Replacement of Clinical Training Time: A Delphi Consensus Study. Clinical Simulation in Nursing. 2022; 68:42-8.
14. Brien L-A, Charette M, Goudreau J. Nursing Students’ Perceptions of the Contribution of High-Fidelity Simulation and Clinical Placement in a Critical Care Course. Clinical Simulation in Nursing. 2017; 13(9):436-41.
15. Brook J, Kemp C, Abbott S. Evaluation of a virtual placement for mental health nursing students: a pilot study. Journal of Mental Health Training, Education & Practice. 2023; 18(5):366-76.
16. Cant RP, Cooper SJ. Use of simulation-based learning in undergraduate nurse education: An umbrella systematic review. Nurse education today. 2017; 49:63-71.
17. Chircop A, Cobbett S. Gett'n on the bus: evaluation of Sentinel City®3.0 virtual simulation in community/population health clinical placement. International Journal of Nursing Education Scholarship. 2020; 17(1):1-12.
18. Chu Eli Mang Y, Sheppard L, Guinea S, Imms C. Placement replacement: A conceptual framework for designing simulated clinical placement in occupational therapy. Nursing & Health Sciences. 2019; 21(1):4-13.
19. Cieslowski B, Haas T, Oh Kyeung M, Chang K, Oetjen Cheryl A. The Development and Pilot Testing of Immersive Virtual Reality Simulation Training for Prelicensure Nursing Students: A Quasi-Experimental Study. Clinical Simulation in Nursing. 2023; 77:6-12.
20. Cornelius Cynthia A. Simulation usage as a partial replacement of traditional clinical: A study of administrator and faculty perceptions in practical nursing programs in Pennsylvania. 2013.
21. Creagh S, Pigg N, Gordillo C, Banks J. Virtual medical student radiology clerkships during the COVID-19 pandemic: Distancing is not a barrier. Clin Imaging. 2021; 80:420-3.
22. Curl ED, Smith S, Ann C, McGee LA, Das K. Effectiveness of Integrated Simulation and Clinical Experiences Compared to Traditional Clinical Experiences for Nursing Students. Nursing education perspectives. 2016; 37(2):72-7.
23. Curry-Lourenco K, Sherraden Bradley C, White P, Loomis A, Childress Reba M, Waxman KT. Where Are We Now? A Follow-up Survey on Regulation of Simulation Use in United States Prelicensure Nursing Programs. Clinical Simulation in Nursing. 2022; 72:9-14.
24. Dale DC, Perlow ER, Lucado AM. Partial Substitution of Simulation-Based Learning Allows Equal Student Self-confidence in the Acute Care Setting: A Pilot Study. Journal of Acute Care Physical Therapy. 2023; 14(1):10-7.
25. Davis Alice M, LaPorta L, Mulligan Nancy F, Carmel S, Thomas S, O'Dell D. Quality Delivered: How a Pandemic Fostered Innovation and Creative Solutions in Clinical Education. Internet Journal of Allied Health Sciences & Practice. 2022; 20(3):1-20.
26. Dennis D, Cipriano L, Mulvey G, Parkinson S, Reubenson A, Furness A. Observational Study Exploring the Efficacy and Effectiveness of a New Model of Peer-Assisted Simulation-Based Learning Clinical Placement. International Journal of Environmental Research and Public Health. 2022; 19(8).
27. Dolan H, Amidon Brayton J, Gephart Sheila M. Evidentiary and theoretical foundations for virtual simulation in nursing education. Journal of Professional Nursing. 2021; 37(5):810-5.
28. Donovan SK, Herstein JJ, Prober CG, Kolars JC, Gordon JA, Boyers P, et al. Expansion of simulation and extended reality for undergraduate health professions education: A call to action. J Interprof Educ Pract. 2021; 24:100436.
29. Durfee SM, Goldenson RP, Gill RR, Rincon SP, Flower E, Avery LL. Medical Student Education Roadblock Due to COVID-19: Virtual

Radiology Core Clerkship to the Rescue. Acad Radiol. 2020; 27(10):1461-6.
30. Easton C, Seaman Claire E, Patton N, Nott Melissa T, Brown L. Clinical educators' perspectives on transitioning to telesupervision: Experiences piloting a telepractice stuttering simulation placement. Journal of Clinical Practice in Speech-Language Pathology. 2021; 23(3):128-33.
31. Evans N. A safe place to learn about critical care: First-of-its-kind placement offers an immersive experience that builds students' confidence in identifying deteriorating patients. Nursing Standard. 2024; 39(4):33-4.
32. Fehl M, Gehres V, Geier AK, Mundt T, Klinge K, Frese T, et al. Medical students' adoption and evaluation of a completely digital general practice clerkship - cross-sectional survey and cohort comparison with face-to-face teaching. Med Educ Online. 2022; 27(1):2028334.
33. Fitzgerald K, Denning T, Vaughan B. Simulated learning activities as part replacement of clinical placements in osteopathy: A case study. International Journal of Osteopathic Medicine. 2017; 26:44-8.
34. Fitzgerald Kylie M, Denning T, Vaughan Brett R, Fleischmann Michael J, Jolly Brian C. Simulation can offer a sustainable contribution to clinical education in osteopathy. Chiropractic & Manual Therapies. 2019; 27(1):N.PAG-N.PAG.
35. Fogg N, Wilson C, Trinka M, Campbell R, Thomson A, Merritt L, et al. Transitioning from direct care to virtual clinical experiences during the COVID-19 pandemic. Journal of Professional Nursing. 2020; 36(6):685-91.
36. Fung JTC, Zhang W, Yeung MN, Pang MTH, Lam VSF, Chan BKY, et al. Evaluation of students' perceived clinical competence and learning needs following an online virtual simulation education programme with debriefing during the COVID-19 pandemic. Nurs Open. 2021; 8(6):3045-54.
37. Gospodarevskaya E, Carter R, Imms C, Chu EMY, Nicola-Richmond K, Gribble N, et al. Economic evaluation of simulated and traditional clinical placements in occupational therapy education. Australian occupational therapy journal. 2019; 66(3):369-79.
38. Grant T, Thomas Y, Gossman P, Berragan L. The use of simulation in occupational therapy education: A scoping review. Australian Occupational Therapy Journal. 2021; 68(4):345-56.
39. Hansen J, Bratt M. Effect of Sequence of Simulated and Clinical Practicum Learning Experiences on Clinical Competency of Nursing Students. Nurse Educator. 2017; 42(5):231-5.
40. Hayden Jennifer K, Smiley Richard A, Alexander M, Kardong-Edgren S, Jeffries Pamela R. The NCSBN National Simulation Study: A Longitudinal, Randomized, Controlled Study Replacing Clinical Hours with Simulation in Prelicensure Nursing Education. Journal of Nursing Regulation. 2014; 5(2):S3-S40.
41. Hensley JG. A comparison of the effectiveness of clinical simulations using standardized patients with actual hospital experiences in high-risk maternity nursing training. 1996. pp. 78 p- p.
42. Hewat S, Penman A, Davidson B, Baldac S, Howells S, Walters J, et al. A framework to support the development of quality simulation-based learning programmes in speech–language pathology. International Journal of Language & Communication Disorders. 2020; 55(2):287-300.
43. Hill Anne E, Ward E, Heard R, McAllister S, McCabe P, Penman A, et al. Simulation can replace part of speech-language pathology placement time: A randomised controlled trial. International Journal of Speech-Language Pathology. 2021; 23(1):92-102.
44. Hoffmann RL, O'Donnell JM, Kim Y. The effects of human patient simulators on basic knowledge in critical care nursing with undergraduate senior baccalaureate nursing students. Simulation in Healthcare. 2007; 2(2):110-4.
45. Imms C, Froude E, Chu EMY, Sheppard L, Darzins S, Guinea S, et al. Simulated versus traditional occupational therapy placements: A randomised controlled trial. Australian occupational therapy journal. 2018; 65(6):556-64.
46. Jacobs C, McEwen A. Adapting to the challenges of the global pandemic on genetic counselor education: Evaluating students' satisfaction with virtual clinical experiences. Journal of Genetic Counseling. 2021; 30(4):1074-83.
47. Jimenez YA, Gray F, Di Michele L, Said S, Reed W, Kench P. Can simulation-based education or other education interventions replace clinical placement in medical radiation sciences? A narrative review. Radiography. 2023; 29(2):421-7.
48. Joung J, Kang Kyung I. Can Virtual Simulation Replace Clinical Practical Training for Psychiatric Nursing? Issues in Mental Health Nursing. 2022; 43(8):706-11.
49. Judd BK, Alison JA, Waters D, Gordon CJ. Comparison of Psychophysiological Stress in Physiotherapy Students Undertaking Simulation and Hospital-Based Clinical Education. Simul Healthc. 2016; 11(4):271-7.
50. Kaminski-Ozturk N, Martin B. Prelicensure Nursing Clinical Simulation and Regulation During the COVID-19 Pandemic. Journal of Nursing Regulation. 2023; 14(2):36-42.
51. Kaminski-Ozturk N, Martin B. Virtual Clinical Simulation Adoption and Use by Licensed Practical Nurse/Licensed Vocational Nurse Education Programs During the COVID-19 Pandemic. J Nurs Regul. 2023; 14(1):21-9.
52. Kasai H, Shikino K, Saito G, Tsukamoto T, Takahashi Y, Kuriyama A, et al. Alternative approaches for clinical clerkship during the COVID-19 pandemic: online simulated clinical practice for inpatients and outpatients-A mixed method. BMC Med Educ. 2021; 21(1):149.
53. Kelly DG, Brown DS, Perritt L, Gardner DL. A descriptive study comparing achievement of clinical education objectives and clinical performance between students participating in traditional and mock clinics. Journal of Physical Therapy Education (American Physical Therapy Association, Education Section). 1996; 10(1):26-31.
54. Kennedy Daphne M, Jewell Justine J, Hickey Jason E. Male nursing students' experiences of simulation used to replace maternal-child clinical learning in Qatar. Nurse Education Today. 2020; 84:N.PAG-N.PAG.
55. Ketterer SJ, Callender J, Warren M, Al-Samarraie F, Ball B, Calder KA, et al. Simulated versus traditional therapeutic radiography placements: A randomised controlled trial. Radiography. 2020; 26(2):140-6.
56. Killam Laura A, Luctkar-Flude M. Virtual Simulations to Replace Clinical Hours in a Family Assessment Course: Development Using H5P, Gamification, and Student Co-Creation. Clinical Simulation in Nursing. 2021; 57:59-65.
57. Kobeissi M, Christopherson K, Kearney K, Aggarwal Seema S. Expanding Clinical Education During a Time of Crisis: Innovative Virtual Simulation. Clinical Simulation in Nursing. 2021; 56:117-20.
58. Korayem GB, Alboghdadly AM. Integrating simulation into advanced pharmacy practice experience curriculum: An innovative approach to training. Saudi Pharm J. 2020; 28(7):837-43.
59. Kubin L, Fogg N, Trinka M. Transitioning Child Health Clinical Content From Direct Care to Online Instruction. Journal of Nursing Education. 2021; 60(3):177-9.
60. Kubin L, Fogg N, Trinka M. Alternative Clinical Learning Experiences for Nursing Education Using Virtual Individual Patients. Nursing

Education Perspectives (Wolters Kluwer Health). 2023; 44(4):259-60.
61. Kulju Lori A. The Development of a 2-Credit Simulated Specialty Clinical Immersion for 15-Month Senior-Level BSN Students. Clinical Simulation in Nursing. 2020; 49:24-7.
62. Larue C, Pepin J, Allard E. Simulation in preparation or substitution for clinical placement: A systematic review of the literature. Journal of Nursing Education and Practice. 2015; 5.
63. Legge TR, Holthaus A, Hallmark B, Alexander K. Preparing for the Next Generation NCLEX Using a Virtual Simulation. The Journal of nursing education. 2023:1-5.
64. Lobão C, Coelho A, Parola V, Neves H, Sousa Joana P, Gonçalves R. Changes in Clinical Training for Nursing Students during the COVID-19 Pandemic: A Scoping Review. Nursing Reports. 2023; 13(1):378-88.
65. Mancini Mary E, LeFlore Judy L, Cipher Daisha J. Simulation and Clinical Competency in Undergraduate Nursing Programs: A Multisite Prospective Study. Journal of Nursing Education. 2019; 58(10):561-8.
66. Massias LA. Influencing critical thinking in nursing education: comparing high-fidelity simulations and traditional clinical training. 2009. pp. 119 p- p.
67. McCoy Tammy L. Implementing High-fidelity Simulation to Meet Undergraduate Clinical Requirements. Implementing High-fidelity Simulation to Meet Undergraduate Clinical Requirements. 2018:1-.
68. McDermott D, McEwing E, Lopez J, Osso M, Trujillo G. Pandemic Crisis: Simulation Contingency Plans. Teaching & Learning in Nursing. 2021; 16(4):393-5.
69. Meyer MN, Connors H, Hou Q, Gajewski B. The effect of simulation on clinical performance: a junior nursing student clinical comparison study. Simul Healthc. 2011; 6(5):269-77.
70. Morgan K, Wagg A, Purssell E, Kilburn A. Evaluation of a virtual practice placement: A model to increase student capacity. Nurse Education in Practice. 2024; 75:N.PAG-N.PAG.
71. Mulcahy A, Gruben D, Wells-Beede E. Overcoming Resistance: Implementation of 100% Simulation for First Semester Nursing Students. Clinical Simulation in Nursing. 2021; 50:107-11.
72. Nagdee N, Sebothoma B, Madahana M, Khoza-Shangase K, Moroe N. Simulations as a mode of clinical training in healthcare professions: A scoping review to guide planning in speech-language pathology and audiology during the COVID-19 pandemic and beyond. South African Journal of Communication Disorders. 2022; 69(2):1-13.
73. Ng CKC. A review of the impact of the COVID-19 pandemic on pre-registration medical radiation science education. Radiography. 2022; 28(1):222-31.
74. Nguyen W, Fromer I, Remskar M, Zupfer E. Development and Implementation of Video-Recorded Simulation Scenarios to Facilitate Case-Based Learning Discussions for Medical Students' Virtual Anesthesiology Clerkship. MedEdPORTAL. 2023; 19:11306.
75. O'Brien L, Tighe J, Doroud N, Barradell S, Dowling L, Pranata A, et al. "Burnout felt inevitable": Experiences of university staff in educating the nursing and allied health workforce during the first COVID-19 waves. Frontiers in public health. 2023; 11:1082325.
76. Olaussen C, Steindal Simen A, Jelsness-Jørgensen L-P, Aase I, Stenseth Hege V, Tvedt Christine R. Integrating simulation training during clinical practice in nursing homes: an experimental study of nursing students' knowledge acquisition, self-efficacy and learning needs. BMC Nursing. 2022; 21(1):1-11.
77. Parker Ramona A, McNeill J, Howard J. Comparing Pediatric Simulation and Traditional Clinical Experience: Student Perceptions, Learning Outcomes, and Lessons for Faculty. Clinical Simulation in Nursing. 2015; 11(3):188-93.
78. Parker Ramona A, McNeill Jeanette A, Pelayo Lula W, Goei Kathleen A, Howard J, Gunter MD. Pediatric Clinical Simulation: A Pilot Project. Journal of Nursing Education. 2011; 50(2):105-11.
79. Parrott L, Pettit E, Mallinson A, Knox P, Bates S, Callard J. How was it for you? University practice educators' reflections on delivering a creative clinical placement during the COVID-19 pandemic in the UK. Journal of Clinical Practice in Speech-Language Pathology. 2021; 23(3):134-9.
80. Partner A, Shiner N, Hyde E, Errett S. First year student radiographers' perceptions of a one-week simulation-based education package designed to increase clinical placement capacity. Radiography. 2022; 28(3):577-85.
81. Partner, A.; England, A.; Young, R.; Shiner, N.; Bridge, P. Post COVID-19 trends in simulation use within diagnostic radiography and radiation therapy education. Radiography (Lond) Jul 2023; 29(4):684-689.
82. Peachey L, McParland T, Goldsworthy S, Williams V. P Stands for Pivot: Pivoting Face-to-Face Practicum to Virtual Simulation during the Pandemic. Clinical Simulation in Nursing. 2021; 57:53-8.
83. Quail M, Brundage SB, Spitalnick J, Allen PJ, Beilby J. Student self-reported communication skills, knowledge and confidence across standardised patient, virtual and traditional clinical learning environments. BMC Med Educ. 2016; 16:73.
84. Raman S, Labrague Leodoro J, Arulappan J, Natarajan J, Amirtharaj A, Jacob D. Traditional clinical training combined with high-fidelity simulation-based activities improves clinical competency and knowledge among nursing students on a maternity nursing course. Nursing Forum. 2019; 54(3):434-40.
85. Redinger KE, Greene JD. Virtual Emergency Medicine Clerkship Curriculum during the COVID-19 Pandemic: Development, Application, and Outcomes. West J Emerg Med. 2021; 22(3):792-8.
86. Reed H. An Examination of Critical Thinking Skills in Traditional and Simulated Environments for Occupational Therapy Students. 2014.
87. Reid Carol A, Ralph Jody L, El-Masri M, Ziefle K. High-Fidelity Simulation and Clinical Judgment of Nursing Students in a Maternal–Newborn Course. Western Journal of Nursing Research. 2020; 42(10):829-37.
88. Richardson H, Gilmartin MJ, Fulmer T. Shifting the clinical teaching paradigm in undergraduate nursing education to address the nursing faculty shortage. Journal of Nursing Education. 2012; 51(4):226-31.
89. Richardson H, Goldsamt Lloyd A, Simmons J, Gilmartin M, Jeffries Pamela R. Increasing Faculty Capacity: Findings from an Evaluation of Simulation Clinical Teaching. Nursing Education Perspectives (National League for Nursing). 2014; 35(5):308-14.
90. Roberts E, Kaak V, Rolley J. Simulation to Replace Clinical Hours in Nursing: A Meta-narrative Review. Clinical Simulation in Nursing. 2019; 37:5-13.
91. Roberts S, Warren T, Moore Leslie C. COVID-19 Pandemic: Effects of Replacing Clinical Hours With Virtual Simulation in BSN Prelicensure Nursing Education. Nursing Education Perspectives (Wolters Kluwer Health). 2022; 43(5):306-8.
92. Rome C. The Impact of Simulation-Based Learning Experience on Critical Thinking Acquisition. 2012. pp. 146 p- p.
93. Romig BD, Maillet JO, Chute PM, McLaughlin RJ. Clinical education from a specialized accreditation viewpoint: A report of the

ASAHP Clinical Education Task Force. Journal of Allied Health. 2013; 42(3):151-6.

94. Roye J, Anderson M, Diaz Desiree A, Rogers M. Considerations for the Effective Integration of Virtual Simulation in the Undergraduate Nursing Curriculum. Nursing Education Perspectives (Wolters Kluwer Health). 2021; 42(6):E173-E5.

95. Sadd R. Ethical Considerations of Replacing Clinical Hours With Simulation in Undergraduate Nursing Education: Means to an End, or an End in Itself? Creative Nursing. 2023; 29(2):187-91.

96. Salje J, Moyo M. Implementation of a virtual student placement to improve the application of theory to practice. British Journal of Nursing. 2023; 32(9):434-41.

97. Samson J, Gilbey M, Taylor N, Kneafsey R. Virtual Simulated Placements in Healthcare Education: A scoping review. 2023.

98. Sanders Mary K, Barr Jessica L, Goldstein Leigh A. Development and Implementation of Unfolding Pediatric Simulations. Journal of Nursing Education. 2020; 59(2):107-10.

99. Sanderson L, Choma L, Cappelli T, Arrey S, Noonan I, Prescott S, et al. Developing online simulated practice placements: a case study. British Journal of Nursing. 2023; 32(13):636-43.

100. Sanford N, Raleigh M, Dickinson T. A Case Study of a Five-Day Virtual Clinical Simulation with Pre-Licensure Nursing Students. Journal of the American Nurses Association - New York (JANANY). 2023; 3(1):14-21.

101. Schlairet MC, Pollock JW. Equivalence testing of traditional and simulated clinical experiences: undergraduate nursing students' knowledge acquisition. Journal of Nursing Education. 2010; 49(1):43-7.

102. Sears K, Goldsworthy S, Goodman WM. The relationship between simulation in nursing education and medication safety. Journal of Nursing Education. 2010; 49(1):52-5.

103. Sharoff L. Student's Perception of vSim for Nursing® using the Simulation Effectiveness Tool—Modified. Clinical Simulation in Nursing. 2022; 68:1-8.

104. Shea Kathleen L, Rovera Edward J. Preparing for the COVID-19 Pandemic and Its Impact on a Nursing Simulation Curriculum. Journal of Nursing Education. 2021; 60(1):52-5.

105. Sheepway L, Jessup B, Podubinski T, Heaney S, Bailie J, Hoang H, et al. A qualitative exploration of health student perspectives of rural and remote placements during the early stages of the COVID-19 pandemic. Australian Journal of Rural Health. 2023; 31(2):294-307.

106. Smiley R, Martin B. Simulation in Nursing Education: Advancements in Regulation, 2014–2022. Journal of Nursing Regulation. 2023; 14(2):5-9.

107. Smiley Richard A. Survey of Simulation Use in Prelicensure Nursing Programs: Changes and Advancements, 2010–2017. Journal of Nursing Regulation. 2019; 9(4):48-61.

108. Smith JD, Jones PD. The COVID-19 e-lective: using innovation to manage disrupted medical student clinical placements. BMC Med Educ. 2023; 23(1):92.

109. Soccio DA. Effectiveness of Mental Health Simulation in Replacing Traditional Clinical Hours in Baccalaureate Nursing Education. Journal of psychosocial nursing and mental health services. 2017; 55(11):36-43.

110. Squires K, Heaney S, MacDonald-Wicks L, Johnston C, Brown L. Mapping Simulated-Based Learning Experiences Incorporated Into Professional Placements in Allied Health Programs: A Scoping Review. Simulation in healthcare : journal of the Society for Simulation in Healthcare. 2022; 17(6):403-15.

111. Strong Virginia L. Perceptions of graduate nurse practitioner faculty on the use of simulation in lieu of traditional clinical hours. 2023.

112. Tabbakhian K, Melia. The impact of the COVID-19 pandemic on nursing simulation use and the influence of simulation use on future admissions. 2022.

113. Taylor N, Wyres M, Green A, Hennessy-Priest K, Phillips C, Daymond E, et al. Developing and piloting a simulated placement experience for students. British Journal of Nursing. 2021; 30(13):S19-S24.

114. Thompson C, Chidume T, Renfroe K. Replacing community clinical hours with an antepartum simulation-based experience. Journal of Professional Nursing. 2022; 43:27-32.

115. Torain Lettie J. Use of human patient simulators as an alternative teaching tool for clinical site experiences in baccalaureate nursing programs. 2011. pp. 113 p- p.

116. Turner C. Student response to varied instructional methods in level I fieldwork experiences. 2024.

117. Villa S, Janeway H, Preston-Suni K, Vuong A, Calles I, Murphy J, et al. An Emergency Medicine Virtual Clerkship: Made for COVID, Here to Stay. West J Emerg Med. 2021; 23(1):33-9.

118. Walker Robyn G. Assessing learning in simulation experiences in graduate programs for speech-language pathology: A phenomenological study. 2023.

119. Ward Elizabeth C, Caird E, Khanal S, Kularatna S, Byrnes J, Penman A, et al. A cost analysis of a 5-day simulation-based learning program for speech-language pathology student training. International Journal of Speech-Language Pathology. 2022.

120. Watson K, Wright A, Morris N, McMeeken J, Rivett D, Blackstock F, et al. Can simulation replace part of clinical time? Two parallel randomised controlled trials. Medical Education. 2012; 46(7):657-67.

121. Waxman KT, Bowler F, Forneris SG, Kardong-Edgren S, Rizzolo MA. Simulation as a Nursing Education Disrupter. Nursing administration quarterly. 2019; 43(4):300-5.

122. Weston J, Zauche LH. Comparison of Virtual Simulation to Clinical Practice for Prelicensure Nursing Students in Pediatrics. Nurse educator. 2021; 46(5):E95-E8.

123. White P, Champion Jane D. Transitioning Undergraduate Maternal-Newborn and Pediatric Hospital Clinical Experience to Immersive Simulation-Based Education. Clinical Simulation in Nursing. 2021; 61:10-3.

124. Wik V, Barfield S, Cornwall M, Lajoie R. Finding the right balance: student perceptions of using virtual simulation as a community placement. International Journal of Nursing Education Scholarship. 2022; 19(1):1-9.

125. Wilkinson E. Survey of clinical placements within pre-registration diagnostic radiography programmes in the UK and Ireland. Radiography. 2023; 29(1):247-54.

126. Wilkinson E, Cadogan E. Radiographers' perceptions of first year diagnostic radiography students' performance following implementation of a simulation-based education model. Radiography. 2023; 29(4):721-8.

127. Williams B, Brown T, Archer F. Can DVD simulations provide an effective alternative for paramedic clinical placement education? Emergency Medicine Journal. 2009; 26(5):377-81.

128. Williams J, Murphy M, Garrow A. Development of a simulation placement in a pre-registration nursing programme. British Journal of Nursing. 2022; 31(10):549-54.
129. Wood Sarah M. Myotherapy student clinical placements: A review beyond the teaching clinic. Journal of Bodywork & Movement Therapies. 2019; 23(4):695-700.
130. Wright A, Moss P, Dennis DM, Harrold M, Levy S, Furness AL, et al. The influence of a full-time, immersive simulation-based clinical placement on physiotherapy student confidence during the transition to clinical practice. Adv Simul (Lond). 2018; 3:3.
131. Zyniewicz Tiffany L. Simulation as replacement for traditional clinical in pre-licensure nursing education: Outcomes of different ratios of replacement time for traditional clinical with simulation. 2020.

Data extraction instrument

**Simulated Clinical Placements (SCPs) data extraction sheet**	
**Evidence source information**	
Aim/objective of the study	
Population/Participant information	
Sample size<br>○ 0-20<br>○ 20-50<br>○ 50-100<br>○ 100-500<br>○ 500-1000<br>○ 1000-2000<br>○ 2000-3000<br>○ 3000-4000<br>○ 4000-5000<br>○ 5000+	
Field<br>○ Dietetics/Nutrition<br>○ Medicine<br>○ Nursing<br>○ Myotherapy<br>○ Physiotherapy<br>○ Radiography<br>○ Occupational therapy<br>○ Osteopathy<br>○ Paramedicine<br>○ Veterinarians<br>○ Psychology/Counselling<br>○ Genetic Counselling<br>○ Pharmacy<br>○ Social work<br>○ Speech Pathology<br>○ Broad health professionals<br>○ Other health professionals	
Findings of the study	
Country eligible study was conducted in<br>○ Australia<br>○ Canada<br>○ China<br>○ Germany<br>○ Indonesia<br>○ Japan<br>○ New Zealand<br>○ Norway<br>○ Oman<br>○ Saudi Arabia<br>○ South Korea<br>○ Qatar<br>○ United Kingdom<br>○ United States<br>○ Other	

Type of evidence sources<br>○ Interventional<br>▪ Randomised control trial<br>▪ Quasi-experimental<br>▪ Pre-Post study design<br>○ Observational<br>▪ Cross-sectional<br>▪ Case-control<br>▪ Retrospective cohort<br>▪ Prospective cohort<br>○ Qualitative<br>○ Mixed-methods<br>○ Discussion<br>○ Editorial<br>○ Review<br>▪ Narrative<br>▪ Scoping<br>▪ Systematic<br>▪ Umbrella<br>○ Case study<br>○ Dissertation<br>○ Other	
Methods of data collection<br>○ Survey<br>○ Focus groups<br>○ Interviews<br>○ Workshops<br>○ No methods described<br>○ Secondary evidence<br>○ Observation<br>○ Not applicable<br>○ Student assessments<br>○ Other	
**Simulated Clinical Placements**	
Reasonings for why SCP was conducted	
Methods of simulated clinical placement	
Resources used for simulated clinical placement	
Learning objective	
COVID-19	
Definition of simulated clinical placement	
Theoretical underpinning	
Conceptual framework	
Hours of simulated clinical placement	
Percentage of simulation replacement<br>○ 0-25%<br>○ 26-50%<br>○ 51-75%<br>○ 76-100%	
Are students assessed within the SCP?<br>○ Yes<br>○ No<br>○ Planned<br>○ Not applicable	
Student assessments	
Costs of simulated clinical placements<br>- Direct costs<br>- Indirect costs	
Description of evaluation of simulated clinical placement	

Regulatory, legislative, accreditation, or qualification implications	
Strengths of SCP	
Weaknesses of SCP	
Barriers of SCP	
Facilitators of SCP	
Integration into the broader educational and practical ecosystem	
Measurement of outcomes	
**Outcomes**	
Student outcomes<br>- Student Performance Outcomes<br>- Students Preparedness for Practice<br>- Student satisfaction and evaluation of SCP	
Implementation of simulation<br>- Resources requirements and distribution<br>- Implementation and tracking of simulation	
No Outcome / NA	
Simulation modalities<br>○ Online<br>○ In person<br>○ Manikin(s)<br>○ Task trainers<br>○ Standardised/simulated patients/participants<br>○ Virtual reality<br>○ Videos of actual or simulated patients<br>○ Computer-based virtual case scenarios<br>○ Hybrid<br>○ Role play with peers<br>○ Multi-modality	

References

1. World Health Organisation guidelines. Transforming and scaling up health professionals' education and training. Geneva: World Health Organisation; 2013.
2. General Medical Council. Guidance on undergraduate clinical placements. General Medical Council,; 2022.
3. Pilcher J, Goodall H, Jensen C, Huwe V, Jewell C, Reynolds R, et al. Simulation-based learning: It's not just for NRP. Neonatal Network. 2021; 31(5).
4. Gantt LT, Young HM. Appendix 1 Commonly Used Simulation Terms, Terminology, and Medical Abbreviations. Healthcare Simulation: A Guide for Operations Specialists, First Edition. 2015; p. 169–79.
5. Lioce L. Healthcare Simulation Dictionary. 2020.
6. Chu EMY, Sheppard L, Guinea S, Imms C. Placement replacement: A conceptual framework for designing simulated clinical placement in occupational therapy. Nurs Health Sci. 2019; 21(1):4-13.
7. Bordage G. Conceptual frameworks to illuminate and magnify. Med Educ. 2009; 43(4):312-9.
8. Moola S, Munn Z, Tufanaru C, Aromataris E, Sears K, Sfetc R, et al. Chapter 7: Systematic reviews of etiology and risk. In: Aromataris E, Munn Z, editors. JBI Manual for Evidence Synthesis. JBI; 2023.
9. Zhang Y, Coello PA, Brozek J, Wiercioch W, Etxeandia-Ikobaltzeta I, Akl EA, et al. Using patient values and preferences to inform the importance of health outcomes in practice guideline development following the GRADE approach. Health Qual Life Outcomes. 2017; 15(1):52.
10. Reja U, Manfreda KL, Hlebec V, Vehovar V. Open-ended vs. close-ended questions in web questionnaires. Developments in applied statistics. 2003; 19(1):159-77.
11. Kitzinger J. Qualitative research: Introducing focus groups. BMJ. 1995; 311(7000):299-302.
12. Jamshed S. Qualitative research method-interviewing and observation. J Basic Clin Pharm. 2014; 5(4):87-8.

13. Aromataris E, Pearson A. The systematic review: An overview. The American Journal of Nursing. 2014; 114(3):53-8.
14. Higgins JPT, Thomas J, Chandler J, Cumpston M, Li T, Page MJ, et al. Cochrane Handbook for Systematic Reviews of Interventions. 2nd edition ed. Chichester (UK): John Wiley & Sons; 2019.
15. Munn Z, Peters MDJ, Stern C, Tufanaru C, McArthur A, Aromataris E. Systematic review or scoping review? Guidance for authors when choosing between a systematic or scoping review approach. BMC Med Res Methodol. 2018; 18(1):143.
16. Yu HY, Everett EN, Baecher-Lind L, Hampton BS. Feasibility of a Regional Approach to an Obstetrics and Gynecology Transition to Residency Course. R I Med J (2013). 2019; 102(7):51-5.
17. Blackstock FC, Watson KM, Morris NR, Jones A, Wright A, McMeeken JM, et al. Simulation can contribute a part of cardiorespiratory physiotherapy clinical education: two randomized trials. Simul Healthc. 2013; 8(1):32-42.
18. Watson K, Wright A, Morris N, McMeeken J, Rivett D, Blackstock F, et al. Can simulation replace part of clinical time? Two parallel randomised controlled trials. Medical Education. 2012; 46(7):657-67.
19. Edwards S, Tuttle N. Using stakeholder input to inform scenario content: an example from physiotherapy. Advances in Simulation. 2019; 4(1):20.
20. Singh I, Raghuvanshi K. Simulated Learning Environments to Prepare for Clinical Placements: Transition to Placement (T2P). IGI Global; 2018; p. 180-207.
21. Tuttle N. The Use of an Online Adaptive Learning Platform as an Adjunct to Live Simulated Clinical Encounters. 2018; p. 93-105.
22. Tuttle N, Horan SA. The effect of replacing 1 week of content teaching with an intensive simulation-based learning activity on physiotherapy student clinical placement performance. Adv Simul (Lond). 2019; 4(Suppl 1):14.
23. Tuttle N BA. Developing Student Skills to Actively Engage in Feedback Conversations: A Pilot Study. The Internet Journal of Allied Health Sciences and Practice. 2017; 15(4).
24. Ketterer SJ, Callender J, Warren M, Al-Samarraie F, Ball B, Calder KA, et al. Simulated versus traditional therapeutic radiography placements: A randomised controlled trial. Radiography (Lond). 2020; 26(2):140-6.
25. Bridge P, Adeoye J, Edge CN, Garner VL, Humphreys A-L, Ketterer S-J, et al. Simulated Placements as Partial Replacement of Clinical Training Time: A Delphi Consensus Study. Clinical Simulation in Nursing. 2022; 68:42-8.
26. Gunn T, Jones L, Bridge P, Rowntree P, Nissen L. The use of virtual reality simulation to improve technical skill in the undergraduate medical imaging student. Interactive Learning Environments. 2018; 26(5):613-20.
27. Ball B, Kirby M, Ketterer SJ, Mullen E, Howard L, Bridge P. Radiotherapy-specific interprofessional learning through simulation. Radiography (Lond). 2021; 27(1):187-92.
28. Bridge P, Shiner N, Bolderston A, Gunn T, Hazell LJ, Johnson R, et al. International audit of simulation use in pre-registration medical radiation science training. Radiography (Lond). 2021; 27(4):1172-8.
29. Partner A, England A, Young R, Shiner N, Bridge P. Post COVID-19 trends in simulation use within diagnostic radiography and radiation therapy education. Radiography (Lond). 2023; 29(4):684-9.
30. Jacobs C, McEwen A. Adapting to the challenges of the global pandemic on genetic counselor education: Evaluating students' satisfaction with virtual clinical experiences. Journal of Genetic Counseling. 2021; 30(4):1074-83.
31. Jacobs C, McEwen A. Embedding simulation in genetic counselor education from the first week of training: Learning outcomes, standardized clients, and students' satisfaction. J Genet Couns. 2024.
